# Humans actively reconfigure neural task states

**DOI:** 10.1101/2024.09.29.615736

**Published:** 2024-10-13

**Authors:** Harrison Ritz, Aditi Jha, Jonathan Pillow, Nathaniel D. Daw, Jonathan D. Cohen

**Affiliations:** 1 Princeton Neuroscience Institute, Princeton University; 2 Department of Psychology, Princeton University; 3 Department of Statistics, Stanford University

**Keywords:** task switching, electroencephalography, state space model, recurrent neural network

## Abstract

The ability to switch between different tasks is a core component of adaptive cognition, but a mechanistic understanding of this capacity has remained elusive. Longstanding questions over whether task switching requires active preparation remain hotly contested, in large part due to the difficulty of inferring preparatory dynamics from behavior or time-locked neuroimaging. We make progress on this debate by quantifying neural task representations using high-dimensional linear dynamical systems fit to human electroencephalographic recordings. We find that these dynamical systems have high predictive accuracy and reveal neural signatures of active preparation that are shared with task-optimized neural networks. These findings inform a classic debate about how we control our cognition, and offer a promising new paradigm for neuroimaging analysis.

Humans have a remarkable capacity to flexibly change how they perform tasks (1–4), such as switching between speaking different languages. This flexibility reflects a core psychometric component of goal-directed cognition (5) and is a strong indicator of cognitive changes across the lifespan (6, 7) and in mental health (8, 9). Despite the centrality of cognitive flexibility in our mental life, we still lack a mechanistic understanding of how people can rapidly configure task processing to achieve their moment-to-moment goals.

Separate literatures have explored how people flexibly prepare for upcoming decisions or upcoming actions. In cognitive psychology, there is a rich history of research exploring how we switch between tasks, often probed through the performance costs that accompany rapidly switching stimulus-response mappings (switching ‘task sets’; (2)). A longstanding debate in this field hinges on whether these switch costs, and the underlying processes they reflect, are due to ‘passive inertia’ or ‘active reconfiguration’ (3). The inertia hypothesis argues that switch costs largely reflect interference from the previous task set, which passively decays over time (10, 11). The reconfiguration hypothesis argues instead that switch costs reflect active preparation for the upcoming task, which is dependent on cognitive control (1, 12). Previous experiments have used behavior or time-locked neuroimaging to adjudicate between these hypotheses (13–17), but these approaches have limited ability to characterize the underlying dynamics of task preparation.

In parallel, a major ongoing focus in the neuroscience of *motor* control has been understanding how the brain prepares for upcoming motor actions. A major advance in this field has been to use dynamical systems theory to explain how neural populations reach action-specific brain states (18–21). This dynamical framework offers a potential solution for *cognitive* control, aligning with recent dynamical theories of transition between cognitive task states (22–24). While promising, these dynamical formalisms have yet to be fully leveraged to explain how people reach task-specific brain states.

This experiment addresses both of the closely-related questions raised above. First, how can we measure preparatory brain states for cognitive tasks, to adjudicate between task-switching hypotheses? We test the utility of the dynamical systems perspective for answering this question, using a novel application of state space modeling to quantify the large-scale dynamics of human brain activity. Second, do neural dynamics support the inertia or active reconfiguration hypotheses? Using state space modeling, we test competing hypotheses about whether task switching is better described by passive decay or by active reconfiguration. Formalizing these hypotheses using task-optimized recurrent neural networks (RNNs), we find that common dynamical motifs across biological and artificial neural systems support the active reconfiguration of neural task states.

## Traditional sensor-level analyses reveal dynamic task encoding

**Fig. 1.**
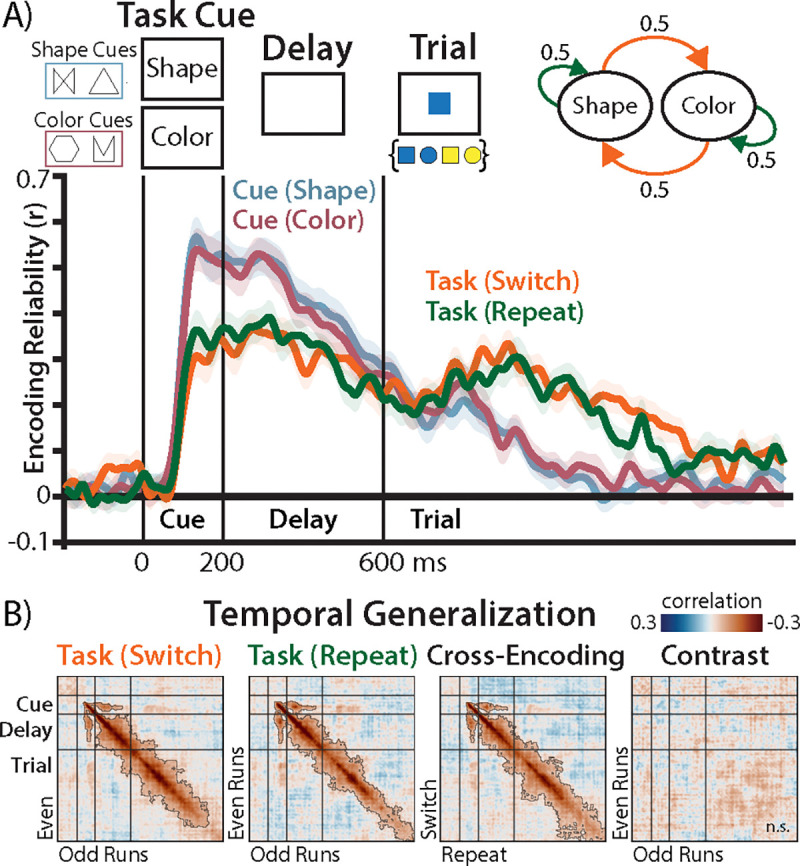
Sensor-level encoding. **A)** Top, participants saw a task cue (two symbols mapped each task), waited through a delay period, then performed the task. Bottom: encoding reliability of cues (red and blue) and tasks (green and orange) during the post-cue period. Traces are smoothed for visualization. Error bars reflect within-participant standard error (SEM; (29)). **B)** Temporal generalization of task patterns, correlating task patterns between different timesteps (and across data splits). For switch and repeat panels, the diagonal is the same as the traces in (A). ‘Cross-Encoding’ depicts the similarity of task patterns between switch and repeat trials. ‘Contrast‘ depicts the difference in encoding strength between switch and repeat trials. Contour indicates *p* < .05, corrected using threshold-free cluster enhancement (TFCE, (30, 31)).

We analyzed scalp electroencephalography (EEG) recordings made in a recent experiment (25), in which thirty participants performed a cued task-switching paradigm (Figure 1A; Method 1 and 2). On each trial, participants were cued whether to perform a ‘shape’ or ‘color’ task, with two potential cues per task. After a delay period, participants saw a colored shape, and responded based on the cued dimension (shape or color) with a button press. The original article found that participants had poorer performance when they had to switch tasks, as well as sensor-level encoding of the task identity during the pre-trial period. This task encoding was interpreted as a preparatory brain state that facilitates task-appropriate behavior (26–28).

We first assessed how well sensor-level EEG analyses differentiated between task-switching hypotheses. We might expect that the inertia hypothesis predicts weaker task encoding on switch trials than repeat trials due to task interference (17, 32), whereas the reconfiguration hypothesis predicts stronger task encoding on switch trials due to the deployment of cognitive control (28, 33). However, these sensor-level predictions are far removed from the underlying neural mechanisms and poorly capture the preparatory dynamics that are central to each theory (i.e., decay or control).

Using encoding geometry analysis (EGA; Method 3; (34)), we found that task identity was robustly encoded throughout the preparatory and trial periods (Figure 1A). However, this sensor-level EGA demonstrated similarly strong task-encoding for switch and repeat trials, inconsistent with both hypotheses (16). To better understand the dynamics of this task encoding, we used EGA to assess temporal generalization (35), measuring the cross-validated similarity between task-encoding patterns at different temporal lags (Method 4). These analyses revealed that tasks representations dramatically changed over the epoch (Figure 1B), evident in their limited off-diagonal similarity, but again without significant differences between switch conditions at any lag.

In summary, sensor-level analyses were unable to discriminate between the inertia and reconfiguration hypotheses, as both predict differences in task encoding between switch and repeat trials. However, traditional encoding analyses like temporal generalization provide limited insight into the underlying preparatory dynamics. To test more specific predictions from our hypotheses for how preparation unfolds over time, we turned to a modeling approach that can characterize the rich temporal structure of the underlying neural system.

## Inferring neural state space dynamics

To characterize the rich dynamics suggested by traditional encoding analyses, we re-analyzed this dataset using linear-Gaussian state space analysis (SSA). Unlike traditional neuroimaging analyses, SSA explicitly accounts for the temporal structure of task representations by modeling the EEG recordings as a linear dynamical system over a set of latent factors.

SSA has long been used in computational neuroscience to model low-dimensional dynamics in multi-unit neural recordings (36–39). This approach provides a parsimonious alternative to EEG analyses using nonlinear (40, 41) or discrete-state (42, 43) dynamical systems. Despite the nonlinearity of neural activation functions, recent work has suggested that linear models can nevertheless serve as effective and highly interpretable descriptive models of neural dynamics (44–46). Critical for our purposes, linear models provide access to a suite of tools from dynamical systems theory and control theory for understanding the effects of recurrent connectivity and inputs (47, 48), and helps to bridge neural and cognitive theories relying on similar dynamical formalisms.

**Fig. 2.**
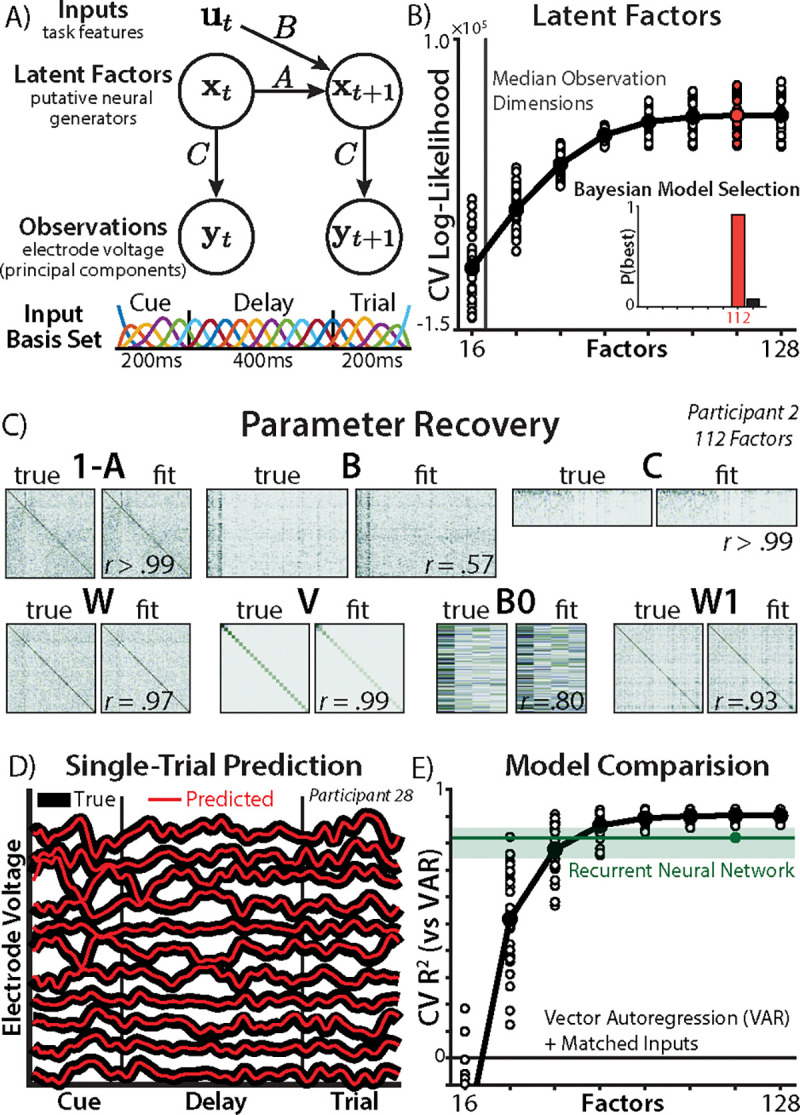
State Space Analysis. **A)** Top : Schematic of the latent state space model. Bottom: temporal basis set spanning the epoch. **B)** The log-likelihood from a held-out test set (y-axis), across different number of factors (x-axis). Dots indicate single subjects; log-likelihood is mean-centered within-participant to show relative differences. Inset: Bayesian model selection. **C)** Parameter recovery on a synthetic dataset generated from participant 2’s parameters. **D)** Predictive accuracy in one example trial from the test set across a subset of the EEG electrodes. Thick black lines indicate EEG voltage, red lines indicate next-timestep predictions from a Kalman filter (112 factor model). **E)** Cross-validated coefficient of determination for SSA models across factor size, relative to autoregressive encoding models (zero indicates equally-good fit). Shaded green line indicates the 95% interval for the performance of RNNs fit to each participant (parameter-matched to 112-factor model).

SSA infers a set of latent factors (state vector x; Figure 2A; Method 5) that evolve according to a standard autoregressive process. Factor dynamics are driven by three components: the recurrence matrix (A), known inputs (with input vector u and input matrix B), and Gaussian noise $\left(w_{t}\sim 𝒩(0,W))\right.$:

(1) $$x_{t}=Ax_{t-1}+Bu_{t-1}+w_{t}$$

To test our core hypothesis about the nature of task encoding on switch versus repeat trials, our model received the current task identity as an input (separately for switch and repeat trials; see Method 6 for full predictor list). We accounted for time-varying neural processes that are below the resolution of EEG by expanding our trial-wise regressors ($r_{n}$) with a smooth temporal basis function ($u_{t,n}=\Phi_{t}⊗r_{n}$; cubic B-spline basis; Figure 2A; Method 6). To model the carry-over from the previous trial, the initial condition depended on the previous task (see Method 6 for predictor list) and initialization noise ($x_{1}=B_{0}u_{0}+w_{1}$).

Factors were assumed to have a linear relationship with the EEG observations (y), ‘read out’ through the observation matrix (C) and corrupted by observation noise $\left(v_{t}\sim 𝒩(0,V))\right.$:

(2) $$y_{t}=Cx_{t}+v_{t}$$

We reduced the dimensionality of the EEG observations during preprocessing using principal components analysis (PCA), retaining 14 – 27 components (99% of the variance). We modeled neural task preparation in each epoch across the cue period, delay period, and the first 200ms of the trial (before any response). We estimated the maximum *a posteriori* parameters of this model through a standard expectation-maximization fitting procedure (Method 3, (49, 50)), implemented in a new toolkit called StateSpaceAnalysis.

## EEG recordings are well-explained by a high-dimensional linear dynamical system

We first assessed the dimensionality of our latent space, choosing the number of factors based on cross-validated predictive accuracy. In systems neuroscience, latent variable models have typically found fewer factors than observation dimensions (51, 52). Surprisingly, we found that SSA models with more factors than the original number of electrodes provided the best cross-validated fit (best model: 112 factors with 90% protected exceedance probability; Figure 2B; (53)). Speculatively, ‘lifting’ the dimensionality may provide a good linear approximation to a nonlinear system (54).

To validate the reliability of this over-parameterized model, we measure SSA’s performance on a synthetic dataset. SSA was highly accurate at recovering the ground truth parameters (Figure 2C; after factor alignment, see Method 6). This strong recovery supports not only the accuracy of SSA as a procedure, but also our ability to differentiate effects across our parameters.

Consistent with this good recovery, our best-fitting model had highly accurate next-timestep prediction (median $R_{CV}^{2}=.98$), evident even in single trials from the test set (Figure 2D). This accuracy was maintained beyond next-step prediction, with good predictions 200 ms into the future (median *t*+200 ms accuracy: $R_{CV}^{2}=.40$; Figure S1A), and simulated power spectra closely matching the empirical spectra (median $R_{CV}^{2}=.98$; Figure S1B; (55)). Finally, model comparison revealed that SSA performed better than both simpler autoregressive encoding models (without latent factors) and non-linear recurrent neural networks (Figure 2E; Method 7). This suggests that SSA strikes a good balance between linear models’ interpretability and non-linear models’ expressivity.

## Latent neural dynamics reveal stable task control

Having validated the predictive power of SSA, we turned to characterizing the dynamics of task preparation. We first assessed whether participants’ latent neural trajectories support an important untested prediction from dynamic theories of task switching: convergence towards a stable, task-specific state that facilitates good performance (22, 23).

To assess the evolution of such task states, we used a procedure known as ‘additive state decomposition’, which factorizes a linear dynamical systems into a set of subsystems for each input ((56); Method 8). We simulated the subsystem for time-varying task inputs ($x_{t}^{\text{task}}=Ax_{t-1}^{\text{task}}+B^{\text{task}}\Phi_{t-1}$), with ‘previous task’ inputs setting the initial conditions.

**Fig. 3.**
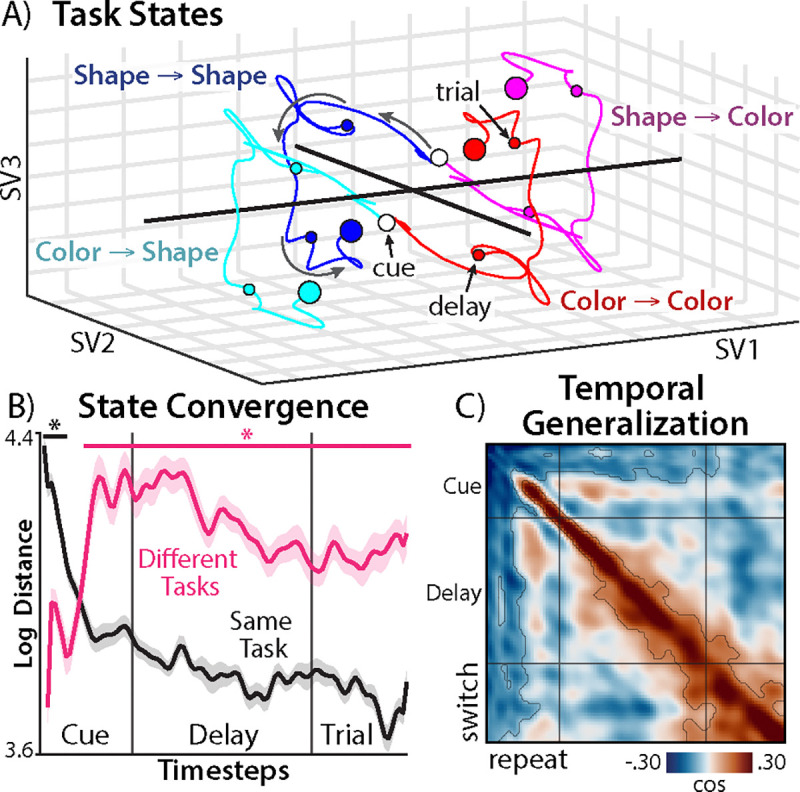
Latent task dynamics. **A)** Simulated task states for each task, split by switch and repeat conditions. Task is contrast-coded, so tasks within the same switch condition are symmetrical. States projected to three dimensions using SVD (‘SV’ refers to singular vectors). **B)** Euclidean distances between task states across switch and repeat conditions. Plotted for same task (black; e.g., blue-cyan in (A)) and different tasks (pink; e.g., red-cyan in (A)). Asterisks indicate *p* < .05 (TFCE-corrected). **C)** Temporal generalization of task states across switch and repeat trials. Vertical and horizontal lines indicate the the boundaries between the cue, delay, and trial periods (Compare to Figure 1B). Contour indicates *p* < .05 (TFCE-corrected).

We visualized the simulated task trajectories using singular value decomposition (SVD) to project participants’ data into a common low-dimensional space (Method 8). We found that the same task appears to have convergent trajectories across switch and repeat conditions (Figure 3A).

To quantify how task states converged over time, we measured the Euclidean distances between states across switch and repeat conditions (Method 8). Whereas brain states for the same task became more similar over time (Figure 3B, black), states for different tasks diverged during the cue period and then maintained separation (Figure 3B, pink). Both of these dynamics support convergence to a task-specific brain state.

Task-switching theories also predict that task-specific states should be stable (e.g., reflecting an attractor state (57)). We quantified the stabilization of task representations using temporal generalization analysis, finding that off-diagonal similarity widened in Figure 3C, in contrast to the uniform generalization seen in sensor-level analyses (see Figure 1B). Testing whether off-diagonal similarity increased over time, we found significant generalization increases for temporal lags up to 248 ms (Figure S2).

Finally, we investigated whether pre-trial task states facilitate performance. Consistent with preparation, we found that participants had faster reaction times on trials for which the EEG signal was better aligned with the task state (summed over timesteps: *d* = −0.53, *p* = .0068; Figure S3; Method 8). Together, these findings support key tenets of dynamical theories of task switching: the brain transitions between stable, task-specific states that promote good performance.

## Task dynamics support active reconfiguration

We found that neural task trajectories supported the core tenets of dynamical task switching theories. We next examined whether these neural dynamics can help discriminate between the inertia and reconfiguration theories of task switching, which we could not distinguish using traditional sensor-level encoding analyses (see Figure 1).

These task switching hypotheses critically differ in how task states evolve during switch and repeat trials. The inertia hypothesis predicts weaker propagation of task information on switch trials than repeat trials, due to passively decaying interference from the previous trial. The reconfiguration hypothesis predicts stronger task propagation on switch trials, due to active changes to the state (i.e., cognitive control).

To measure the propagation of task information, we drew on a metric from control theory called the ‘average controllability Gramian’ (48, 58, 59). The controllability Gramian defines the influences of an input by how much covariance it causes in the system ($G_{\infty }=AG_{\infty }A^{⊤}+BB^{⊤}$; see Method 8), which can be averaged with the matrix trace (48). We adapted this metric to capture the influence of time-varying task inputs over the epoch, which we call the ‘Task Gramian’ (Method 8):

(3) $$G_{t}=AG_{t-1}A^{⊤}+\left(B^{task}\Phi_{t-1}\right){\left(B^{task}\Phi_{t-1}\right)}^{⊤}$$

**Fig. 4.**
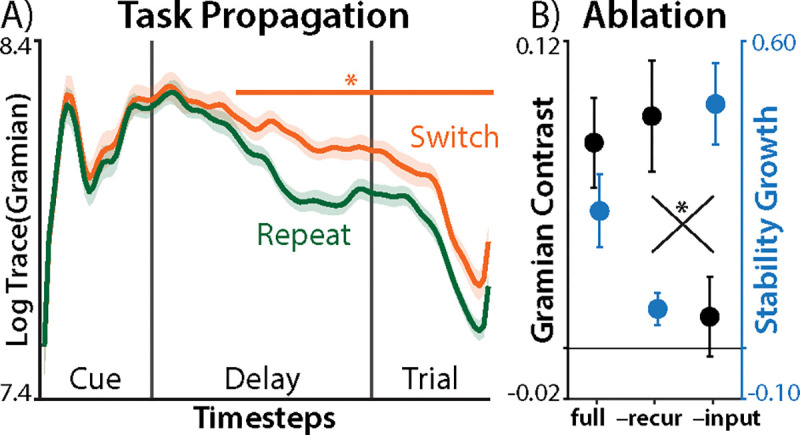
Task Propagation. **A)** The log trace of the Task Gramian, separately for switch (orange) and repeat (green) conditions. Error bars indicate within-participant SEM, asterisks indicates significant differences (*p* < .05, TFCE-corrected). **B)** The contribution of model components toward task propagation (black; contrast between task Gramians for switch and repeat conditions, averaged over timesteps) and task stabilization (blue; linear increase in lagged similarity over the epoch, averaged over first 50 lags; see Figure 2C, Figure S2). ‘full’: full model. ‘–recur’: setting A and $B_{0}$ to zero. ‘–inputs’: z-scoring $B^{\text{task}}\Phi_{t}$ at each timestep. Error bars indicate SEM, asterisks indicate *p* < .05.

To adjudicate between task-switching hypotheses, we tested whether the average Task Gramian was stronger on switch trials or repeat trials. In contrast to our sensor-level analyses, here we found stronger task propagation when participants switched tasks (Figure 4A; summed over timesteps: *d* = 0.83, *p* = 8.6 × 10^−5^), supporting the reconfiguration hypothesis.

Task switching theories make predictions not only about the extent of task propagation, but also the mechanism of this spread. We operationalized passive decay in the inertia hypothesis as how task-dependent initial conditions (i.e., previous task inputs) spread through the system via recurrence (Method 8). We found that setting the fitted initial conditions and recurrence to zero did not reduce the difference in task propagation between switch conditions (Figure 4B, black; Figure S4). However, this ablation greatly reduced the stability growth seen in Figure 2C, consistent with recurrence capturing processes shared by both theories (e.g., attractors).

We operationalized active switching in the reconfiguration hypothesis as the time-varying magnitude of task inputs. Here, we found that z-scoring the fitted task inputs at each timestep strongly reduce differences in task propagation between switch conditions, again supporting the reconfiguration hypothesis.

These task encoding analyses provided consistent support for dynamical theories of task switching in general, and the reconfiguration hypothesis in particular. We found that time-varying inputs appear to move the brain into a stable task state, especially when switching between tasks. We hypothesized that these time-varying task inputs reflect an underlying cognitive control process (23, 26). To facilitate a stronger interpretation of this potential role for control, we formalized the inertia and reconfiguration hypotheses using gated RNNs, which allow us to fully observe task representations. To test these hypotheses, we compared networks’ task-encoding dynamics to those revealed by the EEG analyses reported above.

## Task reconfiguration in recurrent neural networks

We formalized the inertia and reconfiguration hypotheses using RNNs, expressive dynamical systems that can learn to perform basic cognitive tasks (60). Previous neural network models of task switching have implemented inertia and reconfiguration theories through hand-crafted structural mechanisms (22, 61–63), and have not adjudicated between these theories on the basis of their neural predictions. We instead implemented these theories through different training curricula (23, 64), comparing networks’ optimized task-encoding dynamics to human EEG.

**Fig. 5.**
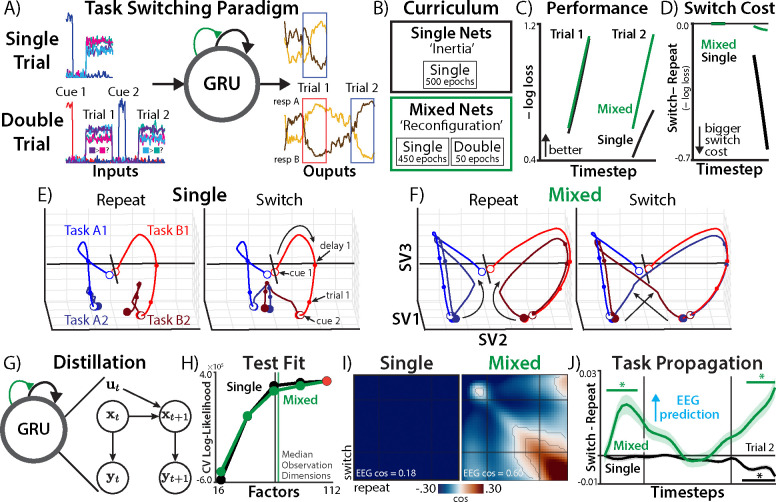
Task dynamics in neural networks. **A)** examples of single trials (top) and double trials (bottom), which differed in whether the required engaging in more than one task in a sequence. **B)** Single networks were only trained on single trials, whereas mixed networks had 10% training on double trials. **C)** network loss over the course of double trial sequences. Error bars are smaller than line width. **D)** Difference in within-sequence loss for switch and repeat trials. **E-F)** Hidden unit activity during double trials, projected to three dimensions with SVD. Note that the SVD was performed across single (D) and mixed (E) networks. White dots are the initial timestep of each trial, colored dots are the final timestep of each trial. **G)** State space analysis of GRU hidden unit trajectories. **H)** Cross-validated log-likelihood across different numbers of SSA latent dimensions. **I)** Temporal generalization of task states across switch and repeat trials. Contour indicates *p* < .05 (TFCE-corrected). Inset statistics are from pixel-wise correlating mean similarity matrices between down-sampled human EEG and each network. **J)** Difference in task propagation between switch and repeat trials. Human EEG found a positive difference (Switch > Repeat). Asterisks reflect differences from zero at *p* < .05 (TFCE-corrected).

We trained networks on a context-dependent decision-making task (65–67), which closely mirrored the EEG task (Method 9). There were two different types of sequences. During ‘single trials’, networks received a task cue, waited through a delay period, and then responded based on the task-relevant stimulus features (Figure 5A, upper). Whereas single trials only had one trial per sequence, during ‘double trials’, networks performed two consecutive trials per sequence (Figure 5A, lower), with equal probability of switching and repeating tasks.

We modeled the two task switching hypotheses through networks’ experience with single and double trials (Figure 5B; Method 9). The inertia hypothesis was captured by ‘single networks’, which had 100% of their training on single trials. We hypothesized that these networks would show passive decay in the absence of learning how to switch. The reconfiguration hypothesis was captured by ‘mixed networks’, which had 90% of their training on single trials and the remaining 10% on double trials. We hypothesized that this limited switching experience would first encourage reconfiguration strategies, before changes to well-learned task representations.

## Switching training produces flexible reconfiguration strategies

We first explored whether single and mixed networks offer face-valid reproductions of the inertia and reconfiguration theories, training 512 RNNs with gated recurrent units (GRUs) under each curriculum (Method 9). Evaluating the networks performance on double trials, we plotted the average loss at each timestep. We found that single and mixed networks had similar performance on the first trial, suggesting similar capacity to perform the tasks (Figure 5C). However, mixed networks showed much stronger performance on the second trial, which they had been trained on. Mixed networks’ advantage on second trials was in part due to better switching, as mixed networks had much lower switch costs for their loss than did single networks (Figure 5D), consistent with learning an adaptive reconfiguration strategy.

We assessed whether mixed networks learned generalizable switching strategies, as one would expect from flexible cognitive control. First, we tested how switching generalized to new sequences. Testing fitted networks on novel ‘triple trials’, we found that the mixed networks still performed better than single networks on untrained third trials (Figure S5). Second, we tested how switching generalized to new tasks. We trained both networks on four tasks, and then trained mixed networks to switch between only two of the tasks (Method 9). After training, we found that mixed networks had better switch-trial performance than single networks on the two tasks that they weren’t trained to switch between, consistent with learning a generalizable reconfiguration strategy (Figure S6).

Finally, we tested the importance of gating mechanisms for switching, given their hypothesized analogy to cognitive control (68, 69). We found that ablating the GRU ‘update’ gate substantially impaired these networks’ ability to learn from double trials (Figure S7), consistent with a role for control in reconfiguration.

## Quantifying RNN reconfiguration with linear dynamical systems

Task-optimized RNNs appear to adopt flexible switching strategies, allowing us to compare networks that only learn to perform tasks (cf. inertia), against networks that learn to actively switch tasks (cf. reconfiguration). A major benefit of RNN modeling is that we have full access to the underlying representations, letting us compare optimized dynamics to the latent trajectories in human EEG.

We first visualized RNNs’ hidden-unit activations using SVD (compare to Figure 3A), projecting a single network and mixed network into a shared low-dimensional space (Method 9). Whereas single and mixed networks exhibited similar first-trial dynamics, their second-trial dynamics sharply diverged. Single networks appeared to move directly between the two trial states, ending up in the middle (Figure 5E). By contrast, mixed networks exhibited much more robust preparatory dynamics (Figure 5F).

To compare the task switching dynamics in each of these networks to human EEG, we used SSA to fit linear dynamical systems to RNNs’ hidden unit activation during preparation for the second trial (Figure 5G; Method 10). Similar to our EEG analyses, we found that high-dimensional latent states improved cross-validated fit (Figure 5H) and provided accurate predictions (one-step $R_{CV}^{2}=.90$; Figure S8). These results showcase how an ‘over-parameterized’ linear model can have high predictive accuracy for nonlinear systems.

We first used temporal generalization analyses to compare the timecourse of task representations across RNNs and EEG (Figure 5I, compare to Figure 2C). In single networks, task states across switch and repeat conditions were negatively aligned throughout the epoch. Although this is inconsistent with the EEG results, this plausibly reflects the inertia hypothesis’s core prediction of cross-task interference. By contrast, temporal generalization in mixed networks was much more similar to human EEG, with growing task stability over time (similarity between temporal generalization matrices: cos(Mixed, EEG) = .60, cos(Single, EEG) = .18; difference *p* < .001; Method 10), supporting the reconfiguration account.

Finally, we tested whether RNNs replicated our EEG signature of stronger task encoding on switch trials than repeat trials. We found that single and mixed networks exhibited opposite patterns (Figure 5J). Single networks, unlike humans, had weaker task propagation on switch trials than repeat trials (summed over timesteps: *d* = −0.091, *p* = .040). Mixed networks, consistent with human EEG, had stronger task encoding on switch trials than repeat trials (*d* = 0.15, *p* = .0010). This concurrence between switching-optimized RNNs and human EEG provides further evidence supporting the reconfiguration account of task switching.

## Discussion

This experiment demonstrates the power of a dynamical systems approach for understanding cognitive flexibility in particular, and the trajectories of neural representations more generally. Linear dynamical systems fit to biological and artificial neural networks provided new insight into the active reconfiguration of neural states, contributing to a decades-old debate in cognitive control.

One prominent feature in both EEG and neural networks was robust configuration for both switch and repeat trials (e.g., Figure 3A and Figure 5F), suggestive of a larger role for active configuration than has typically been assumed (through see (2)). One caveat to this proposal is that the present study had longer inter-trial intervals than are often used. While debates over task switching theories have included paradigms with long delays (3, 70), closely-spaced tasks may reveal addition pressures on preparatory neural activity.

To better characterize the underlying task control process, future work should model the origins of time-varying task inputs. Within a dynamical systems framework, a natural candidate is optimal control theory (48, 58, 59). For cognitive neuroscience more broadly, there is a high demand for more precise spatial localization of the neural generators for M/EEG signals. State space models may provide powerful methods for localizing latent dynamics (71–73), offering a deeper view into the distributed neural systems enabling our cognitive control (74, 75).

## Methods

### 1. EEG Sample

The EEG dataset analyzed here was originally published by Hall-McMaster and colleagues (Hall-McMaster et al., 2019), and was made open-access at https://osf.io/kuzye/ . Thirty participants (18–35 years old; 19 females), with normal or corrected-to-normal vision and no history of neurological or psychological disorders, underwent an EEG recording session during task performance. This study was approved by the Central University Research Ethics Committee at the University of Oxford, with all participants providing informed consent. See (25) for full description.

### 2. EEG Task

Participants performed a cued task-switching experiment during the EEG recording. The major goal of the original study was investigating the role of incentives on task encoding. Our study focused on more traditional switching because this directly relates to our core theoretical questions about task set inertia and reconfiguration.

In the original experiment, the full trial structure involved a cue for high vs low rewards for correct performance (800ms, 400ms delay), a cue for the shape vs color task (200ms, 400ms delay), a trial stimulus consisting of a square or circle colored in yellow or blue (min(RT, 1400ms)), reward feedback (200ms), and an inter-trial interval (1000–1400 ms). Participants’ task was to respond to the trial stimulus according to the cued rule: if the task was ‘shape’, respond with a key press based on the shape; if the task was ‘color’, respond with a key press based on the color. Participants practiced the task until they reached a 70% accuracy criterion, and then performed 10 blocks of 65 trials in the main task. Conditions were balanced within each block. For our encoding and state space analyses, we included trials 1) that were not the first trial of a block, 2) where the current and the previous trial was accurate, 3) where the current RT was longer than 200ms, and 4) that were not identified as containing artifacts during preprocessing. EEG data were recorded with 61 Ag/AgCl sintered electrodes (EasyCap; FieldTrip layout: elec1010), a NeuroScan SynAmps RT amplifier, and Curry 7 acquisition software. See (25) for full description.

### 3. Encoding Geometry Analysis

In our traditional multivariate analyses, we used Encoding Geometry Analysis (EGA; (34)) to quantify task encoding. At each timestep within the epoch, we fit a general linear model (GLM) to the across-trial voltage in the EEG electrodes. Our regression model included an intercept, task identity (with separate regressors for switch and repeat trials), cue identity (with seperate regressor contrasting the cues within each task), cue repetitions vs alternations for repeat trials, the main effect of switch vs repeat, the current trial’s reaction time, and the previous trial’s reaction time. We fit this GLM separately to even and odd runs, and then performed multivariate spatial pre-whitening on our regression estimates (76). We tested the reliability of regression weights by correlating spatial patterns across even and odd folds, and testing these correlations against zero at the group level using sign-flipping permutation tests. This analysis equivalent to out-of-sample prediction (34). To correct for multiple comparisons over time while accounting for temporal autocorrelation, we used threshold-free cluster enhancement (TFCE; (30)) with a set of EEG-optimized parameters (H=2, C=1; from (30); 10,000 permutations).

### 4. Temporal Generalization Analysis

To test the stability of representations over time, we used a variant of temporal generalization analysis (35) that used EGA rather than decoding. Instead of testing the reliability of encoding weights at the same timestep, we tested the similarity of weights across different timesteps (producing time × time estimates of encoding generalization). We tested the generalization against zero using sign-flipping permutation tests (TFCE-corrected; 1,000 permutations).

### 5. State Space Analysis

#### A. Generative Model.

We developed a toolkit called StateSpaceAnalysis for fitting linear-Gaussian state space models (LGSSM) to neuroimaging data. Our goal was to test how effectively LGSSMs could capture rich neuroimaging data, attempting to balance the predictive power of machine learning approaches with the interpretability offered by linear-Gaussian assumptions. To provide the speed and precision needed for estimating the parameters of these large latent variable models, we developed this package using the open-source coding language Julia, available at www.github.com/xxxx .

The generative model for our analysis is a partially observable autoregression process: a linear dynamical system from which we can only take noisy measurements (or alternatively, that only produces noisy emissions of its underlying state). This autoregressive process consistent of a vector of latent factors (x), and a discrete-time difference equation that describes how they evolve:

$$x_{t}=Ax_{t-1}+Bu_{t-1}+w_{t}$$

Latent factors evolve according to their recurrent dynamics (Ax), the influence of known inputs (Bu), and process noise $\left(w_{t}\sim 𝒩(0,W)\right)$. At each timestep, we get a noisy observation of these latent factors:

$$y_{t}=Cx_{t}+v_{t}$$

Observations are projections of the latent factors (Cx) corrupted by observation noise $\left(v_{t}\sim 𝒩(0,V)\right.)$. Note that process noise is carried forward in time through the recurrent dynamics, unlike observation noise. At the beginning of each epoch, we have an expectation about the latent state:

$$x_{1}=B_{0}u_{0}+w_{1}$$

The initial conditions depend on the trial conditions $\left(Bu_{0}\right)$ and initial uncertainty $\left(w_{1}\sim 𝒩\left(0,W_{1}\right))\right.$.

#### B. Expectation-Maximization.

A major benefit of LGSSMs is that they permit a closed-form methods for Bayesian inference of the underlying latent state, as well as a closed-form marginal likelihood of the observed data , $P\left(y_{1:T}|\Theta \right)$ where Θ represents the model parameters. This is achieved using standard inferential tools from control theory that are designed for inference over LGSSMs: Kalman filtering (inferring $x_{t}$ from past observations) and Rauch–Tung–Striebel (RTS) smoothing (inferring $x_{t}$ from both past and future observations).

While the marginal likelihood from the Kalman filter would allow us to directly fit our parameters though techniques like maximum likelihood estimation, in practice it is usually much more efficient to use expectation-maximization (EM; (49, 50, 77)). EM maximizes the expected lower bound of the marginal posterior (often called the evidence lower bound or ELBO) by alternating between two steps. In the E-step, we estimate the latent variables using our RTS smoother. In the M-step, we find a point-estimate of our parameters that maximize the ELBO, given our estimated latent states. With priors on our parameters, this optimizes the mode of their posterior density. The Linear-Gaussian assumptions of our model considerably simplify this procedure by allowing us to work with the sufficient statistics of our estimated latent state (i.e., their mean and covariance).

##### B.1. Marginal Log Posterior.

$$ℒ=E\left[log𝒩\left(x_{1};B_{0}u_{0},W_{1}\right)\right]\;+E\left[log𝒩\left(x_{t};Ax_{t-1}+Bu_{t-1},W\right)\right]+E\left[log𝒩\left(y_{t};Cx_{t},V\right)\right]\;\;+log𝒩\left(A;0,\Lambda_{A}\right)+log𝒩\left(B;0,\Lambda_{B}\right)+log𝒩\left(C;0,\Lambda_{C}\right)$$

##### B.2. E-Step.

Using our filter-smoother algorithm, we estimate the posterior mean $\left(m_{t}\right)$ and covariance $\left(\Sigma_{t}\right)$ of the latent state across timesteps (t) and trials (n), and then the generate the sufficient statistics:

$$M_{\left(t_{1},t_{2}\right)}=\sum_{n=1}^{N}\;\sum_{t=t_{1}}^{t2}\;m_{n,t}m_{n,t}^{⊤}+\Sigma_{t}$$

$$M_{\Delta }=\sum_{n=1}^{N}\;\sum_{t=2}^{T}\;m_{n,t-1}m_{n,t}^{⊤}+\Sigma_{t-1,t}$$

$$Y_{y}=\sum_{n=1}^{N}\;\sum_{t=1}^{T}\;y_{n,t}y_{n,t}^{⊤}$$

$$Y_{\Delta }=\sum_{n=1}^{N}\;\sum_{t=1}^{T}\;m_{n,t}y_{n,t}^{⊤}$$

$$U_{u}=\sum_{n=1}^{N}\;\sum_{t=1}^{T-1}\;u_{n,t}u_{n,t}^{⊤}$$

$$U_{m}=\sum_{n=1}^{N}\;\sum_{t=1}^{T-1}\;m_{n,t}u_{n,t}^{⊤}$$

$$U_{\Delta }=\sum_{n=1}^{N}\;\sum_{t=1}^{T-1}\;m_{n,t+1}u_{n,t}^{⊤}$$

$$U_{u_{0}}=\sum_{n=1}^{N}\;u_{n,0}u_{n,0}^{⊤}$$

$$U_{\Delta_{0}}=\sum_{n=1}^{N}\;m_{n,1}u_{n,0}^{⊤}$$

Note that we can use the same posterior covariances for all trials, which substantially speeds up the computation.

##### B.3. M-Step.

We then use our computed sufficient statistics to update the parameters using a maximization procedure similar to ordinary least squares. Note that we include priors (Λ) on all our dynamics matrices (scaled idenity matrices).

For the dynamics terms:

$$\begin{array}{l} \left[\begin{array}{c} A\\ B \end{array}\right]\leftarrow {\left[\begin{array}{cc} M_{\left(1,T-1\right)}+\Lambda_{A} & U_{m}^{⊤}\\ U_{m} & U_{\left(1,T-1\right)}+\Lambda_{B} \end{array}\right]}^{-1}\left[\begin{array}{c} M_{\Delta }\\ U_{\Delta } \end{array}\right]\\ C\leftarrow {\left(M_{\left(1,T\right)}+\Lambda_{C}\right)}^{-1}Y_{\Delta }\\ B_{0}\leftarrow {\left(U_{u_{0}}+\Lambda_{B_{0}}\right)}^{-1}U_{\Delta_{0}} \end{array}$$

For the covariance terms:

$$\begin{array}{l} W\leftarrow \frac{1}{N(T\;-\;1)\;-\;D_{x}}(M_{\left(2,T\right)}+AM_{\left(1,T-1\right)}A^{⊤}+BU_{m}B^{⊤}-AM_{\Delta }-M_{\Delta }^{⊤}A-BU_{\Delta }-U_{\Delta }^{⊤}B\\ \;\left.\;+\;BU_{m}A^{⊤}+AU_{m}^{⊤}B^{⊤}+A\Lambda_{A}A^{⊤}+B\Lambda_{B}B^{⊤}\right)\\ B\leftarrow \frac{1}{NT\;-\;D_{y}}\left(Y_{y}+CM_{\left(1,T\right)}C^{⊤}-CY_{\Delta }-Y_{\Delta }^{⊤}C+C\Lambda_{C}C^{⊤}\right)\\ W_{0}\leftarrow \frac{1}{N\;-\;D_{x}}\left(M_{\left(1,1\right)}+B_{0}U_{u_{0}}B_{0}^{⊤}-B_{0}U_{\Delta_{0}}-U_{\Delta_{0}}^{⊤}B_{0}+B_{0}\Lambda_{B_{0}}B_{0}^{⊤}\right) \end{array}$$

With $D_{x}$ and $D_{y}$ indicates the numbers of factors and observation dimensions, respectively. Note that our estimator of $W_{0}$ is a little unusual, but is equivalent to (49) when the only input is an intercept term. Alternating between these E-steps and M-steps monotonically improves the ELBO towards a local optimum (i.e., depends on our initialization; see ‘Subspace Identification’ below). Our toolkit is inspired by the SSM and dynamax python packages, but has several optimizations specifically tailored for linear-Gaussian systems.

#### C. Subspace Identification.

We found that good initialization of our parameters was critical to the effectiveness and stability of our model-fitting procedure. We initialized our parameters using a procedure called subspace identification (SSID; (78, 79)). This procedure constructs a large delay embedding matrix which concatenates lagged future and past copies of our observations (y) and inputs (u). The ‘horizon’ of these lags was set to match the largest tested model (see below). We used the canonical variate analysis procedure (80), which uses SVD to find low-dimensional mappings between lagged inputs and outputs. We can recover the A and C matrices by processing different blocks of this SVD, and then estimate the rest of the parameters using fixed-gain Kalman filtering. We performed CVA using a modified version of the ControlSytemIdentification.jl Julia package Method 11.

### 6. Fitting procedure

#### A. Preprocessing.

We split our data into training and testing folds, preprocessing the inputs and observations of each separately. We epoched the task around the prepatory period, including the cue, the delay period, and the first 200ms of the trial (before any RTs had occured).

We used the same inputs as our sensor-level encoding model (see above). For the initial conditions, we included an intercept, the previous task, the previous reaction time, and the upcoming reaction time. We convolved our predictors with a cubic B-spline basis set. Spline knots were placed to optimize coverage at every 5 timesteps. Since we had 100 timesteps in EEG, this resulted in 20 temporal bases per predictor, tiling the entire epoch. Each input was z-scored across trials to reduce collinearity.

We preprocessed the EEG electrodes for SSA using PCA, projecting the voltage timeseries into PCs accounting for 99% of the variance. We used PCA because the observations were degenerate from spatial proximity, independent components analysis, and bad-channel interpolation. PCA also reduced the computational demands of our analyses, due to a smaller observation dimensionality and because it made our observation covariance close to diagonal. We estimated the PCs on training data, and then used these components to project the test data.

#### B. SSID.

To provide the best initialization across our latent dimension hyper-parameter, we estimated SSID for a horizon and latent dimensionality that matched our largest tested model (128 latent factors). We then truncated these systems for smaller models (as latent states from CVA are ordered by their singular values). We found that SSID was enhanced by reshaping our trial-wise data into one long timeseries (allowing for larger numbers of factors, and presumably low frequencies), as well as by only including inputs in the initial timesteps of each epoch (presumably reducing the collinearity between lagged inputs). Note that test data we removed before this process (which were on separate experimental blocks to minimize temporal proximity), and our EM procedure was fit within each epoch. In practice, we found that this procedure provides a good initial guess of the parameters without requiring multiple initial parameters, and was critical for stable estimation of large system (e.g., with more latent dimension that observed dimensions).

#### C. EM.

As described above, our EM procedure cycled between estimating the latent states, and updating the parameters. We fit our model across eight different levels of latent dimensionality: (16, 32, 48, 64, 80, 96, 112, 128). We set the maximum number of iterations to 20,000, measuring the test-set log-likelihood every 100 iterations. We terminated our fitting if either the total data likelihood in the training set stopped decreasing, or if the test log-likelihood stopped decreasing.

#### D. Parameter Recovery.

We validated that our combination of model, data, and fitting procedure could produce identifiable parameter estimates when we know the generative model class. First, we used the LGSSM generative model to create a synthetic dataset based on a participant’s parameters and inputs. We then performed SSA on this synthetic dataset. Next, we aligned our estimated and recovered parameters, as the estimates from SSA are only identifiable up to an invertible transformation. Intuitively, we could shuffle the ordering of the latent factors without changing the likelihood. Finally, we correlated the generating and recovered parameters (correlating the Cholesky factors for covariance matrices). While we had somewhat poorer recovery of input matrices (Figure 2C), this strongly depend on the norm of the input matrix column that we were trying to recover (i.e., some predictors were weakly encoded, and these were difficult to recover).

### 7. Model Comparison

#### A. Bayesian Model Selection.

We selected the number of latent dimensions for subsequent analyses using a standard Bayesian model selection procedure (53). This analysis estimates the expected probability of each model in the population. From these posterior mixture weights, once can compute a ‘protected exceedance probability’, the probability that a model is the most popular within the tested set of models, relative to chance.

#### B. Sensor-Level Null Models.

To compare SSA to a set of simpler null hypotheses, we fit a set of four models directly to the electrode PCs (i.e., without a latent embedding beyond the PCA). First was an intercept-only model (for standard $R^{2}$). Second was an encoding analysis using our full temporal basis set (a generalized additive model similar to EEG methods like the unfold toolbox, (81)). Third was a vector autoregressive (VAR(1)) model, estimating the multivariate relationship between sensor PC responses on adjacent timesteps. Fourth was a model that incorporated both encoding and autoregression. Model four, which included both VAR and encoding, was the best-performing of these null models, so we used this as our benchmark in Figure 2D. We calculated the Cox-Snell $R^{2}$, which uses likelihoods instead of squared errors. This metric is equivalent for normal models with stationary residual covariance (82)), but in our case lets our fit measure account for the predicted covariance.

#### C. Neural-Optimized Recurrent Neural Networks.

To compare SSA to a more complex non-linear state space model, we predicted the PC timeseries used an recurrent neural network (RNN) autoencoder implemented in PyTorch (83)). Each participant was fit with an RNN that had the same number of parameters as their 112-factor SSA model (e.g., to account for SSA’s parameterized covariance matrices). The inputs to this RNN were the previous PC responses and the same set of inputs as our SSA model (formatted as constant inputs over each epoch, which fit better). These inputs were linearly projected into a set of hidden units, along with the previous hidden state, and then passed through a rectified linear unit (ReLU) activation function. The PC response on the next time was linearly decoded from the hidden sate using the transpose of the observation embedding layer (a ‘tied’ parameterization, that matches the assumptions of SSA; (84)). We found similar performance when we fit both the encoding and decoding layers, as these required more parameters. We also fit an initial hidden state, and included the first timestep in our loss.

We fit 32 random initializations of the RNN to each participant. Each fitting session involved 1000 epochs of updating the parameters by backpropagating through time the mean square error of the next-timestep predcition. Each batch contained all of the training data. Parameters were updated using the ‘AdamW’ learning rule (learning rate = .001, weight decay = .01; (85)). To evaluate each model’s performance, we took the best test-set loss across all epochs and initializations.

### 8. Task State Analysis

#### A. Additive state decomposition.

To isolate the dynamics of task encoding, we leveraged a powerful property of linear systems: the superposition principle. Linear state space models can be factorized into an additive set of subsystems for each input, a procedure known as ‘additive state decomposition’ (56). We used this principle to model ‘task subsystems’ in which state dynamics are only caused by task-related inputs. We modeled task inputs separately for switch and repeat trials, allowing us to further decompose our system into a switch-task subsystem and a repeat-task subsystem. Within each subsystem, the state evolves accord to:

$$x_{t}^{\text{input}}=Ax_{t-1}^{\text{input}}+B^{\text{task}}\Phi_{t-1}$$

With $B^{\text{task}}\Phi$ reflecting the estimated input, calculated from the dot product between the columns of B corresponding to a specific input (e.g., task) and our temporal basis set (i.e., the time-varying inputs for task=1). Since we contrast-coded our tasks (+1/−1), the dynamics for each task are symmetrical around the origin. For the initial conditions of this task subsystem, we used the ‘previous task’ component of our initial conditions, which is symmetrical between switch and repeat trials for the same task. To normalize task states across participants, we whitened the latent space by transforming our parameters by the inverse of the mean-field state noise ($Diagonal{\left(W\right)}^{-1}$).

#### B. Spatiotemporal Visualization.

Using the decomposition and symmetry principles afforded by our linear system, we compared the estimated latent trajectories of task encoding between switch and repeat trials. We first visualized task states using group-level singular value decomposition (SVD). The goal of this visualization method was to provide an aggregate task state that accommodated the different realizations across participants (i.e., that aligned their latent states). In both of our the spatial and temporal visualizations, our use of SVD makes the sign of these visualization arbitrary.

To visualize the latent trajectories over time, we spatially concatenated participants’ switch and repeat task trajectories into a wide 2D ‘temporal’ matrix. We projected this temporal matrix into its left singular vectors (weighted by their singular values):

$$\begin{array}{l} x_{\text{temporal}}\in R^{\left(\text{switch}\;\text{timesteps}+\text{repeat}\;\text{timesteps})\times (\text{factors}\times \text{participants}\right)}\\ USV^{⊤}=x_{\text{temporal}}\\ z_{\text{temporal}}=x_{\text{temporal}}VS \end{array}$$

This resulted in separate component timeseries for switch and repeat subsystems. For illustration purposes, we plotted the symmetrical trajectories for each task.

#### C. Task Similarity Analyses.

To quantify our task state visualizations, we took advantage of similar multivariate analysis tools as in our EEG analysis. We performed temporal generalization analysis on the similarity between task representations on switch and repeat subsystems by taking the cosine of the angle between task states across temporal lags. We also measured the proximity of task states across switch and repeat subsystems. We computed the Euclidean distance between either the same task across switch and repeat subsystems, or between different tasks across these subsystems.

To test whether the temporal generalization of task encoding increases over the epoch, we extracted the off-main diagonals of the similarity matrix, which reflect task similarity at different temporal lags. We averaged the diagonals above and below the main diagonal, and then correlated them with a linear time vector (i.e., tested whether the similarity at a given lag increases or decreases over timesteps). After computing these correlations for each participant, we tested for a group-level difference from zero using TFCE.

#### D. Task Propagation.

To quantify the magnitude of task representations, we used the control theoretic tools from the analysis of controllability (48, 58, 86). In control theory, the controllability quantifies the strength of of input-state coupling. For a time-invariant system, the Gramian matrix defines the asymptotic state covariance that is attributable to a (white noise) input:

$$G_{\infty }\;=AG_{\infty }A^{⊤}+BB^{⊤}\;\;=\sum_{t=0}^{\infty }\;\;A^{t}BB^{⊤}A^{t⊤}$$

To capture the influence of a particular set of time-varying inputs, we simulated the trajectory of this Gramian using a matrix-variate difference equation:

$$G_{t}=AG_{t-1}A+\left(B^{\text{task}}\Phi_{t-1}\right){\left(B^{\text{task}}\Phi_{t-1}\right)}^{⊤}\;G_{1}=(B_{0}^{\text{prevTask}}){\left(B_{0}^{\text{prevTask}}\right)}^{⊤}$$

This task-dependent Gramian is equivalent to the cumulative covariance for white noise inputs, measuring how much the system changes due to inputs. It is similar in spirit to the 2-norm of the task state (45), but takes into account the full covariance across factors and the cumulative state change (not just the distance from the origin). We summarized task strength using a standard metric of ‘average controllability’ (48), taking the trace of G each timestep (i.e., the sum of G’s eigenvalues).

#### E. Model Ablation.

We eliminated different components of our fitted model to assess their contributions to our task state trajectories. We assessed the role of system recurrence by setting the recurrence matrix (*A*) and initial conditions ($B_{0}u_{0}^{\text{PreviousTask}}$) to zero. We assessed the role of input strength by z-scoring $Bu_{t}^{\text{task}}$ at each timestep.

#### F. Performance Alignment.

To test whether our inferred task states mattered for good performance, we explored how trial-level brain states predicted upcoming reaction times. First, we projected each participants’ task trajectory for switch and repeat subsystems back into the observation space $(\left.Cx^{\text{task}}\right)$. On every trial and timestep, we computed the cosine similarity between the task state and the brain response. We then put this similarity into a regression along with predictors accounting for task, switch trials, task × switch interaction, and the 2-norm of the neural response. We tested whether EEG-task alignment was significantly different from zero at the group level using TFCE.

### 9. Task-Optimized RNNs

#### A. Task.

We trained networks to perform an extension of the ‘context-dependent decision-making’ task (CDM; (65–67, 87), which was designed to be highly similar to the task that participants performed during EEG.

We trained networks on two kinds of sequences. On ‘single trials’, networks performed a classic CDM task. In each sequence, networks first got a task cue input (one-hot coded; 10 timesteps), followed by a delay period (20 timesteps). They then got two pairs of stimulus inputs (40 timesteps), having to judge which input in the task-relevant pair had higher amplitude. We trained the networks using a logistic loss function on the correct and incorrect response options $\left(-(y\;log(\overset{ˆ}{y})+(1-y)log(1-\overset{ˆ}{y}))\right)$.

On ‘double trials’, networks performed two consecutive trials (i.e., without resetting the state or gradients). They performed the first trial, had a short inter-trial interval (20 timesteps) and then performed the second trial. Sequences within an epoch had a equal number of target repetitions, distractor repetitions, and task repetitions (64 conditions).

Each epoch contained 150 conditions sets (9600 sequences), each with normally distributed noise (cue d’ = 1.0, stimulus d’ = 0.15). We trained at relatively low SNRs to pressure the networks to find task-relevant states.

#### B. Network Architecture.

We trained RNNs with gated recurrent units (GRUs; 108 hidden units; (88)) to perform task-switching. These networks had a standard RNN architecture: linearly encoded inputs, a recurrent hidden state with hyperbolic tangent activation function, and linear decoded outputs. The GRU component of these models learn a pair of ‘gates’, each of which encode the hidden state and inputs, and output a sigmoid that is element-wise multiplied with hidden states. The ‘reset gate’ is applied to the hidden state before it is combined with the inputs and passed through the activation function to produce the ‘proposal state’. The update gate mixes the proposal state and the previous hidden state. We trained GRUs using backpropagation through time, using the AdamW learning rule (learning rate = .01, weight decay = .01).

#### C. Curricula.

We trained two groups of GRUs on different curricula. ‘Single networks’ only trained on the single trials (500 epochs). ‘Mixed networks’ trained on single trials for 90% of their training (450 epochs), and then double trials for the final 10% (50 epochs). The rationale for this design was to teach both groups how to perform the task, but give the Mixed networks enough experience that they would learn a compensatory strategy for switching. Note that 450 epoch constituted over-training on the task, at a point where the ‘test loss’ (noise-free loss) was increasing (Figure S7). We trained 512 networks in each group, using matched random seed to ensure that training was the same for the first 90% of epochs.

#### D. Performance Evaluation.

Networks were tested on noise-free double trials to evaluate whether they had learned the underlying task structure and could switch between tasks. We measured the within-sequence performance through the per-timestep loss, averaged over conditions and log transformed. To test for temporal generalization, we evaluated networks on ‘triple trials’, which included a third trial that was absent from training.

#### E. Task Generalization.

To test the generality of mixed networks’ capacity to switch between task, we explored a curriculum for assessing abstract flexibility. In this curriculum, single trials involved learning four different tasks (mapped to two responses). In double trials, networks switched and repeated only two out of the four tasks. Single networks performed 1000 epochs of single trials, and mixed networks performed 900 single and 100 double. We then assessed both networks’ switching ability on double trials containing the two tasks that had been held out of switching practice, comparing the timestep-resolved loss on switch and repeat trials.

#### F. Gate Ablations.

To assess the role of the reset and update gates in GRU’s performance, we trained a series of model ablations. We either fixed the reset gate to be open, fixed the update gate to be open, or trained a vanilla RNN. GRUs were trained with tanh activation function and learning rate = .01, whereas the RNN was trained with a ReLU activation function and learning rate = .001 (which improved its performance). Free parameters were matched across networks (108 hidden units for full model, 134 units for ablated models, 190 units for RNN). We trained these models on 500 epochs of the single trials, and then 500 epochs of the double trials.

#### G. Hidden Unit Visualization.

We used a similar group SVD procedure to our EEG (see Method 8) to visualize hidden unit activation during double trials. Using trained networks, we first simulated a set of noisy trials (n=2048) for a single and mixed network (same random seed). Next, we concatenated the hidden unit activations across sequences and networks into a joint matrix ((timesteps × sequences) × ( single-network hidden units + mixed-network hidden units)) Performing SVD on this matrix provides embeddings that captures the common and distinct components of the hidden unit activation, and in practice it produces quite similar trajectories across the two networks. We then averaged the sequences within each task pair and projected these average sequences into the (weighted) right singular vectors corresponding to each network.

### 10. Network Distillation

We used our SSA package to distill trained RNNs into a high-dimensional state space model, allowing us to compare the task dynamics to human participants. Our fitting procedure was closely matched between networks and EEG. We preprocessed training and test trials in a similar way (1536 trials for training, 512 for testing): performing PCA on the hidden unit trajectories (keeping components accounting for 99% of the variance) and constructing a temporal basis for the inputs (10 spline bases over the epoch). Network inputs were a bias, task on switch trials, task on repeat trials, and the main effect of switch. Inputs to initial conditions were a bias and the previous task. We analyzed the preparation preparation for the second trial: cue period (10 timesteps), delay period (20 timesteps), and the initial trial period (10 timesteps).

We fit SSA across five levels of latent dimensionality (16, 40, 64, 88, 112), initializing our parameters with SSID at a horizon of 112. We ran SSA for all 512 single networks and mixed networks. Networks were evaluated using the same methods as human participants.

To evaluate the similarity between temporal generalization matrices in networks and humans, we subsampled human task trajectories to match the duration of networks’ trajectories, averaged the temporal generalization within humans and RNNs, and then computed the cosine similarity between these (vectorized) matrices. Note that we found qualitatively similar results evaluating similarities on a per-network basis.

### 11. Software and Visualization

1. Programming Languages
  - Julia (v1.10.4)
  - Python (v3.10.13)
  - Matlab (R2023b)
2. Software Packages
  - PyTorch (v2.4.0; pytorch.org)
  - MatlabTFCE (github.com/markallenthornton/MatlabTFCE)
  - ControlSystemIdentification.jl (v2.10.2; github.com/baggepinnen/ControlSystemIdentification.jl)
  - FieldTrip (89)
  - EEGLAB (v2024.2; (90))
3. Visualization
  - HenriquesLab bioRxiv template (overleaf.com/latex/templates/henriqueslab-biorxiv-template/nyprsybwffws)
  - Scientific Color Maps (91)

The author(s) are pleased to acknowledge that the work reported on in this paper was substantially performed using the Princeton Research Computing resources at Princeton University which is consortium of groups led by the Princeton Institute for Computational Science and Engineering (PICSciE) and Office of Information Technology’s Research Computing.

## Supplementary Material

Supplement 1

## References

[1] Monsell Stephen. Task switching. Trends Cogn. Sci., 7(3):134–140, March 2003. 12639695 10.1016/s1364-6613(03)00028-7

[2] Kiesel Andrea, Steinhauser Marco, Wendt Mike, Falkenstein Michael, Jost Kerstin, Philipp Andrea M, and Koch Iring. Control and interference in task switching—a review. Psychol. Bull., 136(5):849–874, September 2010. 20804238 10.1037/a0019842

[3] Vandierendonck André, Liefooghe Baptist, and Verbruggen Frederick. Task switching: interplay of reconfiguration and interference control. Psychol. Bull., 136(4):601–626, July 2010. 20565170 10.1037/a0019791

[4] Musslick Sebastian and Cohen Jonathan D. Rationalizing constraints on the capacity for cognitive control. Trends Cogn. Sci., 0(0), July 2021. 10.1016/j.tics.2021.06.001 34332856

[5] Friedman Naomi P and Miyake Akira. Unity and diversity of executive functions: Individual differences as a window on cognitive structure. Cortex, 86:186–204, January 2017. 27251123 10.1016/j.cortex.2016.04.023 PMC5104682

[6] Cepeda Nicholas J, Kramer Arthur F, and Gonzalez de Sather Jessica C M. Changes in executive control across the life span: Examination of task-switching performance. Dev. Psychol., 37(5):715–730, 2001. 11552766

[7] Steyvers Mark, Hawkins Guy E, Karayanidis Frini, and Brown Scott D. A large-scale analysis of task switching practice effects across the lifespan. Proc. Natl. Acad. Sci. U. S. A., August 2019. 10.1073/pnas.1906788116 PMC6731761 31427513

[8] Millan Mark J, Agid Yves, Brüne Martin, Bullmore Edward T, Carter Cameron S, Clayton Nicola S, Connor Richard, Davis Sabrina, Deakin Bill, DeRubeis Robert J, Dubois Bruno, Geyer Mark A, Goodwin Guy M, Gorwood Philip, Jay Thérèse M, Joëls Marian, Mansuy Isabelle M, Meyer-Lindenberg Andreas, Murphy Declan, Rolls Edmund, Saletu Bernd, Spedding Michael, Sweeney John, Whittington Miles, and Young Larry J. Cognitive dysfunction in psychiatric disorders: characteristics, causes and the quest for improved therapy. Nat. Rev. Drug Discov., 11(2):141–168, February 2012. 22293568 10.1038/nrd3628

[9] Snyder Hannah R. Major depressive disorder is associated with broad impairments on neuropsychological measures of executive function: a meta-analysis and review. Psychol. Bull., 139(1):81–132, January 2013. 22642228 10.1037/a0028727 PMC3436964

[10] Allport A, Styles E A, and Hsieh S L. Shifting intentional set - exploring the dynamic control of tasks. In ATTENTION AND PERFORMANCE XV: CONSCIOUS AND NONCONSCIOUS INFORMATION PROCESSING, pages 421–452. MIT Press, London, England, 1994.

[11] Wylie Glenn and Allport Alan. Task switching and the measurement of “switch costs”. Psychol. Res., 63(3):212–233, August 2000. 11004877 10.1007/s004269900003

[12] Rogers Robert D and Monsell Stephen. Costs of a predictible switch between simple cognitive tasks. J. Exp. Psychol. Gen., 124(2):207, 1995.

[13] Meiran Nachshon. Reconfiguration of processing mode prior to task performance. J. Exp. Psychol. Learn. Mem. Cogn., 22(6):1423–1442, November 1996.

[14] Yeung Nick and Monsell Stephen. Switching between tasks of unequal familiarity: the role of stimulus-attribute and response-set selection. J. Exp. Psychol. Hum. Percept. Perform., 29(2):455–469, April 2003. 12760628 10.1037/0096-1523.29.2.455

[15] Schmitz Florian and Voss Andreas. Decomposing task-switching costs with the diffusion model. J. Exp. Psychol. Hum. Percept. Perform., 38(1):222–250, February 2012. 22060144 10.1037/a0026003

[16] Loose Lasse S, Wisniewski David, Rusconi Marco, Goschke Thomas, and Haynes John-Dylan. Switch-independent task representations in frontal and parietal cortex. J. Neurosci., 37(33):8033–8042, August 2017. 28729441 10.1523/JNEUROSCI.3656-16.2017 PMC6596903

[17] Qiao Lei, Zhang Lijie, Chen Antao, and Egner Tobias. Dynamic trial-by-trial recoding of task-set representations in the frontoparietal cortex mediates behavioral flexibility. J. Neurosci., 37(45):11037–11050, November 2017. 28972126 10.1523/JNEUROSCI.0935-17.2017 PMC5678027

[18] Shenoy Krishna V, Sahani Maneesh, and Churchland Mark M. Cortical control of arm movements: a dynamical systems perspective. Annu. Rev. Neurosci., 36:337–359, July 2013. 23725001 10.1146/annurev-neuro-062111-150509

[19] Michaels Jonathan A, Dann Benjamin, and Scherberger Hansjörg. Neural population dynamics during reaching are better explained by a dynamical system than representational tuning. PLoS Comput. Biol., 12(11):e1005175, November 2016. 27814352 10.1371/journal.pcbi.1005175 PMC5096671

[20] Schimel Marine, Kao Ta-Chu, and Hennequin Guillaume. When and why does motor preparation arise in recurrent neural network models of motor control? August 2023. 10.7554/eLife.89131 PMC11421851 39316044

[21] Churchland Mark M and Shenoy Krishna V. Preparatory activity and the expansive null-space. Nat. Rev. Neurosci., 25(4):213–236, April 2024. 38443626 10.1038/s41583-024-00796-z PMC13271173

[22] Ardid Salva and Wang Xiao-Jing. A tweaking principle for executive control: neuronal circuit mechanism for rule-based task switching and conflict resolution. J. Neurosci., 33(50):19504–19517, December 2013. 24336717 10.1523/JNEUROSCI.1356-13.2013 PMC6618764

[23] Musslick Sebastian, Jang Seong Jun, Shvartsman Michael, Shenhav Amitai, and Cohen Jonathan D. Constraints associated with cognitive control and the stability-flexibility dilemma. In CogSci. shenhavlab.org, 2018.

[24] Jaffe Paul I, Poldrack Russell A, Schafer Robert J, and Bissett Patrick G. Modelling human behaviour in cognitive tasks with latent dynamical systems. Nature Human Behaviour, pages 1–15, January 2023. 10.1038/s41562-022-01510-8 36658212

[25] Hall-McMaster Sam, Muhle-Karbe Paul S, Myers Nicholas E, and Stokes Mark G. Reward boosts neural coding of task rules to optimize cognitive flexibility. J. Neurosci., 39(43):8549–8561, October 2019. 31519820 10.1523/JNEUROSCI.0631-19.2019 PMC6807286

[26] Cohen J D, Dunbar K, and McClelland J L. On the control of automatic processes: a parallel distributed processing account of the stroop effect. Psychol. Rev., 97(3):332–361, July 1990. 2200075 10.1037/0033-295x.97.3.332

[27] Wallis J D, Anderson K C, and Miller E K. Single neurons in prefrontal cortex encode abstract rules. Nature, 411(6840):953–956, June 2001. 11418860 10.1038/35082081

[28] Woolgar Alexandra, Hampshire Adam, Thompson Russell, and Duncan John. Adaptive coding of task-relevant information in human frontoparietal cortex. J. Neurosci., 31(41):14592–14599, October 2011. 21994375 10.1523/JNEUROSCI.2616-11.2011 PMC6703398

[29] Morey Richard D. Confidence intervals from normalized data: A correction to cousineau. In Tutorials in Quantitative Methods for Psychology, 4. Citeseer, 2008.

[30] Smith Stephen M and Nichols Thomas E. Threshold-free cluster enhancement: addressing problems of smoothing, threshold dependence and localisation in cluster inference. Neuroimage, 44(1):83–98, January 2009. 18501637 10.1016/j.neuroimage.2008.03.061

[31] Mensen Armand and Khatami Ramin. Advanced EEG analysis using threshold-free cluster-enhancement and non-parametric statistics. Neuroimage, 67:111–118, February 2013. 23123297 10.1016/j.neuroimage.2012.10.027

[32] Yeung Nick, Nystrom Leigh E, Aronson Jessica A, and Cohen Jonathan D. Between-task competition and cognitive control in task switching. J. Neurosci., 26(5):1429–1438, February 2006. 16452666 10.1523/JNEUROSCI.3109-05.2006 PMC6675498

[33] Etzel J A, Cole M W, Zacks J M, Kay K N, and Braver T S. Reward motivation enhances task coding in frontoparietal cortex. Cereb. Cortex, 26(4):1647–1659, April 2016. 25601237 10.1093/cercor/bhu327 PMC4785950

[34] Ritz Harrison and Shenhav Amitai. Orthogonal neural encoding of targets and distractors supports multivariate cognitive control. Nature Human Behaviour, pages 1–17, March 2024. 10.1038/s41562-024-01826-7 PMC11219097 38459265

[35] King J-R and Dehaene S. Characterizing the dynamics of mental representations: the temporal generalization method. Trends Cogn. Sci., 18(4):203–210, April 2014. 24593982 10.1016/j.tics.2014.01.002 PMC5635958

[36] Brown E N, Frank L M, Tang D, Quirk M C, and Wilson M A. A statistical paradigm for neural spike train decoding applied to position prediction from ensemble firing patterns of rat hippocampal place cells. J. Neurosci., 18(18):7411–7425, September 1998. 9736661 10.1523/JNEUROSCI.18-18-07411.1998 PMC6793233

[37] Smith Anne C and Brown Emery N. Estimating a state-space model from point process observations. Neural Comput., 15(5):965–991, May 2003. 12803953 10.1162/089976603765202622

[38] Macke Jakob H, Büsing Lars, Cunningham John P, Ece Byron M Yu, Shenoy Krishna V, and Sahani Maneesh. Empirical models of spiking in neural populations. In Advances in neural information processing systems, 2011.

[39] Linderman Scott, Nichols Annika, Blei David, Zimmer Manuel, and Paninski Liam. Hierarchical recurrent state space models reveal discrete and continuous dynamics of neural activity in C. elegans. bioRxiv, page 621540, April 2019.

[40] Hess Florian, Monfared Zahra, Brenner Manuel, and Durstewitz Daniel. Generalized teacher forcing for learning chaotic dynamics. In International Conference on Machine Learning, pages 13017–13049. PMLR, July 2023.

[41] Pals Matthijs, Sağtekin A Erdem, Pei Felix, Gloeckler Manuel, and Macke Jakob H. Inferring stochastic low-rank recurrent neural networks from neural data. arXiv [cs.LG], June 2024.

[42] Lehmann D, Ozaki H, and Pal I. EEG alpha map series: brain micro-states by space-oriented adaptive segmentation. Electroencephalogr. Clin. Neurophysiol., 67(3):271–288, September 1987. 2441961 10.1016/0013-4694(87)90025-3

[43] Gohil Chetan, Kohl Oliver, Huang Rukuang, van Es Mats W J, Jones Oiwi Parker, Hunt Laurence T, Quinn Andrew J, and Woolrich Mark W. Dynamic network analysis of electrophysiological task data. Imaging Neuroscience, 2:1–19, July 2024. 10.1162/imag_a_00226 PMC12272243 40800366

[44] Nozari Erfan, Bertolero Maxwell A, Stiso Jennifer, Caciagli Lorenzo, Cornblath Eli J, He Xiaosong, Mahadevan Arun S, Pappas George J, and Bassett Dani S. Macroscopic resting-state brain dynamics are best described by linear models. Nat. Biomed. Eng., pages 1–17, December 2023. 38082179 10.1038/s41551-023-01117-y PMC11357987

[45] Soldado-Magraner Joana, Mante Valerio, and Sahani Maneesh. Inferring context-dependent computations through linear approximations of prefrontal cortex dynamics. bioRxiv, page 2023.02.06.527389, February 2023. 10.1126/sciadv.adl4743 PMC11654703 39693450

[46] Zhang Yizi, Lyu Hanrui, Hurwitz Cole, Wang Shuqi, Charles Lincoln Findling Felix Hubert, Pouget Alexandre, International Brain Laboratory, Varol Erdem, and Paninski Liam. Exploiting correlations across trials and behavioral sessions to improve neural decoding. bioRxivorg, page 2024.09.14.613047, September 2024. 10.1016/j.neuron.2025.10.026 PMC12695064 41308644

[47] Williams Robert L and Lawrence Douglas A. Linear state-space control systems. John Wiley & Sons, Nashville, TN, January 2007.

[48] Tang E and Bassett D S. Colloquium: Control of dynamics in brain networks. Rev. Mod. Phys., 2018.

[49] Ghahramani Zoubin, Rey Geo, and Hinton E. Parameter estimation for linear dynamical systems. Techinical Report, 1996.

[50] Murphy Kevin P. Probabilistic Machine Learning: Advanced Topics. MIT Press, London, England, 2023.

[51] Churchland Mark M, Yu Byron M, Sahani Maneesh, and Shenoy Krishna V. Techniques for extracting single-trial activity patterns from large-scale neural recordings. Curr. Opin. Neurobiol., 17(5):609–618, October 2007. 18093826 10.1016/j.conb.2007.11.001 PMC2238690

[52] MacDowell Camden J, Briones Brandy A, Lenzi Michael J, Gustison Morgan L, and Buschman Timothy J. Differences in the expression of cortex-wide neural dynamics are related to behavioral phenotype. Curr. Biol., February 2024. 10.1016/j.cub.2024.02.004 PMC10965364 38417445

[53] Stephan Klaas Enno, Penny ill D, Daunizeau Jean, Moran Rosalyn J, and Friston Karl J. Bayesian model selection for group studies. Neuroimage, 46(4):1004–1017, July 2009. 19306932 10.1016/j.neuroimage.2009.03.025 PMC2703732

[54] Brunton Steven L, Budišić Marko, Kaiser Eurika, and Kutz J Nathan. Modern koopman theory for dynamical systems. arXiv [math.DS], February 2021.

[55] Durstewitz Daniel, Koppe Georgia, and Thurm Max Ingo. Reconstructing computational system dynamics from neural data with recurrent neural networks. Nat. Rev. Neurosci., pages 1–18, October 2023. 37794121 10.1038/s41583-023-00740-7

[56] Staffans Olof. Encyclopedia of mathematics and its applications: Well-posed linear systems series number 103. Cambridge University Press, Cambridge, England, October 2009.

[57] Stroud Jake P, Duncan John, and Lengyel Máté. The computational foundations of dynamic coding in working memory. Trends Cogn. Sci., 0(0), April 2024. 10.1016/j.tics.2024.02.011 38580528

[58] Kao Ta-Chu and Hennequin Guillaume. Neuroscience out of control: control-theoretic perspectives on neural circuit dynamics. Curr. Opin. Neurobiol., 58:122–129, September 2019. 31563084 10.1016/j.conb.2019.09.001

[59] Holroyd Clay B. The controllosphere: The neural origin of cognitive effort. Psychol. Rev., February 2024. 10.1037/rev0000467 38358716

[60] Song H Francis, Yang Guangyu R, and Wang Xiao-Jing. Training excitatory-inhibitory recurrent neural networks for cognitive tasks: A simple and flexible framework. PLoS Comput. Biol., 12(2):e1004792, February 2016. 26928718 10.1371/journal.pcbi.1004792 PMC4771709

[61] Gilbert Sam J and Shallice Tim. Task switching: a PDP model. Cogn. Psychol., 44(3):297–337, May 2002. 11971634 10.1006/cogp.2001.0770

[62] Brown Joshua W, Reynolds Jeremy R, and Braver Todd S. A computational model of fractionated conflict-control mechanisms in task-switching. Cogn. Psychol., 55(1):37–85, August 2007. 17078941 10.1016/j.cogpsych.2006.09.005

[63] Herd Seth A, O’Reilly Randall C, Hazy Tom E, Chatham Christopher H, Brant Angela M, and Friedman Naomi P. A neural network model of individual differences in task switching abilities. Neuropsychologia, 62:375–389, September 2014. 24791709 10.1016/j.neuropsychologia.2014.04.014 PMC4167201

[64] Cohen J D, Servan-Schreiber D, and McClelland J L. A parallel distributed processing approach to automaticity. Am. J. Psychol., 105(2):239–269, 1992. 1621882

[65] Siegel Markus, Donner Tobias H, and Engel Andreas K. Spectral fingerprints of large-scale neuronal interactions. Nat. Rev. Neurosci., 13(2):121–134, January 2012. 22233726 10.1038/nrn3137

[66] Mante V, Sussillo D, Shenoy K V, and Newsome W T. Context-dependent computation by recurrent dynamics in prefrontal cortex. Nature, 503(7474):78–84, 2013. 24201281 10.1038/nature12742 PMC4121670

[67] Langdon Christopher and Engel Tatiana A. Latent circuit inference from heterogeneous neural responses during cognitive tasks. bioRxiv, page 2022.01.23.477431, January 2022. 10.1038/s41593-025-01869-7 PMC11893458 39930096

[68] Braver Todd S and Cohen Jonathan D. Chapter 19 dopamine, cognitive control, and schizophrenia: the gating model. In Progress in Brain Research, volume 121 of Progress in brain research, pages 327–349. Elsevier, 1999. 10.1016/s0079-6123(08)63082-4 10551035

[69] O’Reilly Randall C and Frank Michael J. Making working memory work: a computational model of learning in the prefrontal cortex and basal ganglia. Neural Comput., 18(2):283–328, February 2006. 16378516 10.1162/089976606775093909

[70] Allport D A, Styles E A, Hsieh Shulan, Umilta Carlo, and Moscovitch Morris. Attention and performance XV: Conscious and nonconscious information processing, 1994.

[71] Friston K J, Harrison L, and Penny W. Dynamic causal modelling. Neuroimage, 19(4):1273–1302, August 2003. 12948688 10.1016/s1053-8119(03)00202-7

[72] Galka Andreas, Yamashita Okito, Ozaki Tohru, Biscay Rolando, and Valdés-Sosa Pedro. A solution to the dynamical inverse problem of EEG generation using spatiotemporal kalman filtering. Neuroimage, 23(2):435–453, October 2004. 15488394 10.1016/j.neuroimage.2004.02.022

[73] Pirondini Elvira, Babadi Behtash, Obregon-Henao Gabriel, Lamus Camilo, Malik Wasim Q, Hamalainen Matti S, and Purdon Patrick L. Computationally efficient algorithms for sparse, dynamic solutions to the EEG source localization problem. IEEE Trans. Biomed. Eng., 65 (6):1359–1372, June 2018. 28920892 10.1109/TBME.2017.2739824

[74] Goldman-Rakic P S. Topography of cognition: parallel distributed networks in primate association cortex. Annu. Rev. Neurosci., 11:137–156, 1988. 3284439 10.1146/annurev.ne.11.030188.001033

[75] Miller E K and Cohen J D. An integrative theory of prefrontal cortex function. Annu. Rev. Neurosci., 24:167–202, 2001. 11283309 10.1146/annurev.neuro.24.1.167

[76] Walther Alexander, Nili Hamed, Ejaz Naveed, Alink Arjen, Kriegeskorte Nikolaus, and Diedrichsen Jörn. Reliability of dissimilarity measures for multi-voxel pattern analysis. Neuroimage, 137:188–200, August 2016. 26707889 10.1016/j.neuroimage.2015.12.012

[77] e Holmes Elizabeth, j Ward Eric, and Wills Kellie. MARSS: Multivariate autoregressive state-space models for analyzing time-series data. R J., 4(1):11, 2012.

[78] Smith Gavin, de Freitas João, Robinson Tony, and Niranjan Mahesan. Speech modelling using subspace and EM techniques. Advances in Neural Information Processing Systems, 12, 1999.

[79] Stone Iris R, Sagiv Yotam, Park Il Memming, and Pillow Jonathan W. Spectral learning of bernoulli linear dynamical systems models. arXiv [stat.ML], March 2023. PMC12981602 41836604

[80] Larimore W E. Canonical variate analysis in identification, filtering, and adaptive control. In 29th IEEE Conference on Decision and Control, pages 596–604 vol.2. IEEE, 1990.

[81] Ehinger Benedikt V and Dimigen Olaf. Unfold: an integrated toolbox for overlap correction, non-linear modeling, and regression-based EEG analysis. PeerJ, 7(e7838):e7838, October 2019. 31660265 10.7717/peerj.7838 PMC6815663

[82] Cox D R and Wermuth Nanny. A comment on the coefficient of determination for binary responses. Am. Stat., 46(1):1–4, February 1992.

[83] Paszke Adam, Gross Sam, Massa Francisco, Lerer Adam, Bradbury James, Chanan Gregory, Killeen Trevor, Lin Zeming, Gimelshein Natalia, Antiga Luca, Desmaison Alban, Köpf Andreas, Yang Edward, DeVito Zach, Raison Martin, Tejani Alykhan, Chilamkurthy Sasank, Steiner Benoit, Fang Lu, Bai Junjie, and Chintala Soumith. PyTorch: An imperative style, high-performance deep learning library. arXiv [cs.LG], December 2019.

[84] Hinton G E and Salakhutdinov R R. Reducing the dimensionality of data with neural networks. Science, 313(5786):504–507, July 2006. 16873662 10.1126/science.1127647

[85] Loshchilov Ilya and Hutter Frank. Decoupled weight decay regularization. November 2017.

[86] Kalman R E. Lectures on controllability and observability. In Controllability and Observability, pages 1–149. Springer Berlin Heidelberg, Berlin, Heidelberg, 2010.

[87] Ehrlich Daniel B and Murray John D. Geometry of neural computation unifies working memory and planning. Proc. Natl. Acad. Sci. U. S. A., 119(37):e2115610119, September 2022. 36067286 10.1073/pnas.2115610119 PMC9478653

[88] Cho Kyunghyun, van Merrienboer Bart, Gulcehre Caglar, Bahdanau Dzmitry, Bougares Fethi, Schwenk Holger, and Bengio Yoshua. Learning phrase representations using RNN encoder-decoder for statistical machine translation. arXiv [cs.CL], June 2014.

[89] Oostenveld Robert, Fries Pascal, Maris Eric, and Schoffelen Jan-Mathijs. FieldTrip: Open source software for advanced analysis of MEG, EEG, and invasive electrophysiological data. Comput. Intell. Neurosci., 2011(1):156869, January 2011. 21253357 10.1155/2011/156869 PMC3021840

[90] Delorme Arnaud and Makeig Scott. EEGLAB: an open source toolbox for analysis of single-trial EEG dynamics including independent component analysis. J. Neurosci. Methods, 134 (1):9–21, March 2004. 15102499 10.1016/j.jneumeth.2003.10.009

[91] Crameri Fabio, Shephard Grace E, and Heron Philip J. The misuse of colour in science communication. Nat. Commun., 11(1):5444, October 2020. 33116149 10.1038/s41467-020-19160-7 PMC7595127

