# Humans actively reconfigure neural task states

**DOI:** 10.1101/2024.09.29.615736

**Published:** 2025-02-28

**Authors:** Harrison Ritz, Aditi Jha, Nathaniel D. Daw, Jonathan D. Cohen

**Affiliations:** 1 Princeton Neuroscience Institute, Princeton University; 2 Department of Statistics, Stanford University; 3 Department of Psychology, Princeton University

**Keywords:** task switching, electroencephalography, state-space model, recurrent neural networks

## Abstract

The ability to switch between tasks is a core component of adaptive cognition, yet a mechanistic understanding of this capacity has remained elusive. In particular, there are longstanding debates over the extent to which task-switching is primarily influenced by interference from previous tasks or active preparation for upcoming tasks. We advance this debate by modeling the evolution of task representations in human electroencephalographic recordings as linear dynamical systems over a high-dimensional latent space. Using control theoretic analyses of dynamical systems fit to both brains and task-optimized neural networks, we reveal neural signatures of active preparation that reconcile the role of interference and control in task switching. These findings inform a core debate in cognitive control and offer a promising new paradigm for human neuroimaging analysis.

Humans have a remarkable capacity to flexibly adapt how they perform tasks (1–4). This flexibility reflects a core feature of goal-directed cognition (5) and is a strong indicator of cognitive changes across the lifespan (6, 7) and in mental health (8, 9). Despite the centrality of cognitive flexibility in our mental life, we still have a limited mechanistic understanding of how people rapidly configure task processing to achieve their moment-to-moment goals.

Previous research has investigated task preparation primarily through the lens of performance costs associated with rapidly switching stimulus-response mappings (‘task sets’; (2)). There is broad agreement that these switch costs depend both on the interference of the previous trial and preparation for the upcoming trial (2, 3). However, there are long-standing debates about the relative contributions of these two processes. The ‘task set inertia’ hypothesis argues that switch costs largely reflect interference from the previous trial, which passively decays over time (10, 11). The ‘active reconfiguration’ hypothesis argues that switch costs largely reflect active preparation for the upcoming task through cognitive control (1, 12). Previous experiments have attempted to adjudicate between these hypotheses using behavioral analyses or time-locked neuroimaging (13–18), but have faced challenges in adequately characterizing the underlying dynamics of task preparation.

In parallel, recent research in the neuroscience of *motor* control has explored how the brain prepares for upcoming motor actions. A major advance in this field has been the application of dynamical systems theory to explain how neural populations evolve toward action-specific brain states (19, 20). This framework offers a promising approach for studying the dynamics of *cognitive* control, aligning with recent simulations that use dynamical systems theory to model transitions between neurocognitive task states (21–23). Despite the opportunity for dynamical approaches to help adjudicate between classic theories, this approach remains underutilized for characterizing the neural dynamics of task preparation.

To bridge between dynamics-based theories and neural mechanisms, we fit large-scale dynamical systems to two human electroencephalographic (EEG) datasets, tracking the evolution of neural task states during preparation. Using control-theoretic analysis of these fitted dynamical systems, we tested the predictions of the Inertia and Reconfiguration hypotheses in both neural recordings and task-optimized recurrent neural networks (RNNs). We find that common dynamical motifs across biological and artificial neural systems support the active reconfiguration of neural task states, while reconciling these competing accounts.

## Neural measures of task preparation

**Fig. 1.**
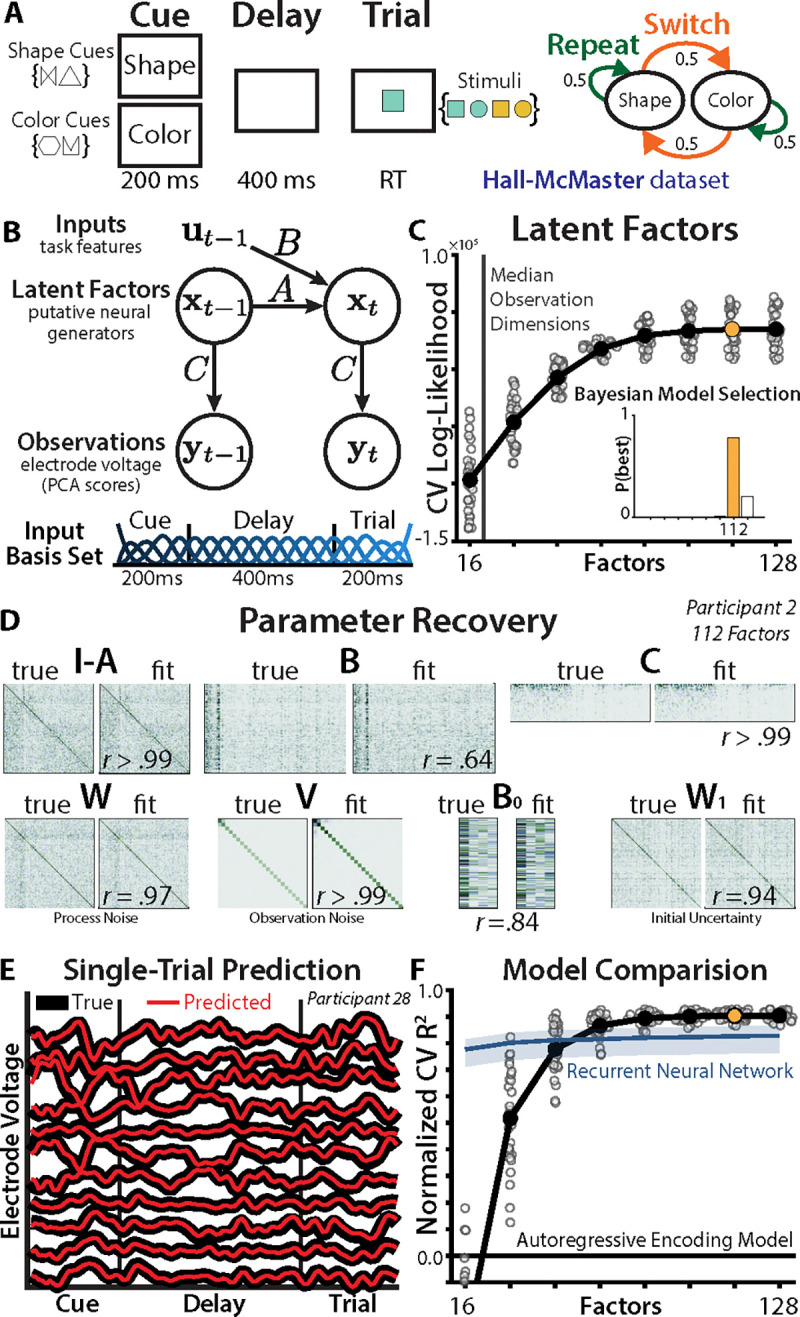
State space analysis. **(A)** Left: Participants saw a task cue (two symbols mapped each task), waited through a delay period, then responded based on the cued dimension. Right: The tasks had an equal change of repeating or switching between trials. **(B)** Top: Schematic of the state space model. ‘PCA’: principal components analysis. Bottom: temporal spline basis set, spanning the epoch. **(C)** The log-likelihood from a held-out test set (y-axis), across different number of factors (x-axis). Dots indicate single subjects; log-likelihood is mean-centered within-participant to show relative differences. Inset: Bayesian model selection. **(D)** Parameter recovery on a synthetic dataset generated from a participants’ estimated parameters. **(E)** Predictive accuracy in one example trial from the test set across a subset of the EEG electrodes. Thick black lines indicate EEG voltage, red lines indicate next-timestep predictions from a Kalman filter (112-factor model). **(F)** Cross-validated coefficient of determination for SSMs across factor size, relative to autoregressive encoding models (zero indicates equally-good fit). Blue line and shading indicates the 95% interval of the predictive accuracy of parameter-matched RNNs fit to each participant. Y-axis truncated for visualization.

We first analyzed scalp EEG recordings made in a recent experiment by Hall-McMaster et al. (24), in which 30 participants performed a cued task-switching paradigm (Fig. 1A; Method 1 and 2). On each trial, participants were cued whether to perform the ‘shape’ or ‘color’ task. After a delay period, participants saw a colored shape and responded with a button press based on the cued dimension. Participants had poorer performance when they had to switch tasks, and the authors’ EEG analyses revealed sensor-level encoding of task identity during the delay period. This task encoding was interpreted as a preparatory brain state that facilitated task-appropriate behavior (24–27).

It has previously been proposed that the strength of task encoding on switch versus repeat trials can adjudicate between task-switching theories (15, 18). However, the sensor-level analyses of these EEG data did not show significant differences in encoding strength (Fig. S1; Method 3). However, sensor-level encoding strength may be an insufficient test of theories that make specific predictions about the *dynamics* of preparatory task representations, which are covert and reflected only indirectly in measured signals. The Inertia hypothesis predicts the passive decay of the previous task representation, whereas the Reconfiguration hypothesis predicts strengthening of representations for the upcoming task. To test these predictions, we turned to a modeling approach that can characterize the rich temporal structure of the underlying neural system.

## Inferring neural state-space dynamics

We quantified the evolution of preparatory task representations using a linear state space model (SSM). Unlike traditional neuroimaging analyses, SSMs account for the temporal structure of neural representations, here modeled as a linear dynamical system over a set of latent factors. Linear dynamical systems have long been used in computational neuroscience to model latent dynamics in multiunit neural recordings (28–30). Modeling activity at the level of latent factors can capture rich (non-Markovian) dynamics (31), reflecting the neural sources of the recorded electrode voltage. Despite the nonlinearity of neural activation functions (32), linear models often provide highly predictive models of neural dynamics (33–35). Critically, the linear SSM can be characterized using a suite of tools from dynamical systems theory and control theory (36, 37), providing an interpretable alternative to nonlinear SSMs, such as hidden Markov models (38–40) or RNNs (41, 42).

The SSM consisted of a set of latent factors (state vector x; Fig. 1B; Method 5) that evolved according to an autoregressive process described by a recurrence matrix (A), inputs (with input vector u and input matrix B), and Gaussian ‘process noise’ $\left(w_{t}\;\sim \;𝒩(0,W)\right)$:

(1) $$\;x_{t}=Ax_{t-1}+Bu_{t-1}+w_{t}\;x_{t=1}=B_{0}u_{0}+w_{1}$$

We captured task switching dynamics by including both the previous and current task identity as inputs (see Method 6 for complete predictor list). To provide additional flexibility to the model, we expanded the inputs with a temporal spline basis (bottom of Fig. 1B; Method 6). To better capture task set inertia, the previous task identity ($B_{0}^{\text{prevTask}}u_{0}^{\text{prevTask}}$) was included in the regressors for the initial condition (see Method 6).

To predict the EEG observations (y; see below), factors were ‘read out’ through the observation matrix (C) and corrupted by ‘observation noise’ $\left(v_{t}\;\sim 𝒩(0,V)\right)$:

(2) $$y_{t}=Cx_{t}+v_{t}$$

We reduced the dimensionality of the EEG recordings using principal component analysis (PCA), retaining components that accounted for 99% of the variance (between 14–27). We fit the model to an epoch that included the cue period, delay period, and the beginning of the trial (before any response). We estimated the maximum a posteriori parameters of the SSM using expectation-maximization (Method 3, (43, 44)), developing an open-source package called StateSpaceAnalysis.jl (45).

## EEG recordings are well-explained by a high-dimensional SSM

We first assessed the dimensionality of the latent space, choosing the number of factors based on cross-validated prediction. In systems neuroscience, latent variable models of multiunit neural recordings typically have fewer factors than observation dimensions (46, 47). Surprisingly, we found that an SSM with many more factors than the original number of EEG electrodes provided the best cross-validated fit (best model: 112 factors with 90% protected exceedance probability; Fig. 1C; (48)). This ‘lifting’ of the dimensionality may allow a linear model to better approximate the underlying nonlinear system (49).

To validate the reliability of this high-dimensional model, we evaluated the SSM on a synthetic dataset based on a participant’s parameters (Fig. 1D; after factor alignment, see Method 6). We could accurately recover the ground-truth parameters, supporting the precision of the fitting procedure and the ability to differentiate effects between parameters.

The best-fitting model was highly accurate at predicting next-timestep EEG activity in held-out data (median $R_{CV}^{2}=.98$), even at the level of single trials (Fig. 1E). The model also made accurate predictions at longer horizons (median t+200ms accuracy: $R_{CV}^{2}\;=.43$; Fig. S2A), and open-loop simulations closely reproduced the empirical power spectra (median $R_{CV}^{2}=.98$; Fig. S2B; (50)). Finally, model comparisons revealed that the best-fitting SSM performed better than both simpler autoregressive encoding models (i.e., without latent factors) and nonlinear recurrent neural networks (matched for parameters; Fig. 1F; Method 7). This suggests that these SSMs strike a good balance between the interpretability of linear models and the expressivity of nonlinear models.

## Latent neural dynamics reveal stable task control

Having validated the predictive power of the SSM, we explored whether the brain transitions into task-specific neural attractors (points of local convergence) during preparation. While the Inertia and Reconfiguration hypotheses are agnostic to this property, task attractors are central to dynamic theories of task switching (21, 22), and to our knowledge remain untested.

To assess the evolution of neural task states towards an attractor, we factorized the SSM into separate subsystems for each task, a procedure known as ‘additive state decomposition’ ((51); Method 8):

(3) $$x_{t}^{\text{task}}=Ax_{t-1}^{\text{task}}+B^{\text{task}}u_{t-1}^{\text{task}}\;x_{t=1}^{\text{task}}=B_{0}^{\text{prevTask}}u_{0}^{\text{prevTask}}$$

**Fig. 2.**
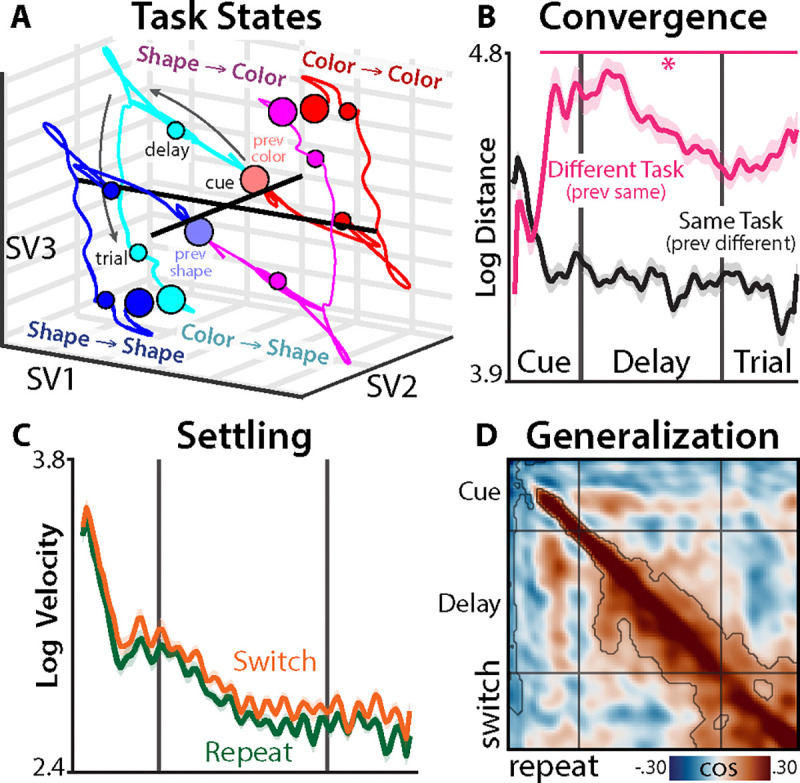
Neural task attractors. **(A)** Simulated task states for each task, split by switch and repeat conditions. Task is contrast-coded, so tasks within the same switch condition are symmetrical. ‘SV’: singular vector. **(B)** Log Euclidean distances across switch and repeat conditions for the same task (black; e.g., cyan vs. blue in (A)) and different tasks (pink; e.g., cyan vs. red in (A)). **(C)** Log task state velocity, plotted for switch trials (orange) and repeat trials (green). First and last timepoint are excluded to accommodate edge effects in the temporal basis set. **(D)** Cosine similarity between task states across switch and repeat trials, at different temporal lags. Grid lines indicate the cue, delay, and trial periods. Asterisks and contours indicate p<.05 (TFCE-corrected; (52)).

When we visualized participants’ task trajectories using singular value decomposition (SVD; Method 8), we observed distinct trajectories for each task that appeared relatively consistent across switch and repeat trials (Fig. 2A). Thus, while the fitted systems were stable (resulting in a global attractor), time-varying inputs could produce complex trajectories.

We first verified that task states converged to similar locations from different initial conditions, measuring the Euclidean distances between states across switch and repeat conditions (Method 8). Brain states for the same task (e.g., red-magenta in Fig. 2A) became more similar over time (group-level t-test on distance-time correlations: d=−1.1, p<.001; Fig. 2B, black). By contrast, states for different tasks (e.g., red-cyan in Fig. 2A) diverged during the cue period and then maintained separation (Fig. 2B, pink). Both of these dynamics support convergence to a task-specific attractor state.

We next assessed whether task states decelerated as they settled into their putative attractors. Consistent with settling, task state velocity slowed over time (group-level t-test on velocity-time correlations: ds<−5.0, ps<.001 across switch and repeat trials). Moreover, task states decelerated more as they approached states that were reached after different initial conditions (i.e., the putative attractor location; group-level t-test on velocity-distance correlations: d=0.97, p<.001, controlling for linear and quadratic effects of time).

We also tested the prediction that neural states should become more stable as they approach an attractor (53). We quantified state stability by computing the cosine similarity between task states at different temporal lags (54). Consistent with stabilization, we found that generalization grew over the epoch (broadening similarity in Fig. 2C), with significant similarity growth for lags up to 224 ms (Fig. S3).

Finally, we confirmed that these neural task states were behaviorally relevant, finding that participants had faster reaction times on trials in which their EEG had higher cosine similarity with the sensor-projected task state (summed over timesteps: d=−0.55, p=.0054; Fig. S4; Method 8). Together, these findings support key tenets of dynamic theories of task-switching, shared by both the Inertia and Reconfiguration hypotheses: the brain transitions into stable, task-specific states that promote good performance.

## Task dynamics support Reconfiguration

We next examined whether the fitted models could discriminate between the Inertia and Reconfiguration hypotheses. These hypotheses make opposing predictions for how strongly cued task representations should propagate through the brain on switch trials relative to repeat trials. Inertia predicts weaker task propagation on switch trials, due to interference from the previous task. Reconfiguration predicts stronger task propagation on switch trials, due to the deployment of cognitive control.

To model the propagation of task representations, we used ‘Lyapunov functions,’ standard tools from control theory for analyzing linear dynamical systems (36). Lyapunov functions have been used in neuroscience for asymptotic ‘controllability’ analyses of how input energy will propagate through a neural system (37, 55, 56). Here, we quantified the time-resolved energy of task inputs, using the recursive form of the Lyapunov equation (57):

(4) $$X_{t}^{\text{task}}=AX_{t-1}^{\text{task}}A^{⊤}+(B^{\text{task}}u_{t-1}^{\text{task}}){\left(B^{\text{task}}u_{t-1}^{\text{task}}\right)}^{⊤}\;X_{t=1}^{\text{task}}=(B_{0}^{\text{prevTask}}u_{0}^{\text{prevTask}}){\left(B_{0}^{\text{prevTask}}u_{0}^{\text{prevTask}}\right)}^{⊤}$$

**Fig. 3.**
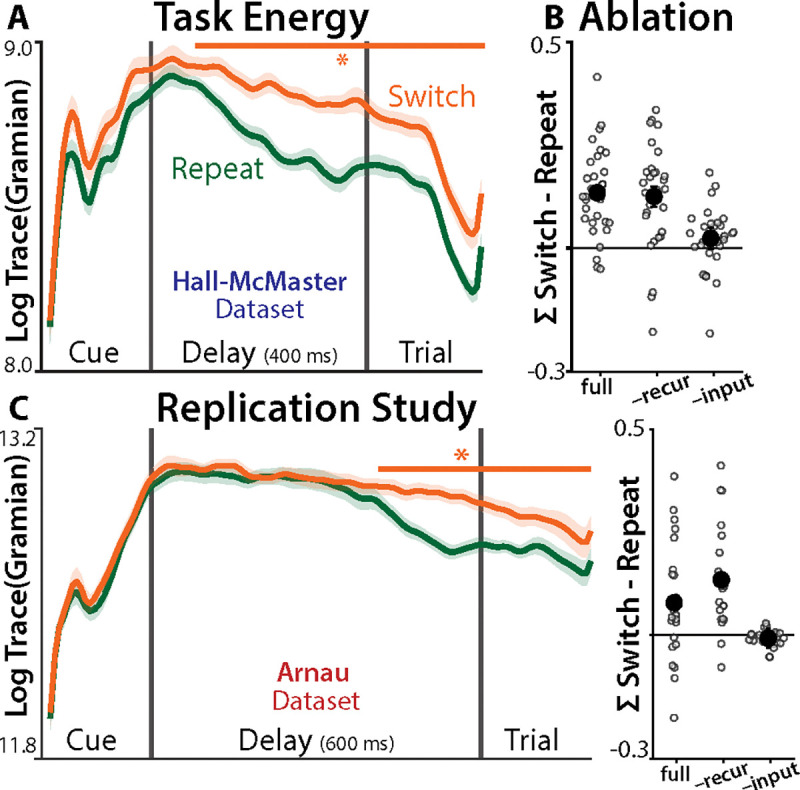
Task Energy. **(A)** The log trace of the Task Gramian, separately for switch (orange) and repeat (green) conditions. Error bars indicate within-participant SEM; asterisks indicate p<.05 (TFCE). **(B)** Time-averaged contrast between task Gramians across switch and repeat conditions, computed under different model ablations. ‘full’: full model. ‘–recur’: setting A and $B_{0}$ to zero. ‘–inputs’: z-scoring $B^{\text{task}}u_{t}^{\text{task}}$ at each timestep. Dots indicate participants, error bars indicate within-participant SEM. **(C)** Replication of (A) and (B) in an independent dataset.

Consistent with the Reconfiguration hypothesis, we found that the total task energy $\left(\sum_{t=1}^{T}log\;trace(X_{t}^{\text{task}})\right)$ was stronger when participants switched tasks than when they repeated tasks (Fig. 3A; group-level t-test: =1.2, $p=1.9\times 10^{-7}$).

While these differences in task energy are consistent with the Reconfiguration hypothesis, it is still possible that they emerge from the recurrent dynamics that are central to the Inertia account. To test this, we estimated task energy after ablating both the fitted recurrence (A) and the initial conditions $\left(B_{0}^{\text{prevTask}}\right)$, components which together captured the passive decay of the previous task set. This ablation did not reduce the difference in task energy between switch conditions (Fig. 3B ‘-recur’; Fig. S5). To directly test the Reconfiguration hypothesis, we ablated time-varying task inputs $\left(B^{\text{task}}\right)$. Here, we found that standardizing inputs at each timestep strongly reduced differences in task energy between switch conditions (Fig. 3B ‘-input’), further supporting the Reconfiguration account.

We confirmed the generalizability of these findings in an independent EEG dataset from Arnau et al. ((58); N=26, Method 1), which used shorter intertrial intervals (ITIs: 800 ms vs. 2200 ms; Method 2). Comparing short and long ITIs provides another way of testing for task set inertia: inertia should depend on the time from the previous task, unlike reconfiguration, which has been thought to depend only on the time since the cue (13). However, analysis of the Arnau dataset replicated the support for Reconfiguration. We found stronger, input-driven, task energy on switch trials (Fig. 3C; d=0.68, p=.0068; between-study: d=0.37, p=.17; ${BF}_{\text{null}}=1.69$), and a replication of the other key results (Fig. S6).

These task state analyses provided consistent support for theories of neural task attractors in general, and the Reconfiguration hypothesis in particular. Time-varying inputs appear to move the brain into a stable task state, especially when switching between tasks, which we hypothesized reflects an underlying cognitive control process (22, 25).

## Task switching in recurrent neural networks

To facilitate a stronger interpretation of the potential role for reconfiguration in task switching, we formalized the Inertia and Reconfiguration hypotheses using gated RNNs. These models provide full access to task representations in nonlinear dynamical systems trained to perform cognitive tasks.

While previous work has developed neural network models of inertia and reconfiguration, these have been limited by using hand-crafted structural mechanisms (21, 59–61), and have not adjudicated between these theories on the basis of their neural predictions. We addressed this by modeling task-switching hypotheses using different training curricula (22, 62), and then directly compared the networks’ task-dependent dynamics to those observed in human EEG.

**Fig. 4.**
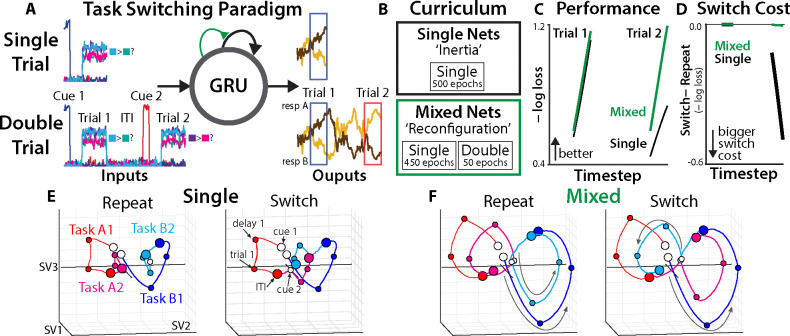
Task dynamics in neural networks. **(A)** example of a ‘single trial’ (top) and a ‘double trial’ (bottom). **(B)** Single networks were only trained on single trials, whereas mixed networks had their final 10% of training on double trials. **(C)** Time-resolved loss over the course of double trial sequences, estimated after training. Mixed networks in green, single networks in black. Averaged over 512 networks; error bars are smaller than line width. **(D)** Difference in time-resolved loss for switch and repeat trials. **(E-F)** Hidden unit activity during double trials, projected to three dimensions with SVD. White dots are the initial timestep of each trial, colored dots are the final timestep of each trial. Shown here for the ‘long-ITI’ model (see Fig. 5, Fig. S11).

We trained RNNs on a ‘context-dependent decision-making’ task paralleling the EEG experiments (Method 9). Networks were trained on two kinds of trials. During ‘Single Trials’ they received a task cue, and then were presented with the task-relevant stimulus features after a delay period (Fig. 4A, upper; (63?, 64)). Whereas Single Trials had only one trial per sequence, in ‘Double Trials’ the networks performed two consecutive trials per sequence (Fig. 4A, lower), with an equal probability of switching and repeating tasks.

We used the amount of training on the Single and Double Trials to model the two task-switching hypotheses (Fig. 4B; Method 9). The Inertia hypothesis was captured by ‘Single Networks,’ which had 100% of their training on Single Trials, and were then tested on switch trials. We hypothesized that these networks would show task set inertia in the absence of learning how to actively switch between tasks. Reconfiguration was captured by ‘Mixed Networks,’ which were trained exclusively on Single Trials for the first 90% of training, followed by Double Trials during the last 10% of training. We hypothesized that this explicit experience with switching during training would encourage the learning of active reconfiguration strategies.

## Switching training produces flexible reconfiguration strategies

We first tested whether Single and Mixed Networks offer face-valid simulations of inertia and reconfiguration, training 512 RNNs with gated recurrent units (GRUs) under each curriculum (Method 9). To evaluate the networks’ performance on Double Trials, we plotted the average loss at each timestep. We found that both networks had similar performance on the first trial of each pair, suggesting a similar capacity for task processing. (Fig. 4C). However, Mixed Networks showed much stronger performance on the second trial of each sequence. Mixed Networks’ advantage on second trials was due in part to better switching than Single Networks, with a smaller difference in their loss between switch and repeat trials (Fig. 4D). This is consistent with Mixed Networks learning an adaptive reconfiguration strategy.

We assessed whether Mixed Networks’ switching strategies were generalizable, as one would expect of flexible cognitive control. First, we tested how networks generalized to new trial sequences. Mixed Networks still performed better than Single Networks when tested on three trial sequences (Fig. S7), which neither had experienced during training. Next, we tested how networks generalized to new tasks. We trained another cohort of Mixed and Single networks on four tasks, with Mixed Networks learning to switch between only two of the four tasks (Method 9). After training, Mixed Networks had better switch-trial performance than Single Networks on the held-out task transitions (Fig. S8), consistent with learning a generalizable reconfiguration strategy.

We directly tested that the gating mechanisms were used for active reconfiguration, consistent with prior theories of cognitive control (65, 66). We found that ablating GRU ‘update’ gates during training substantially impaired their ability to learn from Double Trials (Fig. S9), consistent with a role for gating in their active reconfiguration.

Finally, we visualized Single and Mixed RNNs’ hidden-unit activations using SVD (Fig. 4E–F; compare to Fig. 2A; Method 9). Whereas both networks exhibited similar first-trial dynamics, their second-trial dynamics sharply diverged. Single Networks appeared to move (unsuccessfully) between the two end states, whereas Mixed Networks exhibited robust preparatory dynamics that appeared to reproduce the first-trial trajectory.

## Quantifying RNN reconfiguration using state space models

To compare the processing dynamics in Single and Mixed Networks against the human EEG data, we fit state-space models to RNNs’ hidden unit activation during preparation for the second trial in each sequence (Method 10). As in the EEG analyses, SSMs with more latent factors than observation dimensions had the best cross-validation accuracy (next-step $R_{CV}^{2}=\;.90$ for 112 factors; Fig. S10), showcasing the ability of high-dimensional linear models to capture nonlinear systems.

**Fig. 5.**
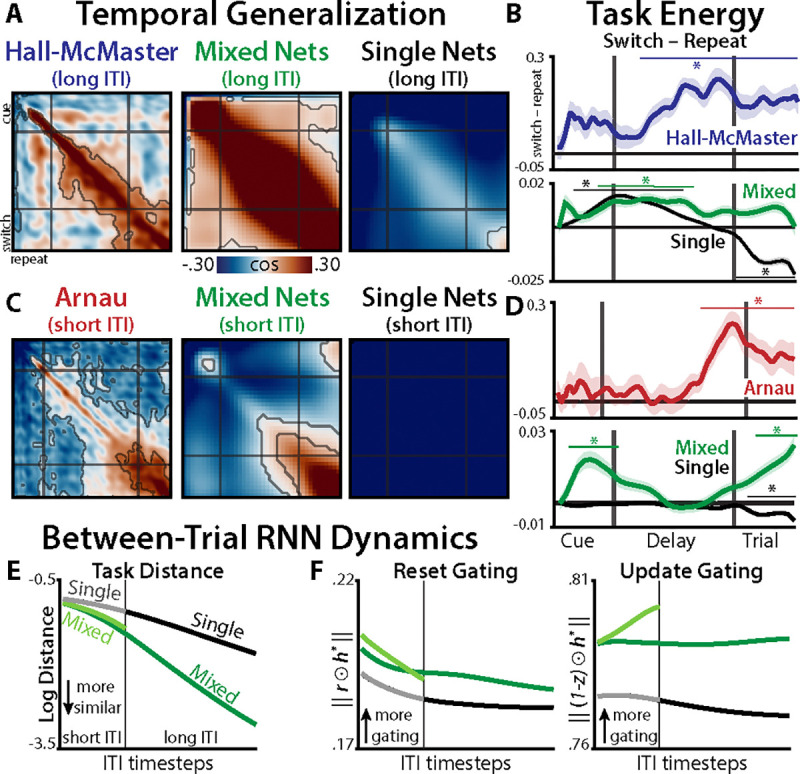
RNN-EEG comparision. **(A)** Temporal generalization of task states in the first EEG dataset (‘Hall-McMaster’) and in RNN models with long intertrial intervals (ITIs). **(B)** Task energy contrasted between switch and repeat trials, plotted for the first EEG dataset (top) and long-ITI RNN models (bottom). **(C)** Temporal generalization and **(D)** task energy in the second EEG dataset (‘Arnau’) and in short-ITI RNNs; compare to (A) and (B). X-axis are not to scale (Hall-McMaster: 100 steps; Arnau: 125 steps; RNNs: 40 steps). **(E)** Euclidian distance between hidden units after each task. **(F)** Alignment between GRU gates (r,z) and a convergence target (e.g., following task A, $h_{t}^{*}={\overset{ˆ}{h}}_{t+1}^{\text{taskB}}$; see Method 9). Contours and asterisks indicates p<.05 (TFCE).

Next, we compared the temporal generalization of task states between RNNs and EEG (Fig. 5A; Method 11). Consistent with the Reconfiguration hypothesis, we found that Mixed Networks had a strikingly similar generalization pattern to human EEG (cosine = [.52, .83], bootstrap 95% confidence interval; Method 11). Both Mixed Networks and EEG had robust generalization of task encoding across switch and repeat conditions. This was in stark contrast to Single Networks, which had poor correspondence to EEG (cosine = [−.39, .024]) and negative alignment of task states across switch and repeat conditions.

We then tested whether RNNs reproduced the EEG signature of stronger task energy in switch trials than repeat trials (see: Fig. 3). As with generalization, we found divergent patterns between Single and Mixed Networks (Fig. 5B). Mixed networks, similar to EEG, had stronger task energy on switch trials (d=0.15, p=.00081; EEG cosine = [.46, .92]), again supporting Reconfiguration. Single Networks, unlike EEG, did not show stronger task energy on switch trials and had poor alignment with EEG (summed energy: d=0.037, p=.41; EEG cosine = [−.40, .21]).

The temporal generalization profile in the Arnau dataset differed from the HallMcMaster dataset (Fig. 5C, left), with negative alignment between early and late task states. This may indicate that there is greater task set inertia in the Arnau dataset, potentially due to shorter ITIs. However, these differences may instead be due to similar reconfiguration mechanisms operating over different ITIs. To test these alternatives, we trained Mixed Networks with shortened ITIs (Method 9). Short-ITI networks closely matched the Arnau dataset on temporal generalization (EEG cosine = [.81, .95]; Fig. 5C) and switch-elevated task energy (d=0.15, p=.001; EEG cosine = [.20, .84]; Fig. 5D). This suggests that participants have processes during the ITI that are similar in Mixed Networks, but missing from classic theories of cue-based reconfiguration.

We explored whether Mixed Networks’ processing during the ITI was dominated by control or inertia by comparing their hidden-unit activity to Single Networks. We found that Mixed Networks more rapidly transitioned into task-neutral states after each trial (Fig. 5E; Fig. S11), which they achieved through elevated control (gating-in Task B states in the ITI after task A; Fig. 5F; Method 9). In sum, Mixed Networks actively configure task-neutral states during the ITI, offering a new explanation of ITI-dependent task preparation centered on control.

## Discussion

Psychologists have debated for decades about the latent processes underlying cognitive flexibility (1, 3, 11). To advance this debate, we quantified people’s latent task processing through high-dimensional dynamical systems fit to two EEG datasets. We found evidence for task-specific neural attractors, a core prediction of dynamic switching theories. Comparing switch and repeat trials, we found stronger task energy when participants switched tasks, consistent with theories of active reconfiguration. Supporting this interpretation, neural networks that were trained to switch between tasks reproduced these neural signatures of reconfiguration. These analyses uncovered new evidence of the reconfiguration of neural task states, while also refining classic theories of task switching.

Task-optimized networks offered a new explanation for how task switching is influenced by ITIs, a major difference between the two EEG studies. During the ITI, networks gated-in a neutral task state (Fig. 5), like a tennis player returning to the center of the court after a volley. Short ITIs didn’t provide networks enough time to reach this neutral state. These simulations offer both a new formalization and a potential reconciliation of task-switching theories. As in the inertia hypothesis, inter-trial configuration will depend on task similarity (67). As in the reconfiguration hypothesis, these dynamics are produced through control. While we found a more expansive role for control than has been assumed in classic theories, including on repeat trials, this is consistent with the ubiquitous dynamics observed during within-task attentional control (68).

One limitation of the current work is that we estimated time-varying inputs to the SSM, including after the cue presentation. These estimated task inputs may reflect an underlying control process, consistent with the analogous dynamics we found in gated RNNs. Optimal feedback control, the gold-standard model of motor control (69, 70), offers a natural process model for control over dynamic neural systems. Notably, preparing for actions (71), for known tasks (72), and, here, for unknown tasks all appear to involve configuring the optimal initial conditions for dynamic processing. Optimal control theory may offer a prime candidate for unifying accounts of this goal-directed control over the neural state space (55, 73, 74).

For cognitive neuroscience more generally, there is high demand for better source reconstruction from MEG and EEG recordings. SSMs provide a powerful method for localizing latent dynamics (75–77), offering Bayesian estimates of source activity from lagged measurements. SSMs can also allow researchers to quantify the spread of information through effective networks (78, 79), offering a clearer view of the distributed neural systems that enable flexible cognition (80, 81).

## Methods

### 1. EEG Sample

#### A. Hall-McMaster Dataset.

The first EEG dataset was originally published by Hall-McMaster and colleagues (24), and was made open-access at https://osf.io/kuzye/. Thirty participants (18–35 years old; 19 females), with normal or corrected-to-normal vision and no history of neurological or psychological disorders, underwent an EEG recording session during task performance. This study was approved by the Central University Research Ethics Committee at the University of Oxford, with all participants providing informed consent. See (24) for the full description.

#### B. Arnau Dataset.

The second EEG dataset was originally published by Arnau and colleagues (58), and was made open-access at https://osf.io/ndgst/. This dataset consists of twenty-six participants (23 female, mean(SD) age 21.65(2.15) years, with normal or corrected-to-normal vision and no history of neurological or psychological disorders, and verified normal color vision. This study was The study was approved by the ethics committee of the Leibniz Research Centre for Working Environment and Human Factors, Dortmund, with all participants providing informed consent. See (58) for the full description.

### 2. EEG Task

#### A. Hall-McMaster dataset.

Participants performed a cued task-switching experiment during the EEG recording. The major goal of the original study was to investigate the role of incentives on task encoding. This study focused on more traditional switching because this directly relates to the core theoretical questions about task set inertia and active reconfiguration.

In the original experiment, the full trial structure involved a cue for high vs low rewards for correct performance (800 ms, 400 ms delay), a cue for the shape vs color task (200 ms, 400 ms delay), a trial stimulus consisting of a square or circle colored in yellow or blue (min(RT, 1400 ms)), reward feedback (200 ms), and an inter-trial interval (1000–1400 ms). Participants’ task was to respond to the trial stimulus according to the cued rule: if the task was ‘shape’, respond with a key press based on the shape; if the task was ‘color’, respond with a key press based on the color. Participants practiced the task until they reached a 70% accuracy criterion, and then performed 10 blocks of 65 trials in the main task. Conditions were balanced within each block. For the analyses, we included trials 1) that were not the first trial of a block, 2) where the current and the previous trial was accurate, 3) where the current RT was longer than 200 ms, and 4) that were not identified as containing artifacts during preprocessing. After preprocessing, we split up the experimental blocks into training and test sets, with Mean(SD) = 416(63) training trials and 47(9) testing trials. EEG data were recorded with 61 Ag/AgCl sintered electrodes (EasyCap; 10–10 layout (Fieldtrip template: elec1010)), a NeuroScan SynAmps RT amplifier, and Curry 7 acquisition software. See (24) for full description.

#### B. Arnau Dataset.

Participants performed a cued task-switching experiment during the EEG recording. On each trial, participants saw a cue for the tilt vs color task for 200 ms, followed by 600 ms delay. In the trial phase, participants saw an array of gratings, and had to report the tilt or the color of a singleton, depending on the cued task. Participants had up to 1200 ms to respond, and then there was an inter-trial interval (800–1000 ms). As in Hall-McMaster, participants practiced the task before the main experiment, which consisted of eight experimental blocks with 256 trials each. This experiment also investigated the role of motivatoin on task switching, with a block-level incentive manipulation. However, this similarity is coincidental, due to motivation being an area of focus in recent high-quality experiments on cognitive control and task switching.

Here, EEG data were recorded using 128 passive Ag/AgCl electrodes (Easycap GmbH, Herrsching, Germany) with a NeurOne Tesla AC-amplifier (Bittium Biosignals Ltd., Kuopio, Finland). The EEG were preprocessed using EEGLab scripts modified from the original experiment in three ways. First, we resampled the data at 125 Hz (as in Hall-McMaster). Second, we used Blackman zero-phase FIR filtering, rather than Butterworth IIR filtering. We separately high-pass filtered at 0.1 Hz and low-pass filtered at 30 Hz, using the default EEGLab filter order for each frequency. Finally, we used more lenient inclusion criteria for the ICA auto-rejection procedure, as some participants had the majority of their components removed. We used the same performance-based inclusion criteria as in Hall-McMaster. After preprocessing, we split up the experimental blocks into training and test sets, with Mean(SD) = 676(147) training trials and 142(31) testing trials. See (58) for full details on the task and original preprocessing procedure.

### 3. Encoding Geometry Analysis

In the traditional multivariate analyses, we used Encoding Geometry Analysis (EGA; (82)) to quantify task encoding. At each timestep within the epoch, we fit a general linear model (GLM) to the across-trial voltage in the EEG electrodes. The regression model included an intercept, the current task, the previous task, cue identity (with separate regressor contrasting the cues within each task), cue repetitions vs alternations for repeat trials, the main effect of switch vs repeat, the current trial’s reaction time, and the previous trial’s reaction time. We fit this GLM separately to even and odd runs, and then performed multivariate spatial prewhitening on the regression estimates (83). We tested the reliability of regression weights by correlating spatial patterns across even and odd folds, which is closely related to out-of-sample predictive accuracy (82).

### 4. Multiple comparisons corrections

To correct for multiple comparisons over time while accounting for temporal autocorrelation, we used threshold-free cluster enhancement through-out (TFCE; (52)) with a set of EEG-optimized parameters (H=2, C=1; from (52); 1,000 permutations for temporal generalization and 10,000 permutations for traces).

### 5. Latent Linear Dynamical System

#### A. Generative Model.

We developed a toolkit called StateSpaceAnalysis for fitting linear-Gaussian state space models (SSMs) to neuroimaging data. Our goal was to test how effectively SSMs could capture rich neuroimaging data, balancing the predictive power of machine learning approaches with the interpretability offered by linear-Gaussian assumptions. To provide the speed and precision needed for estimating the parameters of these large latent variable models, we developed this package using the open-source coding language Julia, available at https://github.com/harrisonritz/StateSpaceAnalysis.jl (45).

The generative model for the analysis is a partially observable autoregressive process: a linear dynamical system from which we can only make noisy measurements (or, equivalently, that only produces noisy emissions of its underlying state). This autoregressive process consists of a vector of latent factors (x), and a discrete-time difference equation that describes how they evolve:

$$x_{t}=Ax_{t-1}+Bu_{t-1}+w_{t}$$

$$x_{t=1}=B_{0}u_{0}+w_{1}$$

Latent factors evolve according to their recurrent dynamics (Ax), the influence of known inputs (Bu), and process noise ($w_{t}\;\sim 𝒩(0,W)$). The initial conditions depend on the trial conditions $\left(B_{0}u_{0}\right)$ and initial uncertainty ($w_{1}\;\sim \;𝒩\left(0,W_{1}\right)$).

At each timestep, we get a noisy observation of these latent factors:

$$y_{t}=Cx_{t}+v_{t}$$

Observations are projections of the latent factors (Cx) corrupted by observation noise $\left(v_{t}\;\sim \;𝒩(0,V)\right)$. Note that process noise is carried forward in time through the recurrent dynamics, unlike observation noise.

#### B. Expectation-Maximization.

A major benefit of SSMs are that they allow closed-form methods for Bayesian inference of the underlying latent state, and closed-form marginal likelihood of the observed data, $P\left(y_{1:T}|\Theta \right)$ where Θ represents the model parameters. This is achieved using standard inferential tools from control theory: Kalman filtering (inferring $x_{t}$ from past observations) and Rauch–Tung–Striebel (RTS) smoothing (inferring $x_{t}$ using both past and future observations).

While the marginal likelihood from Kalman filtering would allow us to directly fit the parameters through techniques like maximum likelihood estimation, in practice it is usually much more efficient to use expectation-maximization (EM; (43, 44, 84)). EM maximizes the expected lower bound of the marginal posterior (often called the evidence lower bound or ELBO) by alternating between two steps. In the E-step, we estimate the latent variables using the RTS smoother. In the M-step, we find a point-estimate of the parameters that maximize the ELBO, given the estimated latent states. With priors on the parameters, this optimizes the mode of their posterior density. The Linear-Gaussian assumptions of this model considerably simplify this procedure by allowing us to work with the sufficient statistics of the estimated latent state (i.e., their mean and covariance).

##### B.1. Marginal Log Posterior.


$$ℒ=E\left[log𝒩\left(x_{1};B_{0}u_{0},W_{1}\right)\right]+E\left[log𝒩\left(x_{t};Ax_{t-1}+Bu_{t-1},W\right)\right]+E\left[log𝒩\left(y_{t};Cx_{t},V\right)\right]\;\;+log𝒩\left(A;0,\Lambda_{A}\right)+log𝒩\left(B;0,\Lambda_{B}\right)+log𝒩\left(C;0,\Lambda_{C}\right)$$

##### B.2. E-Step.

Using the filter-smoother algorithm, we estimated the posterior mean $\left(m_{t}\right)$ and covariance $\left(\Sigma_{t}\right)$ of the latent state across timesteps (t) and epochs (n), generating a set of sufficient statistics:

$$M_{\left(t_{1},t_{2}\right)}=\sum_{n=1}^{N}\;\sum_{t=t_{1}}^{t2}\;m_{n,t}m_{n,t}^{⊤}+\Sigma_{t}\;M_{\Delta }=\sum_{n=1}^{N}\;\sum_{t=2}^{T}\;m_{n,t-1}m_{n,t}^{⊤}+\Sigma_{t-1,t}\;\;Y_{y}=\sum_{n=1}^{N}\;\sum_{t=1}^{T}\;y_{n,t}y_{n,t}^{⊤}\;Y_{\Delta }=\sum_{n=1}^{N}\;\sum_{t=1}^{T}\;m_{n,t}y_{n,t}^{⊤}\;U_{u}=\sum_{n=1}^{N}\;\sum_{t=1}^{T-1}\;u_{n,t}u_{n,t}^{⊤}\;U_{m}=\sum_{n=1}^{N}\;\sum_{t=1}^{T-1}\;m_{n,t}u_{n,t}^{⊤}\;U_{\Delta }=\sum_{n=1}^{N}\;\sum_{t=1}^{T-1}\;m_{n,t+1}u_{n,t}^{⊤}\;U_{u_{0}}=\sum_{n=1}^{N}\;u_{n,0}u_{n,0}^{⊤}\;U_{\Delta_{0}}=\sum_{n=1}^{N}\;m_{n,1}u_{n,0}^{⊤}$$

Note that we can use the same smoothed covariance estimates (Σ) for all epochs, substantially speeding up the computation.

##### B.3. M-Step.

We then use the computed sufficient statistics to update the parameters using a maximization procedure similar to ordinary least squares. Note that we include priors (Λ) on all of the dynamics matrices (scaled identity matrices).

For the dynamics terms:

$$\left[\begin{array}{c} A\\ B \end{array}\right]\leftarrow {\left[\begin{array}{cc} M_{\left(1,T-1\right)}+\Lambda_{A} & U_{m}^{⊤}\\ U_{m} & U_{\left(1,T-1\right)}+\Lambda_{B} \end{array}\right]}^{-1}\left[\begin{array}{c} M_{\Delta }\\ U_{\Delta } \end{array}\right]\;C\leftarrow {\left(M_{\left(1,T\right)}+\Lambda_{C}\right)}^{-1}Y_{\Delta }\;B_{0}\;\leftarrow {\left(U_{u_{0}}+\Lambda_{B_{0}}\right)}^{-1}U_{\Delta_{0}}$$

For the covariance terms:

$$W\leftarrow \frac{1}{N(T\;-\;1)\;-\;D_{x}}\left(M_{\left(2,T\right)}+AM_{\left(1,T-1\right)}A^{⊤}+BU_{m}B^{⊤}-AM_{\Delta }-M_{\Delta }^{⊤}A-BU_{\Delta }-U_{\Delta }^{⊤}B\right.\;\;\left.\;+BU_{m}A^{⊤}+AU_{m}^{⊤}B^{⊤}+A\Lambda_{A}A^{⊤}+B\Lambda_{B}B^{⊤}\right)\;\;B\leftarrow \frac{1}{NT\;-\;D_{y}}\left(Y_{y}+CM_{\left(1,T\right)}C^{⊤}-CY_{\Delta }-Y_{\Delta }^{⊤}C+C\Lambda_{C}C^{⊤}\right)\;W_{0}\leftarrow \frac{1}{N\;-\;D_{x}}\left(M_{\left(1,1\right)}+B_{0}U_{u_{0}}B_{0}^{⊤}-B_{0}U_{\Delta_{0}}-U_{\Delta_{0}}^{⊤}B_{0}+B_{0}\Lambda_{B_{0}}B_{0}^{⊤}\right)$$

With $D_{x}$ and $D_{y}$ indicates the numbers of factors and observation dimensions, respectively. Note that the estimator of $W_{0}$ is a little unusual, but is equivalent to (43) when the only input is an intercept term. Alternating between these E-steps and M-steps monotonically improves the ELBO towards a local optimum (i.e., depended on the initialization; see ‘Subspace Identification’ below). This toolkit is inspired by the SSM and dynamax python packages, but had several optimizations specifically tailored for linear-Gaussian systems (e.g., positive definite matrix classes).

#### C. Subspace Identification.

We found that good initialization of the parameters was critical to the effectiveness and stability of the model-fitting procedure. We initialized the parameters using a procedure called subspace identification (SSID; (85, 86)). This procedure constructs a large delay embedding matrix which concatenates lagged future and past copies of the observations (y) and inputs (u). The ‘horizon’ of these lags was set to match the largest tested model (see below). We used the canonical variate analysis procedure (87), which uses SVD to find low-dimensional mappings between lagged inputs and outputs. We can recover the A and C matrices by processing different blocks of this SVD, and then estimate the rest of the parameters using fixed-gain Kalman filtering. We performed CVA using a modified version of the ControlSytemIdentification.jl Julia package (Method 13).

## 6. Fitting procedure

### A. Preprocessing.

We followed the same procedure for the Hall-McMaster and Arnau datasets. We split the data into training and testing folds, preprocessing the inputs and observations of each separately. We epoched the recordings around the preparatory period, including the cue, the delay period, and the first 200 ms of the trial (before any RTs had occurred, due to the short-RT trial rejection).

The SSM inputs included an intercept (i.e., effect of time), the current task, the previous task, the cue identity (with separate regressor contrasting the cues within each task), cue repetitions vs alternations for repeat trials, task switch vs task repeat, the upcoming trial’s reaction time, and the previous trial’s reaction time. For the initial conditions, we included an intercept, the previous task, the previous reaction time, and the upcoming reaction time. We convolved the predictors with a cubic B-spline basis set. Spline knots were placed to optimize coverage at every 5 timesteps, tiling the entire epoch (Hall-McMaster: 20 bases per predictor, Arnau: 25 bases per predictor). Each input was z-scored across trials to standardize and reduce collinearity.

We preprocessed the EEG electrodes using PCA, projecting the voltage timeseries into PCs accounting for 99% of the variance. We used PCA because the observations were degenerate due to spatial proximity, independent components analysis, and bad-channel interpolation. PCA also reduced the computational demands of this analyses, reducing the observation dimensionality and making the observation covariance closer to diagonal. We estimated the PCs using only the training data, and then used these components to project the test data.

### B. SSID.

To provide the best initialization across the latent dimension hyper-parameter, we estimated SSID for a horizon and latent dimensionality that matched the largest tested model (128 latent factors). We then truncated these systems for smaller models (as latent states from CVA are ordered by their singular values). We found that SSID was enhanced by reshaping the trial-wise data into a long timeseries (e.g., allowing for larger numbers of factors and low frequencies). During SSID, we also only included inputs in the initial timesteps of each epoch, reducing the collinearity between lagged inputs. Note that test data we removed before this process (which were on separate experimental blocks to minimize temporal proximity), and the EM procedure was fit within each epoch and with all inputs. In practice, we found that this procedure provided a good initial guess of the parameters without requiring multiple initializations, which was especially important when there were more factors than observation dimensions.

### C. Expectation Maximization.

As described above, the EM procedure cycled between estimating the latent states, and updating the parameters. We fit the model across eight different levels of latent dimensionality: (16, 32, 48, 64, 80, 96, 112, 128). We set the maximum number of iterations to 20,000, measuring the test-set log-likelihood every 100 iterations. We terminated the fitting procedure if either the total data likelihood in the training set stopped decreasing, or if the test log-likelihood stopped decreasing.

### D. Parameter Recovery.

We validated that the combination of model, data, and fitting procedure could produce identifiable parameter estimates when we know the generative model class. First, we used the SSM generative model to create a synthetic dataset based on a participant’s parameters and inputs. We then performed on this synthetic dataset. Next, we aligned the estimated and recovered parameters, as the estimates from are only identifiable up to an invertible transformation. Intuitively, we could shuffle the ordering of the latent factors without changing the likelihood. Finally, we correlated the generating and recovered parameters (correlating the Cholesky factors for covariance matrices). While we had somewhat poorer recovery of input matrices (Fig. 1D), this strongly depended on the norm of the input matrix column that we were trying to recover (i.e., some predictors were weakly encoded, and these were difficult to recover), and also likely reflects the correlation between temporal bases.

## 7. Model Comparison

### A. Bayesian Model Selection.

We selected the number of latent dimensions for subsequent analyses using a standard Bayesian model selection procedure (48). This analysis estimates the expected probability of each model in the population. From these posterior mixture weights, we computed a ‘protected exceedance probability’, the probability that a model is the most popular within the tested set of models, relative to chance.

### B. Sensor-Level Null Models.

To compare to a set of simpler null hypotheses, we fit a set of four models directly to the electrode PCs (i.e., without a latent embedding beyond the PCA). First was an intercept-only model (for standard $R^{2}$). Second was an encoding analysis using the full temporal basis set (a generalized additive model similar to EEG methods like the unfold toolbox, (88)). Third was a vector autoregressive (VAR(1)) model, estimating the multivariate relationship between sensor PC responses on adjacent timesteps. Fourth was a model that incorporated both encoding and autoregression. Model four, which included both VAR and encoding, was the best-performing of these null models, so we used this as the benchmark in Figure 2D. We calculated the Cox-Snell $R^{2}$, which uses likelihoods instead of squared errors. This metric is equivalent for normal models with stationary residual covariance (89)), but in this case lets the fit metric account for the predicted covariance.

### C. Neural-Optimized Recurrent Neural Networks.

To compare to a more complex, non-linear model, we predicted the PC timeseries using recurrent neural networks (RNNs) implemented in PyTorch (90)). Each participant was fit with RNNs matched for the number of parameters at each level of SSM factor dimensionality. The inputs to this RNN were the previous PC scores and the same set of predictors as the SSM (formatted as constant inputs over each epoch, which fit better). These inputs were linearly projected into a set of hidden units, along with the previous hidden state, and then passed through a rectified linear unit (ReLU) activation function. The PC score on the next time was linearly decoded from the hidden state using the transpose of the observation embedding layer (a ‘tied’ parameterization; (91)). We found similar performance when we fit both the encoding and decoding layers, as these required more parameters. We also fit an initial hidden state, which depended on the previous task.


$$h_{t}=ReLU\left(x_{t-1}W_{ih}^{T}+h_{t-1}W_{hh}^{T}+b_{hh}\right)$$

$$h_{t=1}=x_{0}W_{0}^{T}$$

$$\overset{ˆ}{y}=h_{t}W_{ih[y]}$$

Note that for consistency with PyTorch, we use $h_{t}$ to refer to the latent state (SSM: $x_{t}$), and $x_{t}$ to refer to the inputs (SSM: $u_{t}).\;W_{ih[y]}$ refers to the columns of $W_{ih}$ used to encode the previous observations.

We fit 32 random initializations of the RNN to each participant and factor size. Each fitting session involved 5000 epochs of updating the parameters by backpropagating through time the mean square error of the next-timestep prediction. Each batch contained all of the training data. Parameters were updated using the ‘AdamW’ learning rule (learning rate = .001, weight decay = .01; (92)). To evaluate each model’s performance, we took the best test-set loss across all epochs and initializations.

## 8. Task State Analysis

### A. Additive state decomposition.

To isolate the dynamics of task representations, we leveraged a powerful property of linear systems: the superposition principle. SSMs can be factorized into an additive set of subsystems for each input, a procedure known as ‘additive state decomposition’ (51). We used this principle to model ‘task subsystems’ in which state dynamics are only caused by task-related inputs. We modeled task inputs for switch using the difference between ‘current task’ and ‘previous task’ predictors, and task inputs for repeat trials using their sum. This allowed us to further decompose the system into a switch-task subsystem and a repeat-task subsystem. Within each subsystem, the state evolves according to:

$$x_{t}^{\text{task}}=Ax_{t-1}^{\text{task}\;}+B^{\text{task}\;}u_{t-1}^{\text{task}\;}\;x_{t=1}^{\text{task}\;}=B_{0}^{\text{prevTask}\;}u_{0}^{\text{prevTask}\;}$$

Since we contrast-coded the tasks (+1/−1), the dynamics for each task are symmetrical around the origin. For the initial conditions of this task subsystem, we used the ‘previous task’ component of the initial conditions ($B_{0}^{\text{PrevTask}}u_{0}^{\text{PrevTask}}$), which is also symmetrical between switch and repeat trials for the same task. To better equate task states across participants, we normalized the latent space by transforming the parameters by the inverse of the univariate state noise ($Diagonal{\left(W\right)}^{-1}$).

### B. Spatiotemporal Visualization.

Using the decomposition and symmetry principles afforded by the linear system, we compared the estimated latent trajectories of task representations between switch and repeat trials. We first visualized task states using group-level singular value decomposition (SVD). The goal of this visualization method was to provide an aggregate task state that accommodated the different realizations across participants (i.e., that aligned their latent states). In both of the spatial and temporal visualizations, the use of SVD makes the sign of these visualizations arbitrary.

To visualize the latent trajectories over time, we spatially concatenated participants’ switch and repeat task trajectories into a wide 2D ‘temporal’ matrix ((switch timesteps+repeat timesteps)×(factors×participants)). We then used SVD to extract separate scores for the switch and repeat subsystems. Since we only have two tasks, tasks were coded as a contrast, which meant that the trajectories were symmetrical around an interpretable zero point. For illustration purposes, we plotted the trajectories for both tasks.

### C. Temporal Generalization Analysis on Task State.

We performed temporal generalization analysis (93), using a similarity-based approach rather than a decoding-based approach (54). To compute temporal generalization, we computed the cosine of the angle between task states for switch and repeat conditions at every timepoint and temporal lags.

To test whether the temporal generalization of task representations increases over the epoch, we extracted the off-main diagonals of the similarity matrix, which reflect task similarity at different temporal lags. We averaged the diagonals above and below the main diagonal, and then correlated them with a linear time vector (i.e., tested whether the similarity at a given lag increases or decreases over timesteps). After computing these correlations for each participant, we tested for a group-level difference from zero using TFCE.

### D. Task Distance.

To characterize how task states converged (or diverged), we measured the proximity of task states across switch and repeat subsystems. We computed the log Euclidean distance between either (1) when the current task was the same and the previous tasks were different, or (2) when the current task was different and the previous tasks were the same.

### E. Task Velocity.

Within the switch and repeat subsystems, we quantified the velocity of task trajectories. To determine whether velocity differed when states were more convergent, we computed the partial correlation between the same-task proximity and the velocity, controlling for linear and quadratic effects of timestep. For plotting and analysis, we trimmed the first two and last two timesteps, as these had extreme values due to edge artifacts in the temporal basis set (refer to the first and last bases in Fig. 1B).

### F. Performance Alignment.

To test whether the inferred task states mattered for good performance, we explored how trial-level brain states predicted upcoming reaction times. First, we projected each participant’s task trajectory for switch and repeat subsystems back into the observation space ($Cx^{\text{task}}$). On every trial and timestep, we computed the cosine similarity between the task state and the brain response. We then put this similarity into a regression along with predictors accounting for task, switch trials, task × switch interaction, and the 2-norm of the neural response. We tested whether EEG-task alignment was significantly different from zero at the group level using TFCE.

### G. Task Energy.

To quantify the magnitude of task representations, we used Lyapunov functions. These are control theoretic tools used to quantify asymptotic system properties in methods like controllability analysis (37, 55, 94). Controllability quantifies the strength of the input-state coupling. For a time-invariant system, the controllability Gramian $\left(X_{\infty }\right)$ defines the asymptotic state covariance that is attributable to a (white noise) input:

$$X_{\infty }\;=AX_{\infty }A^{⊤}+BB^{⊤}\;\;=\sum_{t=0}^{\infty }\;A^{t}BB^{⊤}A^{t⊤}$$

$X_{\infty }$ can be computed by solving the corresponding Lyapunov equation. To capture the influence of a particular set of time-varying inputs, we simulated the time-resolved trajectory of this Gramian using a recursive Lyapunov function (57):

$$X_{t}^{\text{task}\;}=AX_{t-1}^{\text{task}\;}A^{⊤}+(B^{\text{task}\;}u_{t-1}^{\text{task}\;}){\left(B^{\text{task}\;}u_{t-1}^{\text{task}\;}\right)}^{⊤}\;X_{t=1}^{\text{task}\;}=(B_{0}^{\text{prevTask}\;}u_{0}^{\text{prevTask}\;}){\left(B_{0}^{\text{prevTask}\;}u_{0}^{\text{prevTask}\;}\right)}^{⊤}$$

This task-dependent Gramian measures how much the system changes due to both immediate inputs and the spread of inputs through recurrence. It is similar in spirit to the 2-norm of the task state (34), but additional takes into account the cumulative state change (not just the distance from the origin). We summarized task energy using a standard metric of ‘average controllability’ (37), taking the trace of $X^{\text{task}}$ each timestep.

### H. Model Ablation.

We eliminated different components of the fitted model to assess their contributions to the task state trajectories. We assessed the role of system recurrence by setting the recurrence matrix (A) and initial conditions ($B_{0}u_{0}^{\text{PreviousTask}}$) to zero. We assessed the role of input strength by z-scoring $Bu_{t}^{\text{task}}$ at each timestep.

## 9. Task-Optimized RNNs

### A. Task.

We trained networks to perform an extension of the ‘context-dependent decision-making’ task (CDM; (63, 64, 95, 96), which was designed to be highly similar to the task that participants performed during EEG.

We trained networks on two kinds of sequences. On ‘single trials’, networks performed a classic CDM task. In each sequence, networks first recieved a task cue input (one-hot coded; 10 timesteps), followed by a delay period (20 timesteps). They then got two pairs of stimulus inputs (40 timesteps), having to judge which input in the task-relevant pair had higher amplitude. We trained the networks using a logistic loss function on the correct $\left(y_{i}=1\right)$ and incorrect $\left(y_{i}=0\right)$ response options $\left(-\left(y_{i}log\left(\overset{ˆ}{y_{i}}\right)+(1-\right.\left.y_{i}\right)log\left(1-{\overset{ˆ}{y}}_{i}\right))\right)$.

On ‘double trials’, networks performed two consecutive trials (i.e., without resetting the state). They performed the first trial, had a short intertrial interval (20 timesteps for short-ITI models or 60 timesteps for long-ITI models) and then performed the second trial. Sequences within an epoch had a equal number of target repetitions, distractor repetitions, and task repetitions (64 conditions).

Each epoch contained 150 conditions sets (9600 sequences), each with normally distributed noise (cue d’ = 1.0, stimulus d’ = 0.15). We trained at relatively low SNRs to pressure the networks to find task-relevant states.

### B. Network Architecture.

We trained RNNs with gated recurrent units (GRUs; 108 hidden units; (97)) to perform task-switching. These networks used a standard single-layer RNN architecture: linearly encoded inputs ($x_{t}$), a recurrent hidden state with hyperbolic tangent activation function $\left(h_{t}\right)$, and linear decoded outputs $\left(y_{t}\right)$. The GRU component of these models learn a pair of ‘gates’, each of which encodes the hidden state and inputs, and outputs a sigmoid-constrained gating value that is element-wise multiplied with hidden states. The ‘reset gate’ $\left(r_{t}\right)$ is applied to the hidden state before it is combined with the inputs and passed through the activation function to produce the ‘proposal state’. The update gate $\left(z_{t}\right)$ mixes the proposal state and the previous hidden state. This was implemented through the standard GRU class in PyTorch:

$$r_{t}=\sigma \left(W_{ir}x_{t}+b_{ir}+W_{hr}h_{t-1}+b_{hr}\right)$$

$$z_{t}=\sigma \left(W_{iz}x_{t}+b_{iz}+W_{hz}h_{t-1}+b_{hz}\right)$$

$$n_{t}=tanh\left(W_{in}x_{t}+b_{in}+r_{t}⊙\left(W_{hn}h_{t-1}+b_{hn}\right)\right)$$

$$h_{t}=\left(1-z_{t}\right)⊙n_{t}+z_{t}⊙h_{t-1}$$

$$y_{t}=\sigma \left(W_{ho}h_{t}\right)$$

Note that for consistency with PyTorch, we use $h_{t}$ to refer to the latent state (SSM: $x_{t}$), and $x_{t}$ to refer to the inputs (SSM: $u_{t}$). We trained the GRUs using backpropagation through time, using the AdamW learning rule (learning rate = .01, weight decay = .01).

### C. Curricula.

We trained two groups of GRUs on different curricula. ‘Single networks’ only trained on the single trials (500 epochs). ‘Mixed networks’ trained on single trials for 90% of their training (450 epochs), and then double trials for the final 10% (50 epochs). The rationale for this design was to teach both groups how to perform the task, but give the Mixed networks enough experience that they would learn a compensatory strategy for switching. Note that 450 epoch constituted over-training on the task, at a point where the ‘test loss’ (noise-free loss) was increasing (Fig. S9). We trained 512 networks in each group, using matched random seed to ensure that training was the same for the first 90% of epochs.

### D. Performance Evaluation.

Networks were tested on noise-free double trials to evaluate whether they had learned the underlying task structure and could switch between tasks. We measured the within-sequence performance through the per-timestep loss, averaged over conditions and log transformed. To test for temporal generalization, we evaluated networks on ‘triple trials’, which included a third trial that was absent from training.

### E. Temporal Generalization.

To test the generality of mixed networks’ capacity to switch between task, we explored a curriculum for assessing abstract flexibility. In this curriculum, single trials involved learning four different tasks (mapped to two responses). In double trials, networks switched and repeated only two out of the four tasks. Single networks performed 1000 epochs of single trials, and mixed networks performed 900 single and 100 double. We then assessed both networks’ switching ability on double trials containing the two tasks that had been held out of switching practice, comparing the timestep-resolved loss on switch and repeat trials.

### F. Gate Ablations.

To assess the role of the reset and update gates in GRU’s performance, we trained a series of model ablations. We either fixed the reset gate to be open, fixed the update gate to be open, or trained a vanilla RNN. GRUs were trained with tanh activation function and learning rate = .01, whereas the RNN was trained with a ReLU activation function and learning rate = .001 (which improved its performance). Free parameters were matched across networks (108 hidden units for full model, 134 units for ablated models, 190 units for RNN). We trained these models on 500 epochs of the single trials, and then 500 epochs of the double trials.

### G. Hidden Unit Visualization.

We used a similar group SVD procedure to the EEG (see Method 8) to visualize hidden unit activation during double trials. Using trained networks, we first simulated a set of noisy trials (n=2048) for a single and mixed network (same random seed). Next, we concatenated the hidden unit activations across sequences and networks into a joint matrix ((timesteps×sequences)×( single-network hidden units+ mixed-network hidden units)) Performing SVD on this matrix provides embeddings that captures the common and distinct components of the hidden unit activation, and in practice it produces quite similar trajectories across the two networks. We then averaged the sequences within each task pair and projected these average sequences into the (weighted) right singular vectors corresponding to each network.

## 10. Network Distillation

We distilled the trained RNNs into a high-dimensional SSM, allowing us to more directly compare the task dynamics to human participants. The fitting procedure was closely matched between networks and EEG. We preprocessed training and test trials in a similar way (1536 trials for training, 512 for testing): performing PCA on the hidden unit trajectories (keeping components accounting for 99% of the variance) and constructing a temporal basis for the inputs (10 spline bases over the epoch). Network inputs were a bias, task on switch trials, task on repeat trials, and the main effect of switch. Inputs to initial conditions were a bias and the previous task. We analyzed the preparation for the second trial: cue period (10 timesteps), delay period (20 timesteps), and the initial trial period (10 timesteps).

We fit across five levels of latent dimensionality (16,40,64,88,112), initializing the parameters with SSID at a horizon of 112. We ran for all 512 single networks and mixed networks. Networks were evaluated using the same methods as human participants.

## 11. Quantifying RNN dynamics

## 12. EEG-RNN comparison

To evaluate the similarity between (1) temporal generalization and (2) task energy across networks and humans, we first subsampled human task trajectories (100 or 125 timesteps) to match the duration of networks’ task trajectories (40 timesteps). Next wek then averaged the each measure within humans and RNNs, and then computed the cosine similarity between these vectorized traces across groups (‘congruence coefficient’). To produce a bootstrap estimate, we sampled with replacement 10,000 times from both the RNN and EEG groups, averaging and comparing each sample as above, and then computed the 95th percentiles of this bootstrap distribution.

### A. ITI comparison.

We compared Mixed and Single Networks that had learned under short ITIs (20 timesteps) and Mixed and Single networks that had learned under long ITIs (60 timesteps), using two different methods (N=512 for each combination of network type and ITI).

First, we computed the Euclidean distance between hidden unit activations during the ITI. We used the distance between the average states for each of the two possible previous tasks (i.e., the distances between the post-A states and the post-B states).

Second, we tested whether the networks were using gating to elicit a plausible convergence target state. We used the learned weights to reconstruct the update and reset gates for the post-A and post-B trajectories. For the convergence target, we used the next timestep of the other task. For example, the target during the ITI after task A was $h_{t}^{*}={\overset{ˆ}{h}}_{t+1}^{\text{taskB}}$. This target has the effect of bringing the state to a point of convergence, and we found similar results using the terminal location of the trajectories. We unit-normalized this target to make this comparision more comperable between networks. To test whether these target states were aligned with the gating policy, we took the element-wise multiplication of the gates with the convergence target, and then computed the 2-norm at each timestep. We computed this for the epoch-averaged timeseries, but found similar results when we computed it per-trial and then averaged.

## 13. Software and Visualization

1. Programming Languages
  - Julia (v1.11.2)
  - Python (v3.10.13)
  - Matlab (R2023b)
2. Software Packages
  - PyTorch (v2.4.0; pytorch.org)
  - MatlabTFCE (github.com/markallenthornton/MatlabTFCE)
  - ControlSystemIdentification.jl (v2.10.2; github.com/baggepinnen/ControlSystemIdentification.jl)
  - FieldTrip (98)
  - EEGLAB (v2024.2; (99))
3. Visualization
  - HenriquesLab bioRxiv template (overleaf.com/latex/templates/henriqueslab-biorxiv-template/nyprsybwffws)
  - Scientific Color Maps (100)

The author(s) are pleased to acknowledge that the work reported on in this paper was substantially performed using the Princeton Research Computing resources at Princeton University which is a consortium of groups led by the Princeton Institute for Computational Science and Engineering (PICSciE) and Office of Information Technology’s Research Computing.

## Supplementary Material

Supplement 1

## References

[1] Monsell Stephen. Task switching. Trends Cogn. Sci., 7(3):134–140, March 2003. 12639695 10.1016/s1364-6613(03)00028-7

[2] Kiesel Andrea, Steinhauser Marco, Wendt Mike, Falkenstein Michael, Jost Kerstin, Philipp Andrea M, and Koch Iring. Control and interference in task switching—a review. Psychol. Bull., 136(5):849–874, September 2010. 20804238 10.1037/a0019842

[3] Vandierendonck André, Liefooghe Baptist, and Verbruggen Frederick. Task switching: interplay of reconfiguration and interference control. Psychol. Bull., 136(4):601–626, July 2010. 20565170 10.1037/a0019791

[4] Musslick Sebastian and Cohen Jonathan D. Rationalizing constraints on the capacity for cognitive control. Trends Cogn. Sci., 0(0), July 2021. 10.1016/j.tics.2021.06.001 34332856

[5] Friedman Naomi P and Miyake Akira. Unity and diversity of executive functions: Individual differences as a window on cognitive structure. Cortex, 86:186–204, January 2017. 27251123 10.1016/j.cortex.2016.04.023 PMC5104682

[6] Cepeda Nicholas J, Kramer Arthur F, and Gonzalez de Sather Jessica C M. Changes in executive control across the life span: Examination of task-switching performance. Dev. Psychol., 37(5):715–730, 2001. 11552766

[7] Steyvers Mark, Guy E Hawkins Frini Karayanidis, and Scott D Brown. A large-scale analysis of task switching practice effects across the lifespan. Proc. Natl. Acad. Sci. U. S. A., August 2019. 10.1073/pnas.1906788116 PMC6731761 31427513

[8] Millan Mark J, Agid Yves, Brüne Martin, Bullmore Edward T, Carter Cameron S, Clayton Nicola S, Connor Richard, Davis Sabrina, Deakin Bill, DeRubeis Robert J, Dubois Bruno, Geyer Mark A, Goodwin Guy M, Gorwood Philip, Jay Thérèse M, Joëls Marian, Mansuy Isabelle M, Meyer-Lindenberg Andreas, Murphy Declan, Rolls Edmund, Saletu Bernd, Spedding Michael, Sweeney John, Whittington Miles, and Young Larry J. Cognitive dysfunction in psychiatric disorders: characteristics, causes and the quest for improved therapy. Nat. Rev. Drug Discov., 11(2):141–168, February 2012. 22293568 10.1038/nrd3628

[9] Snyder Hannah R. Major depressive disorder is associated with broad impairments on neuropsychological measures of executive function: a meta-analysis and review. Psychol. Bull., 139(1):81–132, January 2013. 22642228 10.1037/a0028727 PMC3436964

[10] A Allport E A Styles, and Hsieh S L. Shifting intentional set - exploring the dynamic control of tasks. In ATTENTION AND PERFORMANCE XV: CONSCIOUS AND NONCONSCIOUS INFORMATION PROCESSING, pages 421–452. MIT Press, London, England, 1994.

[11] Wylie Glenn and Allport Alan. Task switching and the measurement of “switch costs”. Psychol. Res., 63(3):212–233, August 2000. 11004877 10.1007/s004269900003

[12] Rogers Robert D and Monsell Stephen. Costs of a predictible switch between simple cognitive tasks. J. Exp. Psychol. Gen., 124(2):207, 1995.

[13] Meiran Nachshon. Reconfiguration of processing mode prior to task performance. J. Exp. Psychol. Learn. Mem. Cogn., 22(6):1423–1442, November 1996.

[14] Yeung Nick and Monsell Stephen. Switching between tasks of unequal familiarity: the role of stimulus-attribute and response-set selection. J. Exp. Psychol. Hum. Percept. Perform., 29(2):455–469, April 2003. 12760628 10.1037/0096-1523.29.2.455

[15] Yeung Nick, Leigh E Nystrom, Jessica A Aronson, and Jonathan D Cohen. Between-task competition and cognitive control in task switching. J. Neurosci., 26(5):1429–1438, February 2006. 16452666 10.1523/JNEUROSCI.3109-05.2006 PMC6675498

[16] Schmitz Florian and Voss Andreas. Decomposing task-switching costs with the diffusion model. J. Exp. Psychol. Hum. Percept. Perform., 38(1):222–250, February 2012. 22060144 10.1037/a0026003

[17] Loose Lasse S, Wisniewski David, Rusconi Marco, Goschke Thomas, and Haynes John-Dylan. Switch-independent task representations in frontal and parietal cortex. J. Neurosci., 37(33):8033–8042, August 2017. 28729441 10.1523/JNEUROSCI.3656-16.2017 PMC6596903

[18] Qiao Lei, Zhang Lijie, Chen Antao, and Egner Tobias. Dynamic trial-by-trial recoding of taskset representations in the frontoparietal cortex mediates behavioral flexibility. J. Neurosci., 37(45):11037–11050, November 2017. 28972126 10.1523/JNEUROSCI.0935-17.2017 PMC5678027

[19] Jonathan A Michaels Benjamin Dann, and Scherberger Hansjörg. Neural population dynamics during reaching are better explained by a dynamical system than representational tuning. PLoS Comput. Biol., 12(11):e1005175, November 2016. 27814352 10.1371/journal.pcbi.1005175 PMC5096671

[20] Churchland Mark M and Shenoy Krishna V. Preparatory activity and the expansive null-space. Nat. Rev. Neurosci., 25(4):213–236, April 2024. 38443626 10.1038/s41583-024-00796-z PMC13271173

[21] Ardid Salva and Wang Xiao-Jing. A tweaking principle for executive control: neuronal circuit mechanism for rule-based task switching and conflict resolution. J. Neurosci., 33(50):19504–19517, December 2013. 24336717 10.1523/JNEUROSCI.1356-13.2013 PMC6618764

[22] Musslick Sebastian, Seong Jun Jang Michael Shvartsman, Shenhav Amitai, and Cohen Jonathan D. Constraints associated with cognitive control and the stability-flexibility dilemma. In CogSci. shenhavlab.org, 2018.

[23] Jaffe Paul I, Poldrack Russell A, Schafer Robert J, and Bissett Patrick G. Modelling human behaviour in cognitive tasks with latent dynamical systems. Nature Human Behaviour, pages 1–15, January 2023. 10.1038/s41562-022-01510-8 36658212

[24] Hall-McMaster Sam, Muhle-Karbe Paul S, Myers Nicholas E, and Stokes Mark G. Reward boosts neural coding of task rules to optimize cognitive flexibility. J. Neurosci., 39(43):8549–8561, October 2019. 31519820 10.1523/JNEUROSCI.0631-19.2019 PMC6807286

[25] J D Cohen, Dunbar K, and McClelland J L. On the control of automatic processes: a parallel distributed processing account of the stroop effect. Psychol. Rev., 97(3):332–361, July 1990. 2200075 10.1037/0033-295x.97.3.332

[26] Wallis J D, Anderson K C, and Miller E K. Single neurons in prefrontal cortex encode abstract rules. Nature, 411(6840):953–956, June 2001. 11418860 10.1038/35082081

[27] Woolgar Alexandra, Hampshire Adam, Thompson Russell, and Duncan John. Adaptive coding of task-relevant information in human frontoparietal cortex. J. Neurosci., 31(41):14592–14599, October 2011. 21994375 10.1523/JNEUROSCI.2616-11.2011 PMC6703398

[28] Brown E N, Frank L M, Tang D, Quirk M C, and Wilson M A. A statistical paradigm for neural spike train decoding applied to position prediction from ensemble firing patterns of rat hippocampal place cells. J. Neurosci., 18(18):7411–7425, September 1998. 9736661 10.1523/JNEUROSCI.18-18-07411.1998 PMC6793233

[29] Smith Anne Cand Brown Emery N. Estimating a state-space model from point process observations. Neural Comput., 15(5):965–991, May 2003. 12803953 10.1162/089976603765202622

[30] Macke Jakob H, Büsing Lars, Cunningham John P, Yu Ece Byron M, Shenoy Krishna V, and Sahani Maneesh. Empirical models of spiking in neural populations. In Advances in neural information processing systems, 2011.

[31] Valente Adrian, Ostojic Srdjan, and Pillow Jonathan W. Probing the relationship between latent linear dynamical systems and low-rank recurrent neural network models. Neural Comput., 34(9):1871–1892, August 2022. 35896161 10.1162/neco_a_01522

[32] LaFosse Paul K, Zhou Zhishang, O’Rawe Jonathan F, Friedman Nina G, Scott Victoria M, Deng Yanting, and Histed Mark H. Single-cell optogenetics reveals attenuation-by-suppression in visual cortical neurons. bioRxivorg, page 2023.09.13.557650, May 2024. 10.1073/pnas.2318837121 PMC11551350 39485801

[33] Nozari Erfan, Bertolero Maxwell A, Stiso Jennifer, Caciagli Lorenzo, Cornblath Eli J, He Xiaosong, Mahadevan Arun S, Pappas George J, and Bassett Dani S. Macroscopic resting-state brain dynamics are best described by linear models. Nat. Biomed. Eng., pages 1–17, December 2023. 38082179 10.1038/s41551-023-01117-y PMC11357987

[34] Soldado-Magraner Joana, Mante Valerio, and Sahani Maneesh. Inferring context-dependent computations through linear approximations of prefrontal cortex dynamics. Sci. Adv., 10(51):eadl4743, December 2024. 39693450 10.1126/sciadv.adl4743 PMC11654703

[35] Jha Aditi, Gupta Diksha, Brody Carlos D, and Pillow Jonathan W. Disentangling the roles of distinct cell classes with cell-type dynamical systems. bioRxiv, page 2024.07.08.602520, July 2024. 10.52202/079017-1060 PMC13377537 42491378

[36] Williams Robert L and Lawrence Douglas A. Linear state-space control systems. John Wiley & Sons, Nashville, TN, January 2007.

[37] Tang E and Bassett D S. Colloquium: Control of dynamics in brain networks. Rev. Mod. Phys., 2018.

[38] Lehmann D, Ozaki H, and Pal I. EEG alpha map series: brain micro-states by space-oriented adaptive segmentation. Electroencephalogr. Clin. Neurophysiol., 67(3):271–288, September 1987. 2441961 10.1016/0013-4694(87)90025-3

[39] Linderman Scott, Nichols Annika, Blei David, Zimmer Manuel, and Paninski Liam. Hierarchical recurrent state space models reveal discrete and continuous dynamics of neural activity in C. elegans. bioRxiv, page 621540, April 2019.

[40] Gohil Chetan, Kohl Oliver, Huang Rukuang, Mats W J van Es, Jones Oiwi Parker, Hunt Laurence T, Quinn Andrew J, and Woolrich Mark W. Dynamic network analysis of electrophysiological task data. Imaging Neuroscience, 2:1–19, July 2024. 10.1162/imag_a_00226 PMC12272243 40800366

[41] Hess Florian, Monfared Zahra, Brenner Manuel, and Durstewitz Daniel. Generalized teacher forcing for learning chaotic dynamics. In International Conference on Machine Learning, pages 13017–13049. PMLR, July 2023.

[42] Matthijs Pals A, Sağtekin Erdem, Pei Felix, Gloeckler Manuel, and Macke Jakob H. Inferring stochastic low-rank recurrent neural networks from neural data. arXiv [cs.LG], June 2024.

[43] Ghahramani Zoubin, Rey Geo, and Hinton E. Parameter estimation for linear dynamical systems. Techinical Report, 1996.

[44] Murphy Kevin P. Probabilistic Machine Learning: Advanced Topics. MIT Press, London, England, 2023.

[45] Ritz Harrison. harrisonritz/StateSpaceAnalysis.jl: v0.2.0, 2024.

[46] Churchland Mark M, Yu Byron M, Sahani Maneesh, and Shenoy Krishna V. Techniques for extracting single-trial activity patterns from large-scale neural recordings. Curr. Opin. Neurobiol., 17(5):609–618, October 2007. 18093826 10.1016/j.conb.2007.11.001 PMC2238690

[47] MacDowell Camden J, Briones Brandy A, Lenzi Michael J, Gustison Morgan L, and Buschman Timothy J. Differences in the expression of cortex-wide neural dynamics are related to behavioral phenotype. Curr. Biol., February 2024. 10.1016/j.cub.2024.02.004 PMC10965364 38417445

[48] Stephan Klaas Enno, Penny Will D, Daunizeau Jean, Moran Rosalyn J, and Friston Karl J. Bayesian model selection for group studies. Neuroimage, 46(4):1004–1017, July 2009. 19306932 10.1016/j.neuroimage.2009.03.025 PMC2703732

[49] Steven L Brunton Marko Budišić, Kaiser Eurika, and Nathan Kutz J. Modern koopman theory for dynamical systems. arXiv [math.DS], February 2021.

[50] Durstewitz Daniel, Koppe Georgia, and h Max Ingo. Reconstructing computational system dynamics from neural data with recurrent neural networks. Nat. Rev. Neurosci., pages 1–18, October 2023. 37794121 10.1038/s41583-023-00740-7

[51] Staffans Olof. Encyclopedia of mathematics and its applications: Well-posed linear systems series number 103. Cambridge University Press, Cambridge, England, October 2009.

[52] Smith Stephen Mand Nichols Thomas E. Threshold-free cluster enhancement: addressing problems of smoothing, threshold dependence and localisation in cluster inference. Neuroimage, 44(1):83–98, January 2009. 18501637 10.1016/j.neuroimage.2008.03.061

[53] Stroud Jake P, Duncan John, and Lengyel Máté. The computational foundations of dynamic coding in working memory. Trends Cogn. Sci., 0(0), April 2024. 10.1016/j.tics.2024.02.011 38580528

[54] Luyckx Fabrice, Nili Hamed, Spitzer Bernhard, and Summerfield Christopher. Neural structure mapping in human probabilistic reward learning. Elife, 8, March 2019. 10.7554/eLife.42816 PMC6405242 30843789

[55] Kao Ta-Chu and Hennequin Guillaume. Neuroscience out of control: control-theoretic perspectives on neural circuit dynamics. Curr. Opin. Neurobiol., 58:122–129, September 2019. 31563084 10.1016/j.conb.2019.09.001

[56] Holroyd Clay B. The controllosphere: The neural origin of cognitive effort. Psychol. Rev., February 2024. 10.1037/rev0000467 38358716

[57] Klett Corbin, Abate Matthew, Yoon Yongeun, Coogan Samuel, and Feron Eric. Bounding the state covariance matrix for switched linear systems with noise. In 2020 American Control Conference (ACC), pages 2876–2881. IEEE, July 2020.

[58] Arnau Stefan, Liegel Nathalie, and Wascher Edmund. Frontal midline theta power during the cue-target-interval reflects increased cognitive effort in rewarded task-switching. Cortex, 180:94–110, November 2024. 39393200 10.1016/j.cortex.2024.08.004

[59] Gilbert Sam Jand Shallice Tim. Task switching: a PDP model. Cogn. Psychol., 44(3):297–337, May 2002. 11971634 10.1006/cogp.2001.0770

[60] Brown Joshua W, Reynolds Jeremy R, and Braver Todd S. A computational model of fractionated conflict-control mechanisms in task-switching. Cogn. Psychol., 55(1):37–85, August 2007. 17078941 10.1016/j.cogpsych.2006.09.005

[61] Herd Seth A, O’Reilly Randall C, Hazy Tom E, Chatham Christopher H, Brant Angela M, and Friedman Naomi P. A neural network model of individual differences in task switching abilities. Neuropsychologia, 62:375–389, September 2014. 24791709 10.1016/j.neuropsychologia.2014.04.014 PMC4167201

[62] Cohen J D, Servan-Schreiber D, and McClelland J L. A parallel distributed processing approach to automaticity. Am. J. Psychol., 105(2):239–269, 1992. 1621882

[63] Siegel Markus, Donner Tobias H, and Engel Andreas K. Spectral fingerprints of large-scale neuronal interactions. Nat. Rev. Neurosci., 13(2):121–134, January 2012. 22233726 10.1038/nrn3137

[64] Mante V, Sussillo D, Shenoy K V, and Newsome W T. Context-dependent computation by recurrent dynamics in prefrontal cortex. Nature, 503(7474):78–84, 2013. 24201281 10.1038/nature12742 PMC4121670

[65] Braver Todd Sand Cohen Jonathan D. Chapter 19 dopamine, cognitive control, and schizophrenia: the gating model. In Progress in Brain Research, volume 121 of Progress in brain research, pages 327–349. Elsevier, 1999. 10.1016/s0079-6123(08)63082-4 10551035

[66] O’Reilly Randall Cand Frank Michael J. Making working memory work: a computational model of learning in the prefrontal cortex and basal ganglia. Neural Comput., 18(2):283–328, February 2006. 16378516 10.1162/089976606775093909

[67] Arrington Catherine M, Altmann Erik M, and Carr Thomas H. Tasks of a feather flock together: similarity effects in task switching. Mem. Cognit., 31(5):781–789, July 2003. 10.3758/bf03196116 12956242

[68] van den Wildenberg Wery P M, Wylie Scott A, Forstmann Birte U, Burle Borís, Hasbroucq Thierry, and Richard Ridderinkhof K. To head or to heed? beyond the surface of selective action inhibition: a review. Front. Hum. Neurosci., 4:222, December 2010. 21179583 10.3389/fnhum.2010.00222 PMC3004391

[69] Todorov Emanuel and Jordan Michael I Optimal feedback control as a theory of motor coordination. Nat. Neurosci., 5(11):1226–1235, November 2002. 12404008 10.1038/nn963

[70] Shadmehr Reza and Krakauer John W. A computational neuroanatomy for motor control. Exp. Brain Res., 185(3):359–381, March 2008. 18251019 10.1007/s00221-008-1280-5 PMC2553854

[71] Churchland Mark M, Cunningham John P, Kaufman Matthew T, Ryu Stephen I, and Shenoy Krishna V. Cortical preparatory activity: representation of movement or first cog in a dynamical machine? Neuron, 68(3):387–400, November 2010. 21040842 10.1016/j.neuron.2010.09.015 PMC2991102

[72] Ritz Harrison and Shenhav Amitai. Humans reconfigure target and distractor processing to address distinct task demands. Psychol. Rev., August 2023. 10.1037/rev0000442 PMC11193598 37668574

[73] Athalye Vivek R, Carmena Jose M, and Costa Rui M. Neural reinforcement: re-entering and refining neural dynamics leading to desirable outcomes. Curr. Opin. Neurobiol., 60:145–154, December 2019. 31877493 10.1016/j.conb.2019.11.023

[74] Ritz Harrison, Leng Xiamin, and Shenhav Amitai. Cognitive control as a multivariate optimization problem. J. Cogn. Neurosci., 34(4):569–591, March 2022. 35061027 10.1162/jocn_a_01822 PMC8939373

[75] Friston K J, Harrison L, and Penny W. Dynamic causal modelling. Neuroimage, 19(4):1273–1302, August 2003. 12948688 10.1016/s1053-8119(03)00202-7

[76] Galka Andreas, Yamashita Okito, Ozaki Tohru, Biscay Rolando, and Pedro Valdés-Sosa. A solution to the dynamical inverse problem of EEG generation using spatiotemporal kalman filtering. Neuroimage, 23(2):435–453, October 2004. 15488394 10.1016/j.neuroimage.2004.02.022

[77] Pirondini Elvira, Babadi Behtash, Obregon-Henao Gabriel, Lamus Camilo, Malik Wasim Q, Hamalainen Matti S, and Purdon Patrick L. Computationally efficient algorithms for sparse, dynamic solutions to the EEG source localization problem. IEEE Trans. Biomed. Eng., 65 (6):1359–1372, June 2018. 28920892 10.1109/TBME.2017.2739824

[78] Yan Gang, Vértes Petra E, Towlson Emma K, Chew Yee Lian, Walker Denise S, Schafer William R, and Barabási Albert-László. Network control principles predict neuron function in the caenorhabditis elegans connectome. Nature, 550(7677):519–523, October 2017. 29045391 10.1038/nature24056 PMC5710776

[79] Creamer Matthew S, Leifer Andrew M, and Pillow Jonathan W. Bridging the gap between the connectome and whole-brain activity inC. *elegans*. bioRxiv, page 2024.09. 22.614271, September 2024.

[80] Goldman-Rakic P S. Topography of cognition: parallel distributed networks in primate association cortex. Annu. Rev. Neurosci., 11:137–156, 1988. 3284439 10.1146/annurev.ne.11.030188.001033

[81] Miller E Kand Cohen J D. An integrative theory of prefrontal cortex function. Annu. Rev. Neurosci., 24:167–202, 2001. 11283309 10.1146/annurev.neuro.24.1.167

[82] Ritz Harrison and Shenhav Amitai. Orthogonal neural encoding of targets and distractors supports multivariate cognitive control. Nature Human Behaviour, pages 1–17, March 2024. 10.1038/s41562-024-01826-7 PMC11219097 38459265

[83] Walther Alexander, Nili Hamed, Ejaz Naveed, Alink Arjen, Kriegeskorte Nikolaus, and Diedrichsen Jörn. Reliability of dissimilarity measures for multi-voxel pattern analysis. Neuroimage, 137:188–200, August 2016. 26707889 10.1016/j.neuroimage.2015.12.012

[84] e Holmes Elizabeth, j Ward Eric, and Wills Kellie. MARSS: Multivariate autoregressive state-space models for analyzing time-series data. R J., 4(1):11, 2012.

[85] Smith Gavin, João de Freitas Tony Robinson, and Niranjan Mahesan. Speech modelling using subspace and EM techniques. Advances in Neural Information Processing Systems, 12, 1999.

[86] Stone Iris R, Sagiv Yotam, Park Memming, and Pillow Jonathan W. Spectral learning of bernoulli linear dynamical systems models. arXiv [stat.ML], March 2023. PMC12981602 41836604

[87] Larimore W E. Canonical variate analysis in identification, filtering, and adaptive control. In 29th IEEE Conference on Decision and Control, pages 596–604 vol.2. IEEE, 1990.

[88] Ehinger Benedikt Vand Dimigen Olaf. Unfold: an integrated toolbox for overlap correction, non-linear modeling, and regression-based EEG analysis. PeerJ, 7(e7838):e7838, October 2019. 31660265 10.7717/peerj.7838 PMC6815663

[89] Cox D Rand Wermuth Nanny. A comment on the coefficient of determination for binary responses. Am. Stat., 46(1):1–4, February 1992.

[90] Paszke Adam, Gross Sam, Massa Francisco, Lerer Adam, Bradbury James, Chanan Gregory, Killeen Trevor, Lin Zeming, Gimelshein Natalia, Antiga Luca, Desmaison Alban, Köpf Andreas, Yang Edward, DeVito Zach, Raison Martin, Tejani Alykhan, Chilamkurthy Sasank, Steiner Benoit, Fang Lu, Bai Junjie, and Chintala Soumith. PyTorch: An imperative style, high-performance deep learning library. arXiv [cs.LG], December 2019.

[91] Hinton G Eand Salakhutdinov R R. Reducing the dimensionality of data with neural networks. Science, 313(5786):504–507, July 2006. 16873662 10.1126/science.1127647

[92] Loshchilov Ilya and Hutter Frank. Decoupled weight decay regularization. November 2017.

[93] King J-R and Dehaene S. Characterizing the dynamics of mental representations: the temporal generalization method. Trends Cogn. Sci., 18(4):203–210, April 2014. 24593982 10.1016/j.tics.2014.01.002 PMC5635958

[94] Kalman R E. Lectures on controllability and observability. In Controllability and Observability, pages 1–149. Springer Berlin Heidelberg, Berlin, Heidelberg, 2010.

[95] Ehrlich Daniel Band Murray John D. Geometry of neural computation unifies working memory and planning. Proc. Natl. Acad. Sci. U. S. A., 119(37):e2115610119, September 2022. 36067286 10.1073/pnas.2115610119 PMC9478653

[96] Langdon Christopher and Engel Tatiana A. Latent circuit inference from heterogeneous neural responses during cognitive tasks. Nat. Neurosci., pages 1–11, February 2025. 39930096 10.1038/s41593-025-01869-7 PMC11893458

[97] Cho Kyunghyun, van Merrienboer Bart, Gulcehre Caglar, Bahdanau Dzmitry, Bougares Fethi, Schwenk Holger, and Bengio Yoshua. Learning phrase representations using RNN encoder-decoder for statistical machine translation. arXiv [cs.CL], June 2014.

[98] Oostenveld Robert, Fries Pascal, Maris Eric, and Schoffelen Jan-Mathijs. FieldTrip: Open source software for advanced analysis of MEG, EEG, and invasive electrophysiological data. Comput. Intell. Neurosci., 2011(1):156869, January 2011. 21253357 10.1155/2011/156869 PMC3021840

[99] Delorme Arnaud and Makeig Scott. EEGLAB: an open source toolbox for analysis of single-trial EEG dynamics including independent component analysis. J. Neurosci. Methods, 134(1):9–21, March 2004. 15102499 10.1016/j.jneumeth.2003.10.009

[100] Crameri Fabio, Shephard Grace E, and Heron Philip J. The misuse of colour in science communication. Nat. Commun., 11(1):5444, October 2020. 33116149 10.1038/s41467-020-19160-7 PMC7595127

