# Supplementary material for: Convergent neural dynamical systems for task control in artificial networks and human brains
Source: bioRxiv. 2025 Jul 24:2024.09.29.615736. Preprint. [Version 5] doi: 10.1101/2024.09.29.615736 (PMC11482766; doi:10.1101/2024.09.29.615736)
Supplement: Supplement 1 [file NIHPP2024.09.29.615736v5-supplement-1.pdf]

bioRxiv preprint doi: https://doi.org/10.1101/2024.09.29.615736; this version posted July 24, 2025. The copyright holder for this preprint (which was not certified by peer review) is the author/funder, who has granted bioRxiv a license to display the preprint in perpetuity. It is made available under aCC-BY 4.0 International license.

Supplementary Figures

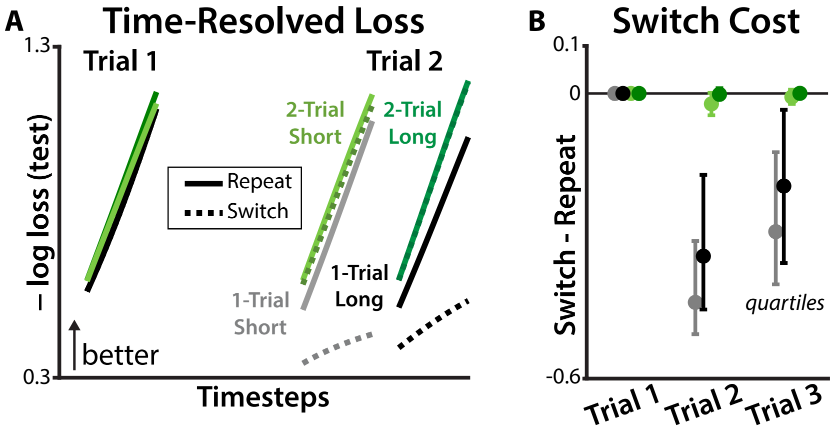

Fig. 1: **Differences in switch costs across RNNs. A)** Time-resolved test loss for each RNN condition, plotted for two trials that either switched task (solid line) or repeated tasks (dashed line). Loss is averaged over networks within each RNN condition; SEM not show because they were similar to or smaller than the line width. Note that the second trial for Long-ITI RNNs are delayed due to their longer ITI. **B)** Difference in the test loss switch and repeat trials, plotted for each RNN condition on 3-trial sequences. Error bars reflect quartiles of the between-RNN distribution.

bioRxiv preprint doi: https://doi.org/10.1101/2024.09.29.615736; this version posted July 24, 2025. The copyright holder for this preprint (which was not certified by peer review) is the author/funder, who has granted bioRxiv a license to display the preprint in perpetuity. It is made available under aCC-BY 4.0 International license.

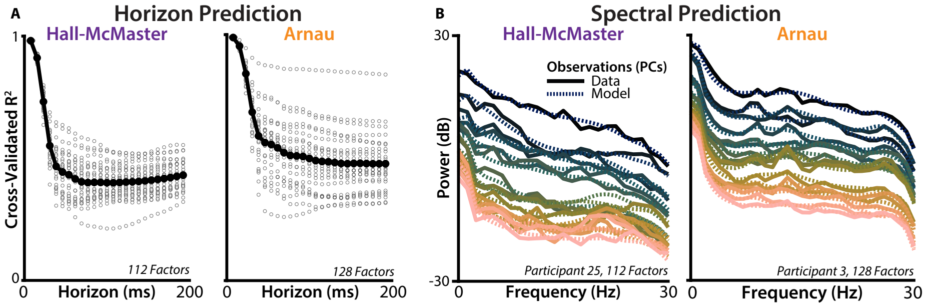

Fig. 2: **Long-range predictive accuracy of SSMs fits to EEG. A)** Predictive accuracy of the model when forecasting at different timesteps into the future. Gray dots indicate individual participants. **B)** Similarity in the power spectrum for test-set observations (solid lines) and model simulations (dashed lines), shown for example participants. Rather than using Kalman filtered predictions, these predictions were generated from 100 posterior samples per participant (i.e., feedforward roll-outs). Power spectra are plotted for each observation dimension (i.e., principal component), aggregated across stimulated epochs using EEGLab's `spectopo` function. Across participants, SSMs accurately captured the empirical power spectra (Hall-McMaster: R^2_{CV} 95% CI [0.97, 0.98]; Arnau: R^2_{CV} 95% CI [0.99, 0.99]). We estimated the the noise ceiling for power spectrum similarity using the similarity between the empirical training-set spectrum and the empirical test-set spectrum, finding that SSMs captured most of the reliable signal (Hall-McMaster: noise-normalized R^2_{CV} 95% CI [.99, .99]; Arnau: noise-normalized R^2_{CV} 95% CI [0.99, 1.0]). There was still high overlap between model and data after mean-centering and linearly detrending the spectra within each principal component (Hall-McMaster: R^2_{CV} 95% CI [0.79, 0.88], noise-normalized 95% CI [0.92, 0.94]; Arnau: R^2_{CV} 95% CI [0.87, 0.93], noise-normalized 95% CI [0.89, 0.97]).

bioRxiv preprint doi: https://doi.org/10.1101/2024.09.29.615736; this version posted July 24, 2025. The copyright holder for this preprint (which was not certified by peer review) is the author/funder, who has granted bioRxiv a license to display the preprint in perpetuity. It is made available under aCC-BY 4.0 International license.

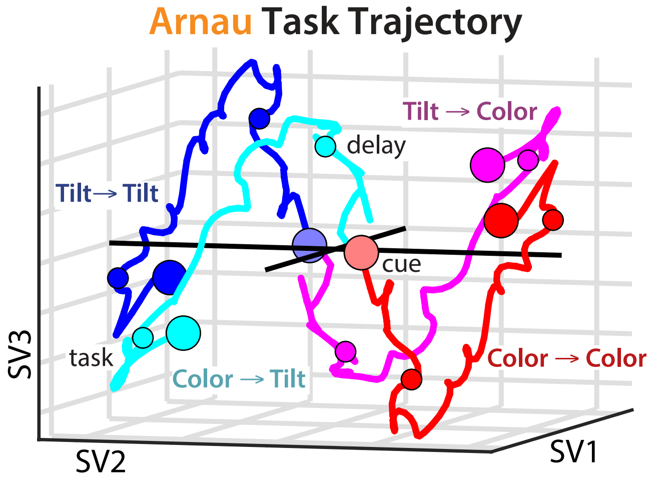

Fig. 3: **Latent task trajectories from SSMs fit to Arnau.** Low-dimensional embedding of the expected task trajectories, split by switch and repeat conditions (SV: singular vector). Task is contrast-coded, so task trajectories within the same switch condition are symmetrical. Shown for Arnau, see Fig. 4C for Hall-McMaster.

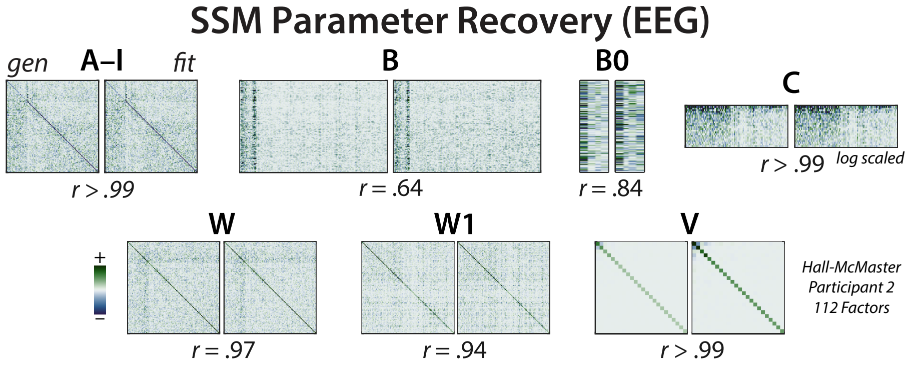

Fig. 4: **SSM parameter recovery in EEG.** Generating and fit parameters for an example participant in Hall-McMaster. Parameters were linearly aligned to account for degeneracy in the latent system Method 4.4.
