# Supplementary material for: Humans actively reconfigure neural task states
Source: bioRxiv. 2025 Feb 28:2024.09.29.615736. Preprint. [Version 4] doi: 10.1101/2024.09.29.615736 (PMC11482766; doi:10.1101/2024.09.29.615736)
Supplement: Supplementary file 1 [file NIHPP2024.09.29.615736v4-supplement-1.pdf]

bioRxiv preprint doi: https://doi.org/10.1101/2024.09.29.615736; this version posted February 28, 2025. The copyright holder for this preprint (which was not certified by peer review) is the author/funder, who has granted bioRxiv a license to display the preprint in perpetuity. It is made available under aCC-BY 4.0 International license.

14. Supplementary Figures

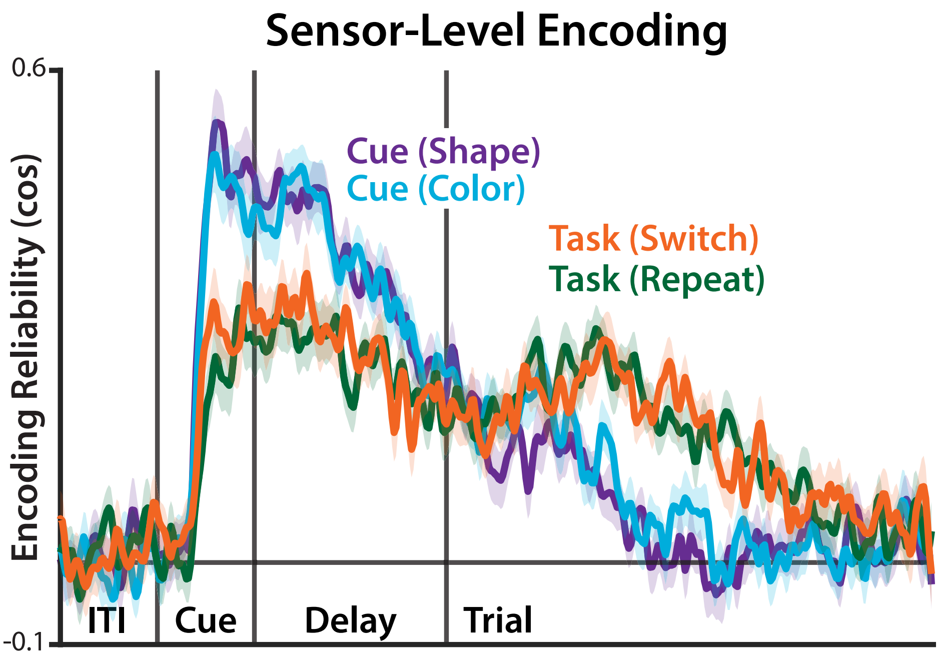

Fig. S1. Sensor-level encoding. Cross-validated encoding reliability of cues for each task (pink and cyan) and tasks for each switch condition (purple and blue). Error bars reflect within-participant standard error. Differences in task encoding strength was not significantly different between switch and repeat conditions.

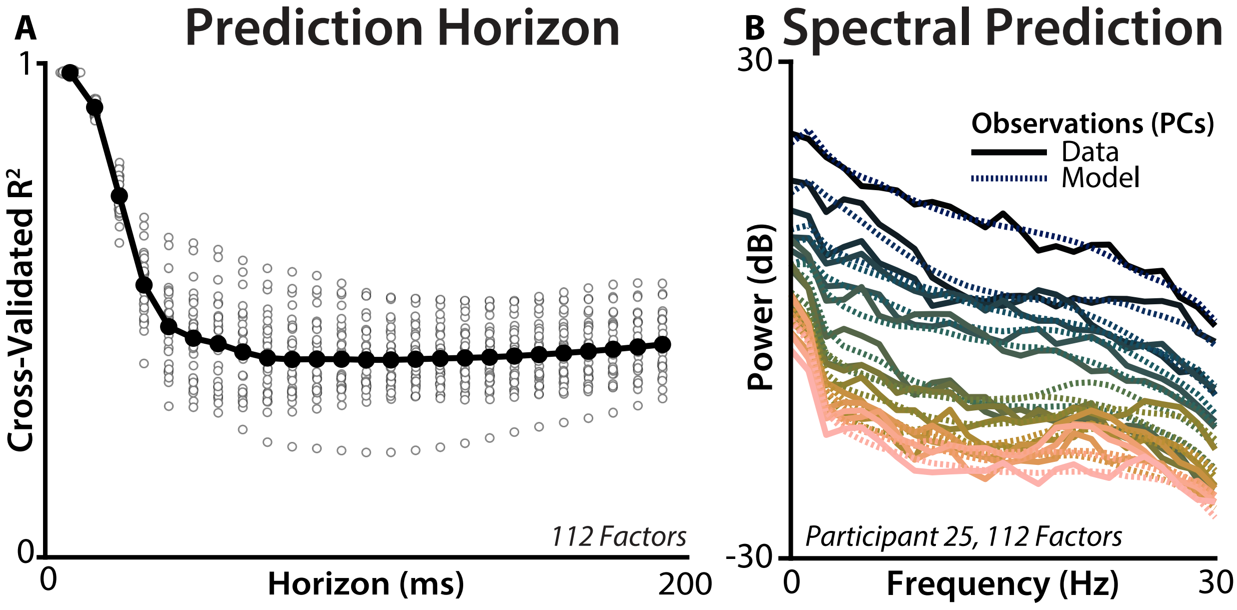

Fig. S2. Extended predictions. **A)** Predictive accuracy of the model when forecasting at different timesteps into the future. White dots reflect individual participants. **B)** Overlap in the power spectrum for test-set observations (solid lines) and model simulations (dashed lines), shown for participant 25. Power spectra are plotted for each observation dimension (i.e., principal component), averaged over epochs using EEGLab's `spectopo` function. Note that these predictions are not filtered (i.e., these are feedforward simulations, averaged over 100 repetitions). Across participants, there was high overlap in the power spectra (median R^2_{CV} = .98; 99% of the noise ceiling, which was estimated by the similarity between training set spectra and test set spectra). There was still high overlap after mean-centering and linearly detrending the spectra within each channel (median R^2_{CV} = .82; 92% of noise ceiling)

bioRxiv preprint doi: https://doi.org/10.1101/2024.09.29.615736; this version posted February 28, 2025. The copyright holder for this preprint (which was not certified by peer review) is the author/funder, who has granted bioRxiv a license to display the preprint in perpetuity. It is made available under aCC-BY 4.0 International license.

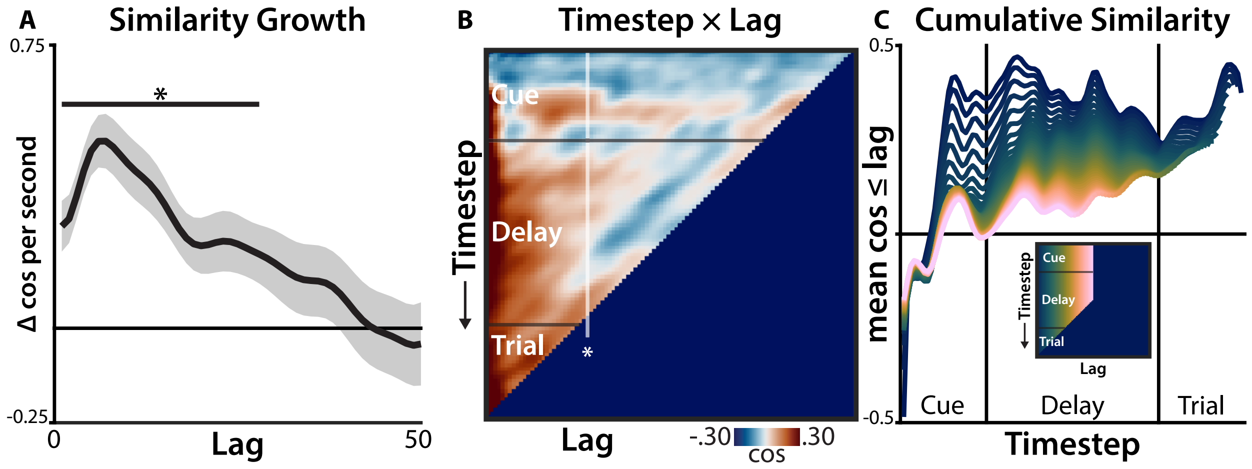

Fig. S3. Change in generalization over the epoch. **A)** Average cosine similarity between task patterns across switch and repeat conditions, plotted at every timestep (y-axis) and time lag (x-axis;compare to Fig. 2C). Vertical white line reflect significance cut-off from (B). **B)** Change in similarity over the epoch (y-axis; linear regression estimate), as a function of lag (x-axis). Similarity change is significantly greater than zero for lags up 31 timesteps (224 ms; TFCE-corrected). Error bars reflect SEM. Corresponds to columns of (A). **C)** At each timestep (x-axis), the cumulative similarity up to each lag (line color; see inset for legend). Corresponds to rows of (A).

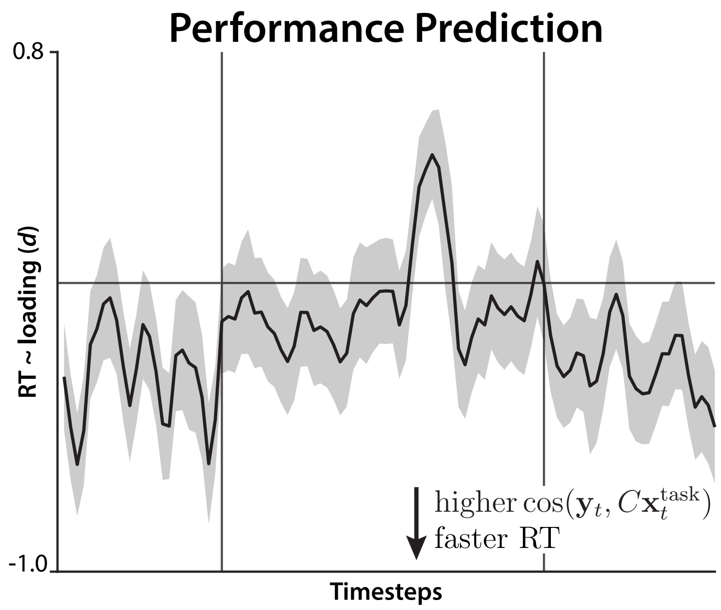

Fig. S4. Relevance of task states for good performance. Group-level effect size (y-axis; Cohen's d) of the individual regression weights mapping 'task loading' on to log reaction time. Task loading is the cosine similarity between EEG activity and the estimated task state. Error bars reflect SEM. No individual timestep is significantly different from zero (using TFCE), however the sum of the regression weights over time is significant ($d = -0.55,$ t-test $p = .0054$), with stronger alignment predicting shorter reaction times.

bioRxiv preprint doi: https://doi.org/10.1101/2024.09.29.615736; this version posted February 28, 2025. The copyright holder for this preprint (which was not certified by peer review) is the author/funder, who has granted bioRxiv a license to display the preprint in perpetuity. It is made available under aCC-BY 4.0 International license.

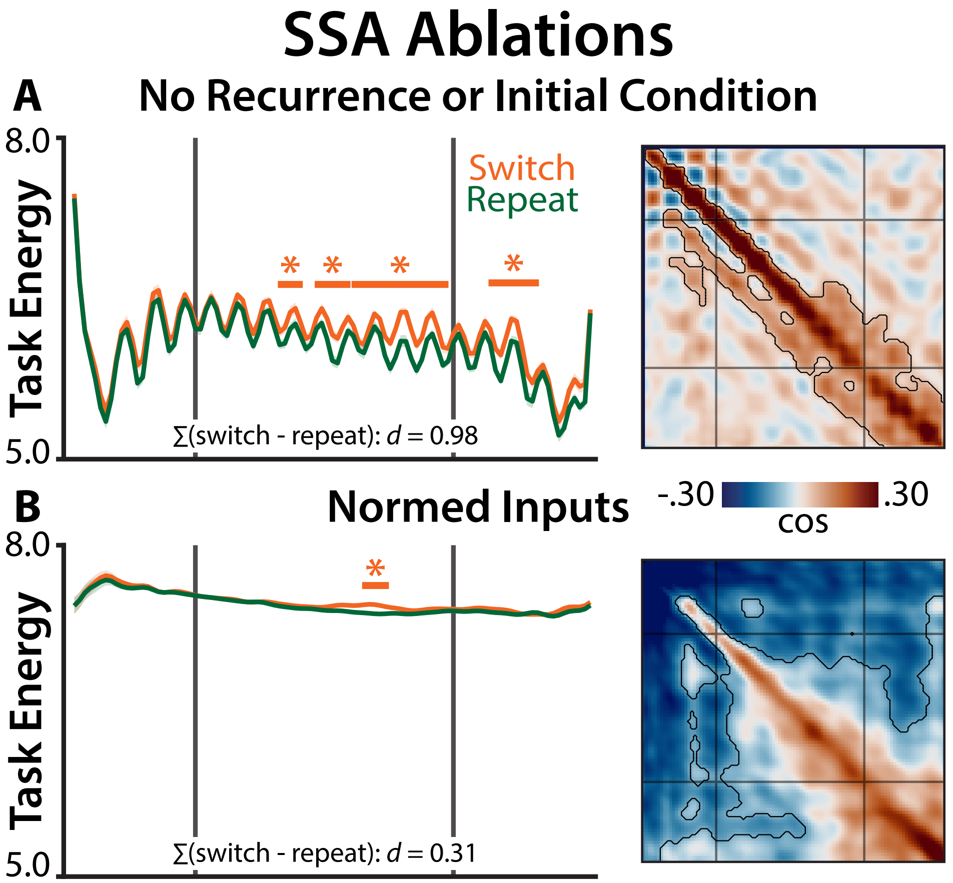

Fig. S5. Ablation. Task energy (left) and temporal generalization (right) for ablated models (generating task states after reduce the influence of fitted parameters). **A)** Removing recurrence (setting A and B_0 to 0) largely preserved differences in task energy across switch conditions, but dramatically changed the pattern of temporal generalization. Note that apparent oscillations are due to the locations of the spline bases. **B)** Removing input magnitude (z-scoring $B^{\text{task}}\mathbf{u}_{t-1}^{\text{task}}$) eliminated the task strength effect, but largely preserved the pattern of temporal generalization (albeit with stronger negative generalization than positive generalization, consistent with interference).

bioRxiv preprint doi: https://doi.org/10.1101/2024.09.29.615736; this version posted February 28, 2025. The copyright holder for this preprint (which was not certified by peer review) is the author/funder, who has granted bioRxiv a license to display the preprint in perpetuity. It is made available under aCC-BY 4.0 International license.

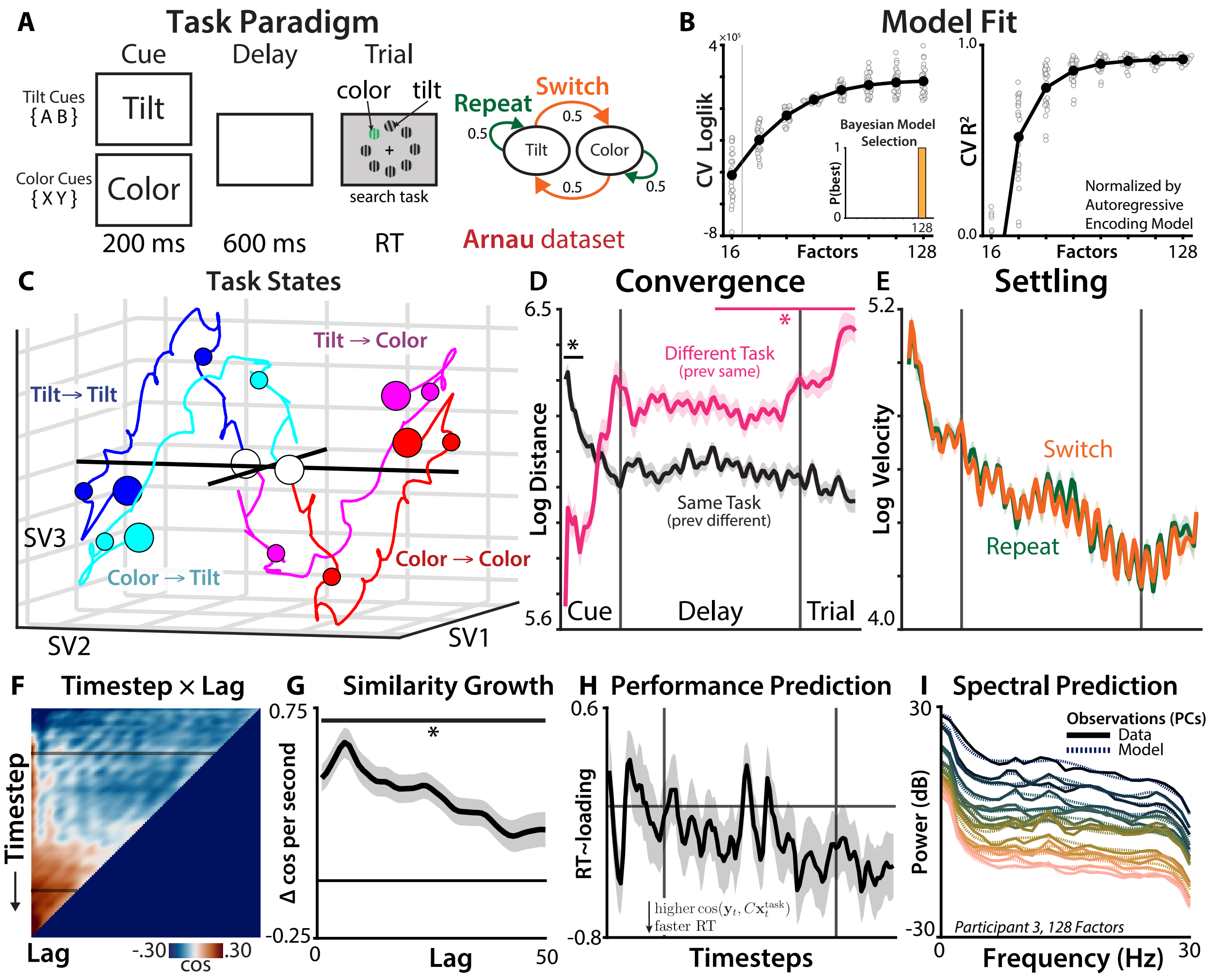

Fig. S6. Replication Dataset ('Arnau'). **(A)** As in Hall-McMaster, participants saw a task cue (two symbols mapped each task), waited through a delay period, then performed the task. Search task required participants to find the colored grating or the tilted grating. Left: The tasks had an equal change of repeating or switching between trials. **(B)** As in Hall-McMaster, models with more factors than observation dimensions provided the best fit. Left, the 128-factor model had the highest CV loglikelihood. Right, the best fitting model had high predictive accuracy ($R^2 = 92\%$, relative to an autoregressive null model). Compare to Fig. 1C,F. **(C)** Low-dimensional projection of the simulated task trajectory. Compare to Fig. 2A. **(D)** Log Euclidean distance between task states for the same task (black) and different tasks (pink). Compare to Fig. 2B. **(E)** Log velocity of task states on switch and repeat trials. Compare to Fig. 2C. **(F)** Generalization of task states at different timepoints (y-axis) and lags (x-axis). Compare to Fig. S3A. **(G)** Linear increase in similarity over time a different lags (columns of (F). Compare to Fig. S3B. **(H)** Group-level effect size (y-axis; Cohen's d) of the individual regression weights mapping 'task loading' (cosine between EEG observation and projected task state) on to log reaction time. Note that weights are not significantly different from zero (summed over time *d* = -0.31, left-tailed *p* = 0.06), however they are significant using a sign-rank test (*p* = 0.0095) and during the trial event (*p* = 0.022). Compare to Fig. S4. **(I)** Prediction of spectral power for test-set observations from open-loop model simulations (centering and detrending within each channel: $R^2 = 0.88$, which is 91% of the noise ceiling). Compare to Fig. S2. Astericks and contours indicate *p* < .05 (TFCE-corrected).

bioRxiv preprint doi: https://doi.org/10.1101/2024.09.29.615736; this version posted February 28, 2025. The copyright holder for this preprint (which was not certified by peer review) is the author/funder, who has granted bioRxiv a license to display the preprint in perpetuity. It is made available under aCC-BY 4.0 International license.

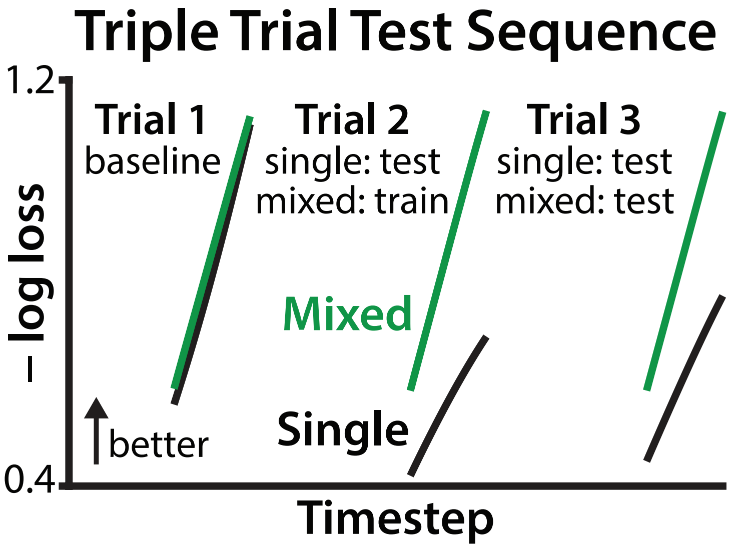

Fig. S7. Sequence generalization. Average test loss across timesteps within a 'triple trial'. Networks were trained on single trials (single networks) or single and double trials (mixed networks), and then tested on this three-trial sequence. SEM smaller than line thickness. Shown for short-ITI model.

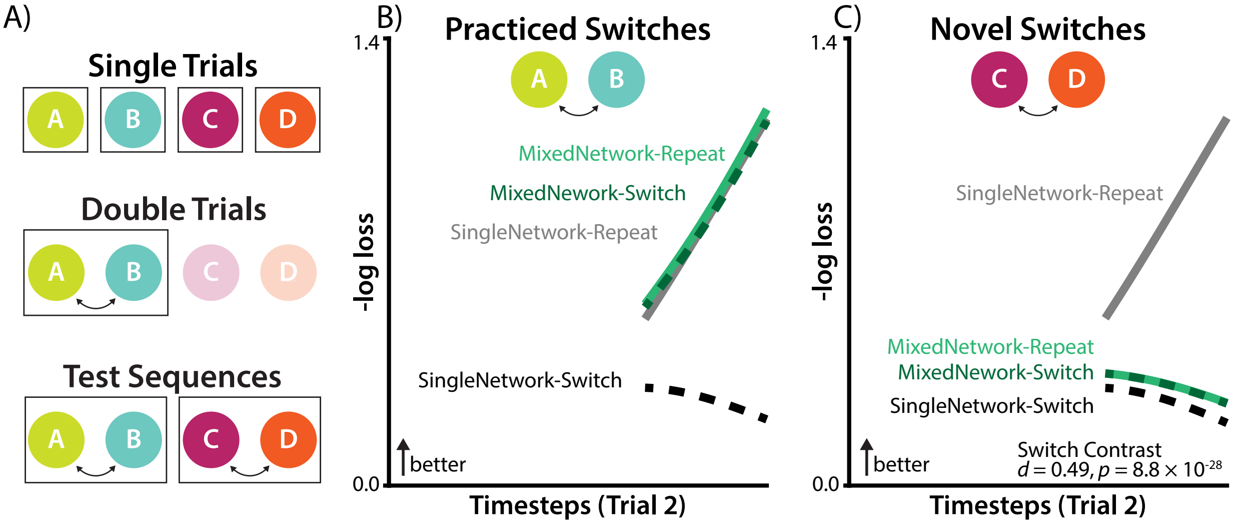

Fig. S8. Task generalization. **A)** We trained GRUs on a modified version of the protocol. In single trials, networks learned four different tasks. In double trials, networks practiced switching between two of the tasks. In test (shown in A and B), we examined switching performance between the practiced and novel task pairs. As in the main experiment, single networks performed only single trials (1000 epochs), and mixed networks were trained on 90% single trials and 10% double trials. **B)** The mixed network shows great performance on task transitions that it had practiced, it (as good at switching as the single network is at repeating). This is similar to the results Figure 4F (shown fore each condition, rather than as contrasts). **C)** On novel task transitions, the mixed network still shows lower switch costs and better performance on switch trials. Note that it has worse performance on repeat trials, likely due to these networks having less experience with C and D tasks (and/or catastrophic interference). Shown for short-ITI model.

bioRxiv preprint doi: https://doi.org/10.1101/2024.09.29.615736; this version posted February 28, 2025. The copyright holder for this preprint (which was not certified by peer review) is the author/funder, who has granted bioRxiv a license to display the preprint in perpetuity. It is made available under aCC-BY 4.0 International license.

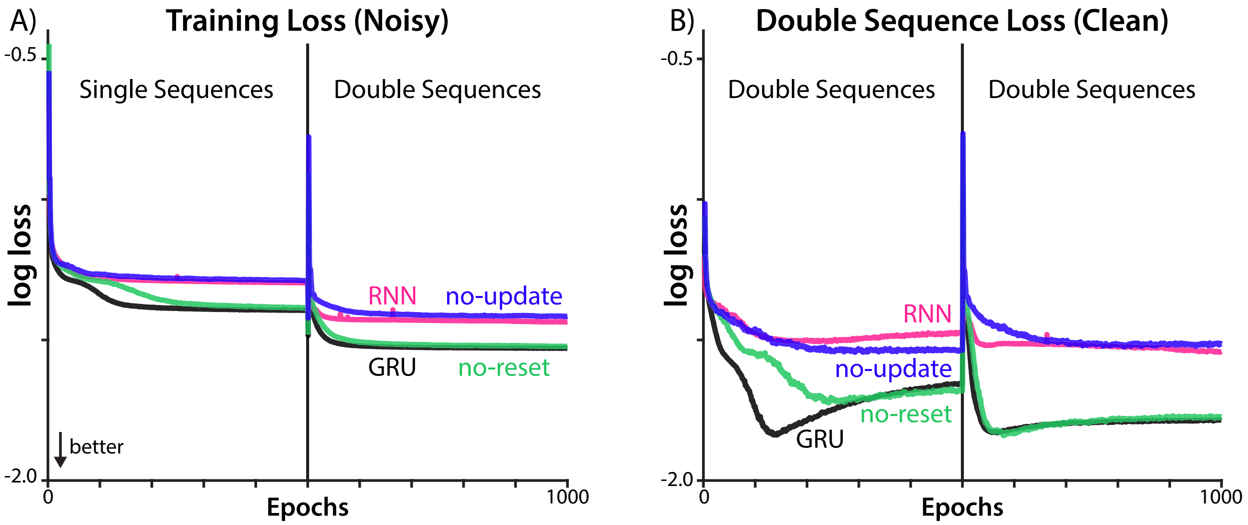

Fig. S9. Gate Ablation. We trained models with all gates ('GRU', black), fixed-open reset gates ('no-reset', green), fixed-open update gates ('no-update', blue), and classic Elman RNNs ('RNN', pink). All models are matched for the number of parameters (see Methods). The first epoch is removed to improve visualization. Note that we plot loss (instead of -loss) to follow convention. **A)** We trained networks on single trials for 500 epochs, and then double trials for 500 epochs. During this training, noise was added to inputs to encourage the learning of a robust policy. During learning, GRU and no-reset models had similar learning trajectories, with better performance than no-update or RNN models. **B)** Network loss was also tested on double trials at every epoch, without input noise. Here too, the GRU and no-reset models had performance gains from experience with the double trials. Note that GRU test loss during the single trials is increasing, suggesting that this is in an over-trained regime. Shown for short-ITI model.

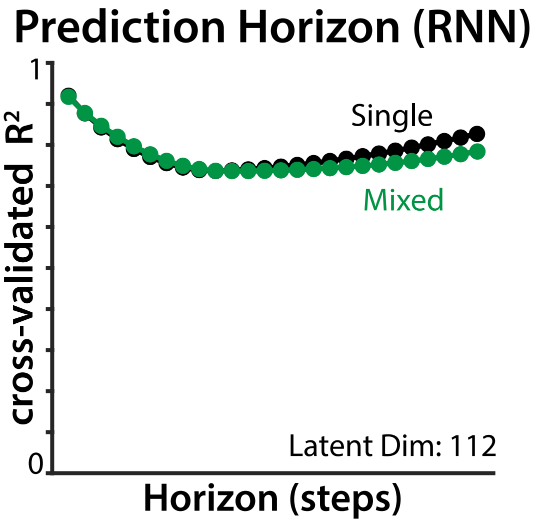

Fig. S10. Prediction horizon for s fit to RNNs. had similarly good predictive accuracy when fit to either single or mixed networks had. This high predictive accuracy was present both at the next timestep (training objective) and at distant temporal lags (25 timesteps is more than half of the epoch). Shown for short-ITI model.

bioRxiv preprint doi: https://doi.org/10.1101/2024.09.29.615736; this version posted February 28, 2025. The copyright holder for this preprint (which was not certified by peer review) is the author/funder, who has granted bioRxiv a license to display the preprint in perpetuity. It is made available under aCC-BY 4.0 International license.

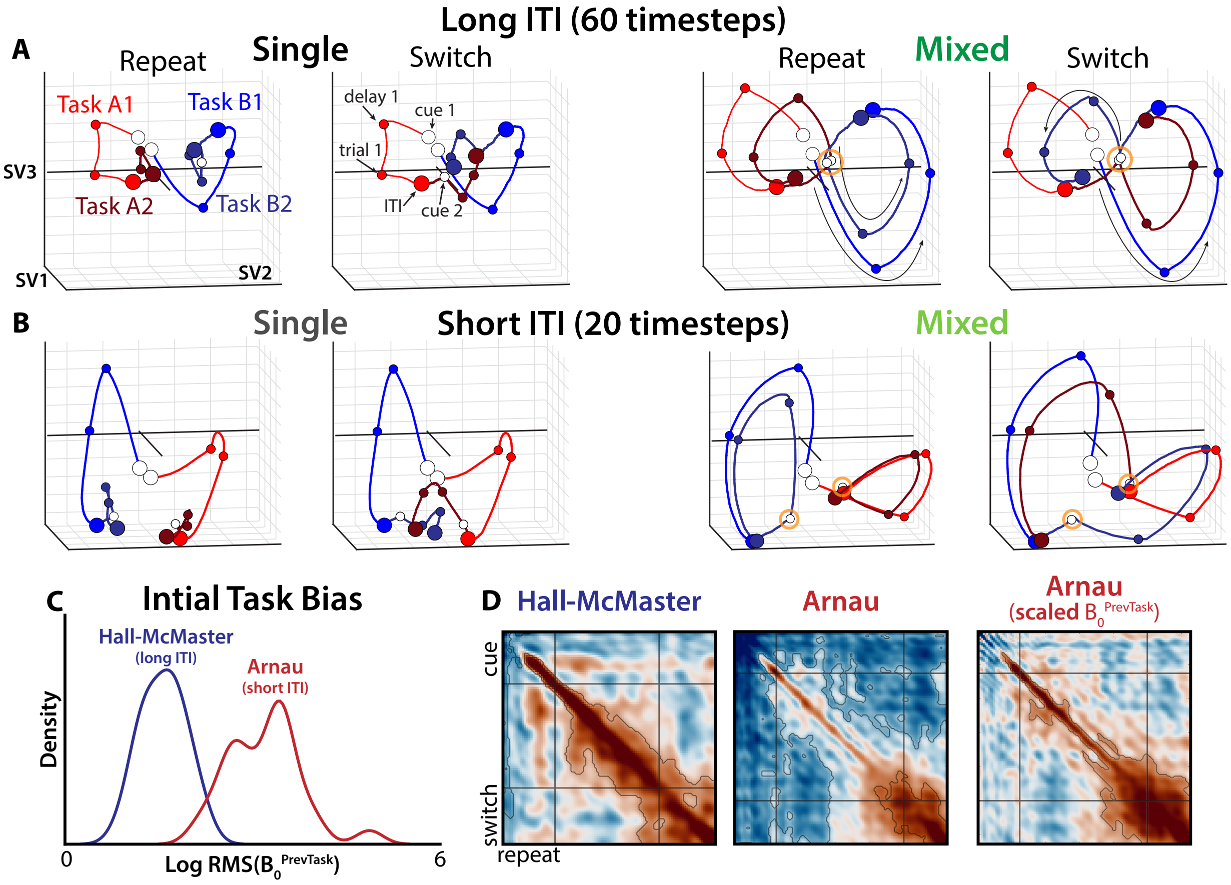

Fig. S11. Comparison between RNNs trained with short and long ITIs. **(A)** Low-dimensional projection of an RRN hidden state for long-ITI networks (same as in Fig. 4). For mixed networks (right two panels), states were close to each other at the time of the second cue. **(B)** RNN hidden state projection for short-ITI networks. Here, states are close to the previous task at the time of the cue. **(C)** Participants in the Hall-McMaster dataset had weaker encoding of 'previous task' in the their initial conditions than participants in the Arnau dataset (both shown at 128 factors). **(D)** Temporal generalization plots for the datasets. The first two plots are from the HallMcMaster and Arnau datasets, shown for reference (same as in Fig. 5). The third panel is from the Arnau dataset, where the magnitude of the initial conditions was rescaled by the ratio between the studies. Rescaling the initial conditions in the Arnau dataset recovers a similar temporal generalization profile as the Hall-McMaster dataset.
