# Supplementary material for: Humans actively reconfigure neural task states
Source: bioRxiv. 2024 Oct 13:2024.09.29.615736. Preprint. [Version 3] doi: 10.1101/2024.09.29.615736 (PMC11482766; doi:10.1101/2024.09.29.615736)
Supplement: Supplementary file 1 [file NIHPP2024.09.29.615736v3-supplement-1.pdf]

bioRxiv preprint doi: https://doi.org/10.1101/2024.09.29.615736; this version posted October 13, 2024. The copyright holder for this preprint (which was not certified by peer review) is the author/funder, who has granted bioRxiv a license to display the preprint in perpetuity. It is made available under aCC-BY 4.0 International license.

## 12. Supplementary Figures

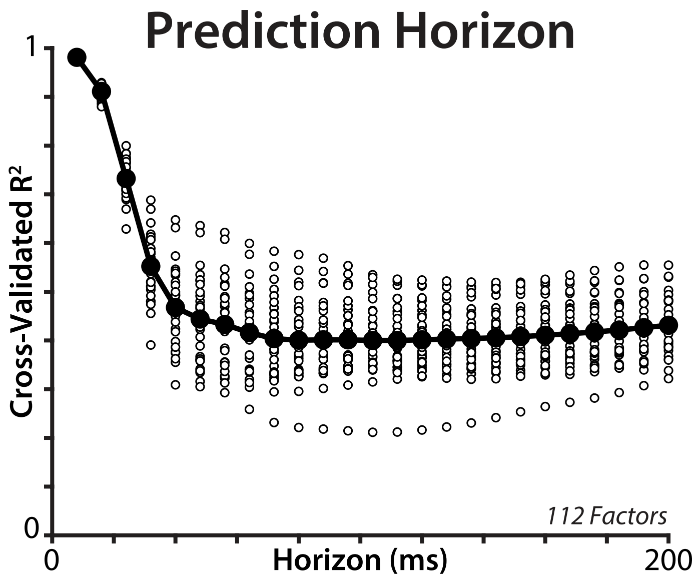


**Fig. S1.** Extended SSA predictions. **A)** Predictive accuracy of the SSA model when forecasting at different timesteps into the future. White dots reflect individual participants. **B)** Overlap in the power spectrum for test-set observations (solid lines) and model simulations (dashed lines), shown for participant 25. Power spectra are plotted for each observation dimension (i.e., principal component), averaged over epochs using EEGLab's `spectopo` function. Note that these predictions are not filtered (i.e., these are feedforward simulations, averaged over 100 repetitions). Across participants, there was high overlap in the power spectra (median $R^2_{CV}$ = .98; 99% of the noise ceiling, which was estimated by the similarity between training set spectra and test set spectra). There was still high overlap after mean-centering and linearly detrending the spectra within each channel (median $R^2_{CV}$ = .82; 92% of noise ceiling)

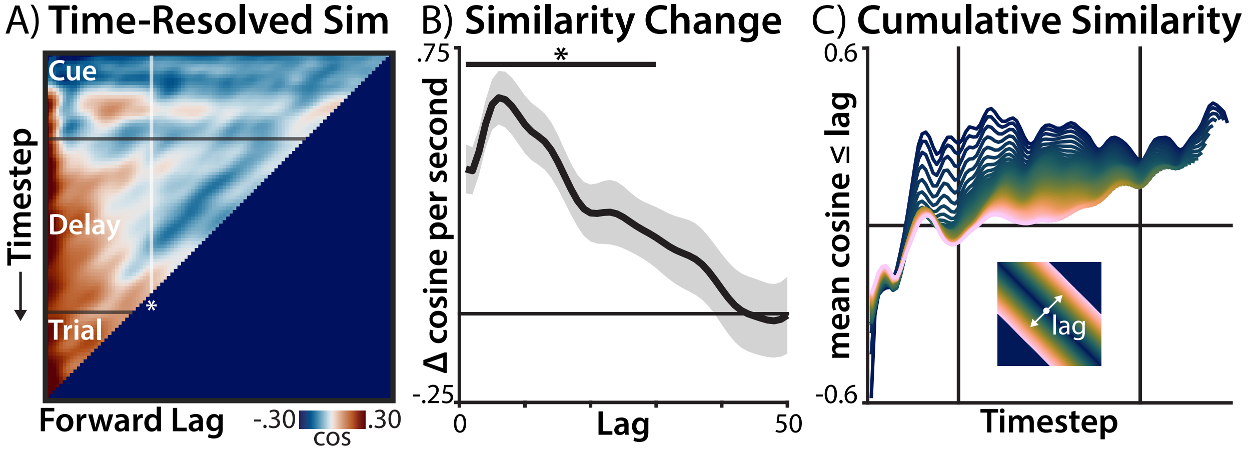


**Fig. S2.** Change in generalization over the epoch. **A)** Average cosine similarity between task patterns across switch and repeat conditions, plotted at every timestep (y-axis) and time lag (x-axis;compare to Figure 3C). Vertical white line reflect significance cut-off from (B). **B)** Change in similarity over the epoch (y-axis; linear regression estimate), as a function of lag (x-axis). Similarity change is significantly greater than zero for lags up 31 timesteps (248 ms; TFCE-corrected). Error bars reflect SEM. Corresponds to columns of (A). **C)** At each timestep (x-axis), the cumulative similarity up to each lag (line color; see inset for legend). Corresponds to rows of (A).

bioRxiv preprint doi: https://doi.org/10.1101/2024.09.29.615736; this version posted October 13, 2024. The copyright holder for this preprint (which was not certified by peer review) is the author/funder, who has granted bioRxiv a license to display the preprint in perpetuity. It is made available under aCC-BY 4.0 International license.

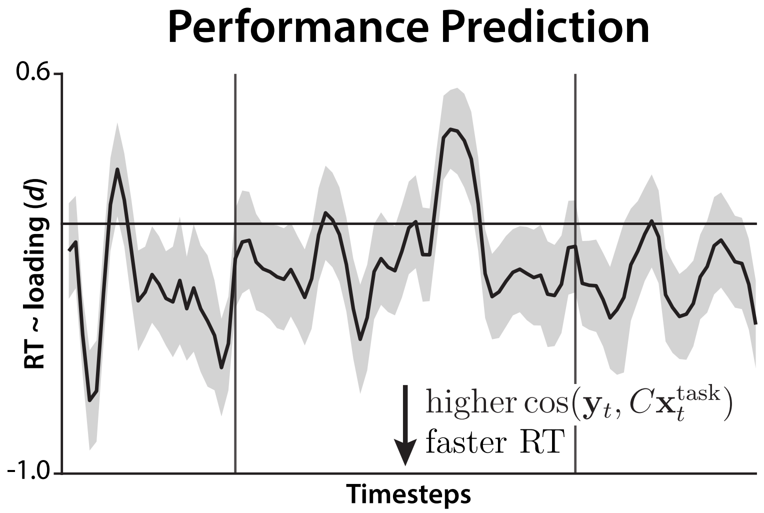


**Fig. S3.** Relevance of task states for good performance. Group-level effect size (y-axis; Cohen's $d$) of the individual regression weights mapping 'task loading' on to log reaction time. Task loading is the cosine similarity between EEG activity and the estimated task state. Error bars reflect SEM. No individual timestep is significantly different from zero (using TFCE), however the sum of the regression weights over time is significant ($d = -0.53,$ t-test $p = .0068$), with stronger alignment predicting shorter reaction times.

bioRxiv preprint doi: https://doi.org/10.1101/2024.09.29.615736; this version posted October 13, 2024. The copyright holder for this preprint (which was not certified by peer review) is the author/funder, who has granted bioRxiv a license to display the preprint in perpetuity. It is made available under aCC-BY 4.0 International license.

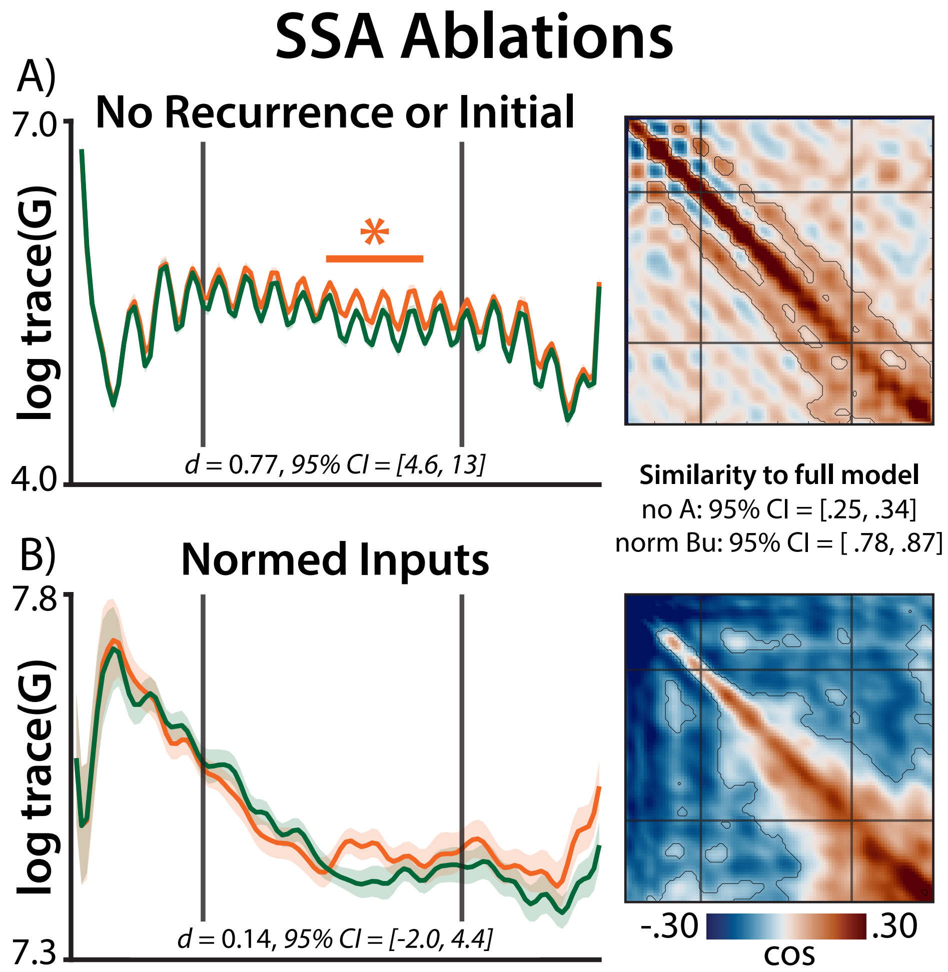


**Fig. S4.** SSA Ablation. Task propagation (left) and temporal generalization (right) for ablated models (generating task states after reduce the influence of fitted parameters). **A)** Removing recurrence (setting $A$ and $B_0$ to 0) largely preserved differences in task propagation across switch conditions, but dramatically changed the pattern of temporal generalization. Note that apparent oscillations are due to the locations of our spline bases. **B)** Removing input magnitude (z-scoring $B^{\text{task}}\mathbf{\Phi}_t$) eliminated the task strength effect, but largely preserved the pattern of temporal generalization (albeit with stronger negative generalization than positive generalization, consistent with interference).

bioRxiv preprint doi: https://doi.org/10.1101/2024.09.29.615736; this version posted October 13, 2024. The copyright holder for this preprint (which was not certified by peer review) is the author/funder, who has granted bioRxiv a license to display the preprint in perpetuity. It is made available under aCC-BY 4.0 International license.

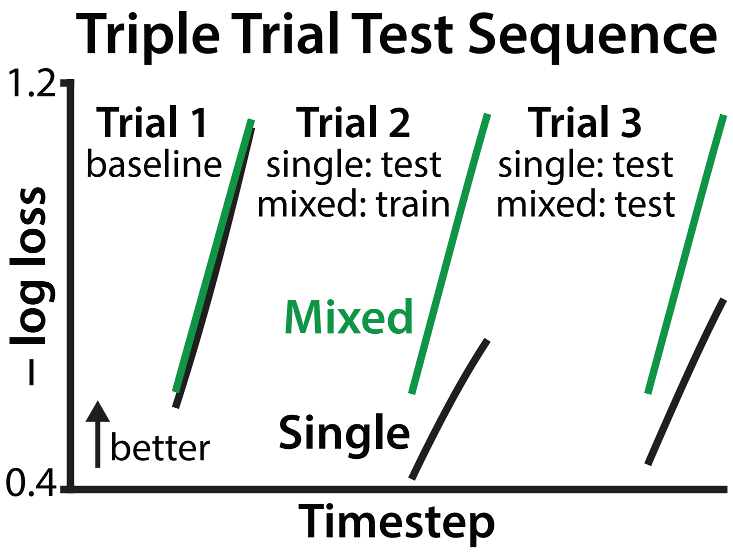


**Fig. S5.** Sequence generalization. Average test loss across timesteps within a 'triple trial'. Networks were trained on single trials (single networks) or single and double trials (mixed networks), and then tested on this three-trial sequence. SEM smaller than line thickness.

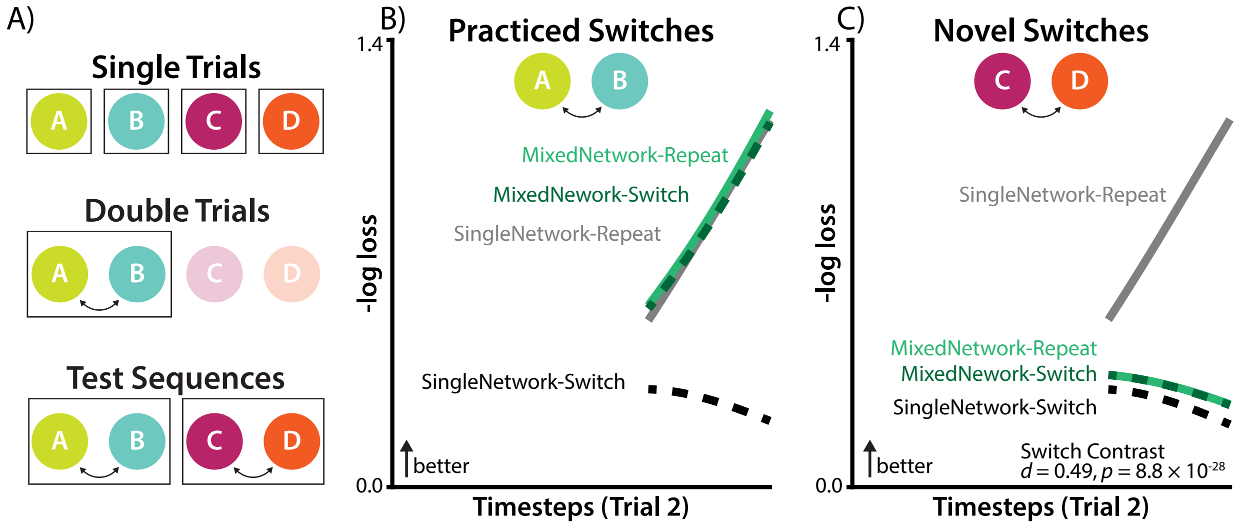


**Fig. S6.** Task generalization. **A)** We trained GRUs on a modified version of our protocol. In single trials, networks learned four different tasks. In double trials, networks practiced switching between two of the tasks. In test (shown in A and B), we examined switching performance between the practiced and novel task pairs. As in our main experiment, single networks performed only single trials (1000 epochs), and mixed networks were trained on 90% single trials and 10% double trials. **B)** The mixed network shows great performance on task transitions that it had practiced, it (as good at switching as the single network is at repeating). This is similar to the results Figure 4F (shown fore each condition, rather than as contrasts). **C)** On novel task transitions, the mixed network still shows lower switch costs and better performance on switch trials. Note that it has worse performance on repeat trials, likely due to these networks having less experience with C and D tasks (and/or catastrophic interference)

bioRxiv preprint doi: https://doi.org/10.1101/2024.09.29.615736; this version posted October 13, 2024. The copyright holder for this preprint (which was not certified by peer review) is the author/funder, who has granted bioRxiv a license to display the preprint in perpetuity. It is made available under aCC-BY 4.0 International license.

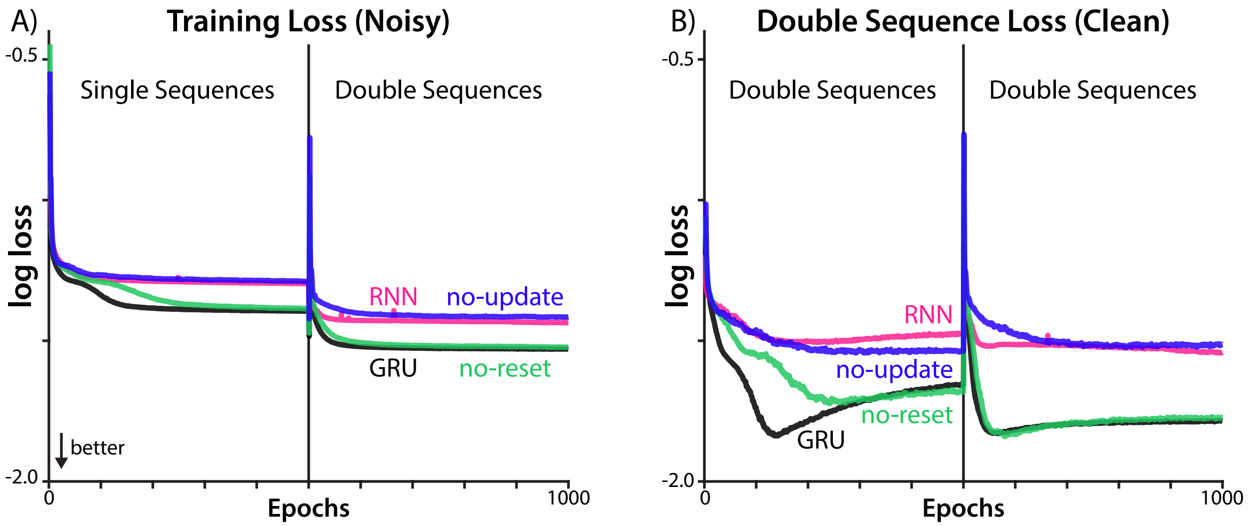


**Fig. S7.** Gate Ablation. We trained models with all gates ('GRU', black), fixed-open reset gates ('no-reset', green), fixed-open update gates ('no-update', blue), and classic Elman RNNs ('RNN', pink). All models are matched for the number of parameters (see Methods). The first epoch is removed to improve visualization. Note that we plot loss (instead of -loss) to follow convention. **A)** We train networks on single trials for 500 epochs, and then double trials for 500 epochs. During this training, noise was added to inputs to encourage the learning of a robust policy. During learning, GRU and no-reset have similar learning trajectories, with better performance (lower loss) than no-update or RNN models. **B)** Network loss was also tested on double trials at every epoch, without input noise. Here too, the GRU and no-reset show much stronger gains in performance from experience with the double trials. Note that GRU test loss during the single trials is increasing, suggesting that this is in an over-trained regime.

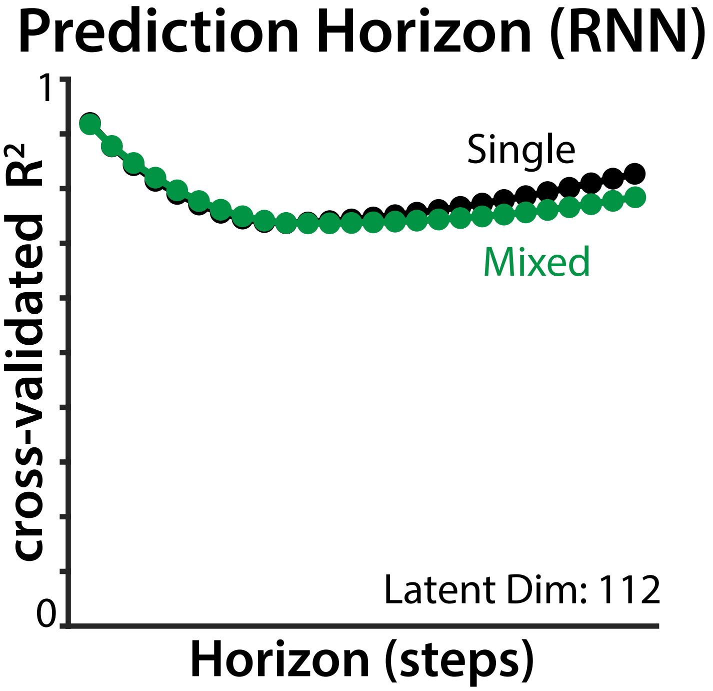


**Fig. S8.** Prediction horizon for SSAs fit to RNNs. SSA had similarly good predictive accuracy when fit to either single or mixed networks had. This high predictive accuracy was present both at the next timestep (training objective) and at distant temporal lags (25 timesteps is more than half of the epoch).
